# Insulin-like growth factor 1 receptor mediates photoreceptor neuroprotection

**DOI:** 10.1038/s41419-022-05074-3

**Published:** 2022-07-15

**Authors:** Ammaji Rajala, Kenneth Teel, Mohd A. Bhat, Albert Batushansky, Timothy M. Griffin, Lindsey Purcell, Raju V. S. Rajala

**Affiliations:** 1 Department of Ophthalmology, University of Oklahoma Health Sciences Center, Oklahoma City, OK 73104 USA; 2 Dean McGee Eye Institute, Oklahoma City, OK 73104 USA; 3 Oklahoma Medical Research Foundation, Oklahoma City, OK 73014 USA; 4 Department of Physiology, University of Oklahoma Health Sciences Center, Oklahoma City, OK 73104 USA; 5 Department of Cell Biology, University of Oklahoma Health Sciences Center, Oklahoma City, OK 73104 USA

**Keywords:** Retina, Growth factor signalling

## Abstract

Insulin-like growth factor I (IGF-1) is a neurotrophic factor and is the ligand for insulin-like growth factor 1 receptor (IGF-1R). Reduced expression of IGF-1 has been reported to cause deafness, mental retardation, postnatal growth failure, and microcephaly. IGF-1R is expressed in the retina and photoreceptor neurons; however, its functional role is not known. Global IGF-1 KO mice have age-related vision loss. We determined that conditional deletion of IGF-1R in photoreceptors and pan-retinal cells produces age-related visual function loss and retinal degeneration. Retinal pigment epithelial cell-secreted IGF-1 may be a source for IGF-1R activation in the retina. Altered retinal, fatty acid, and phosphoinositide metabolism are observed in photoreceptor and retinal cells lacking IGF-1R. Our results suggest that the IGF-1R pathway is indispensable for photoreceptor survival, and activation of IGF-1R may be an essential element of photoreceptor and retinal neuroprotection.

## Introduction

Insulin-like growth factor-1 (IGF-1), the ligand for insulin-like growth factor-1 receptor (IGF-1R), is a neurotrophic factor [1, 2]. In humans, IGF-1 deficiency caused by homozygous mutations in the IGF1 causes microcephaly, mental retardation, deafness, and postnatal growth failure, confirming the essential role of IGF-1 as a neurotrophic factor [1, 2]. Global IGF-1 KO mice have age-related vision loss in addition to congenital deafness [3]. Because of these properties, IGF-1 has been used to counteract neurodegeneration in experimental animal models of brain injury [4] and retinal neurodegeneration [5–8]. Our work over the past decade showed that the proteins that cause cancer (oncogenes) are neuroprotective [9–13] to post-mitotic photoreceptor cells, whereas anti-oncogenes inhibit tumor progression and promote retinal degeneration [14–16]. Furthermore, other laboratories have observed that proteins that promote tumor cell progression prevent retinal degeneration [17, 18]. Several neuroprotective pathways have been identified in the retina. These pathways are active under stress-induced conditions [10, 19–21], suggesting that other pathways might provide neuroprotection under normal conditions. Recently, exercise-induced retinal neuroprotection has been reported [22]. Progress in the identification of these pathways is slow due to the lack of a thorough understanding of these complex neuroprotective signaling pathways. IGF-1R is a receptor tyrosine kinase that mediates the actions of IGF1, which binds with high affinity, whereas IGF2 and insulin bind to this receptor with low affinity [23]. In the present study, we report the expression of IGF-1R and its functional role in the retina and photoreceptor cells.

## Results

### Generation of rod-specific IGF-1R KO mice

Some null mutants for the IGF-1R gene die of respiratory failure at birth, and those that do survive exhibit a severe growth deficiency (45% normal size) [24, 25]. To overcome postnatal developmental complications and determine the cell-specific role of IGF-1R, we generated rod photoreceptor-specific IGF-1R KO (abbreviated as ^*rod*^*Igf-1r*^*−/−*^) mice. To generate rod-specific conditional IGF-1R KO mice, we mated homozygous floxed IGF-1R mice with mice expressing Cre-recombinase under the control of rhodopsin promoter (i75-Cre) [26]. The resultant mice were heterozygous for IGF-1R. The Cre-carrying IGF-1R heterozygous mice were backcrossed with homozygous IGF-1R floxed mice. This breeding resulted in Cre-carrying homozygous IGF-1R floxed mice (^*rod*^*Igf-1r*^*−/−*^) and homozygous IGF-1R floxed mice (wild-type control).

### Characterization of rod-specific IGF-1R KO mice

**Fig. 1:**
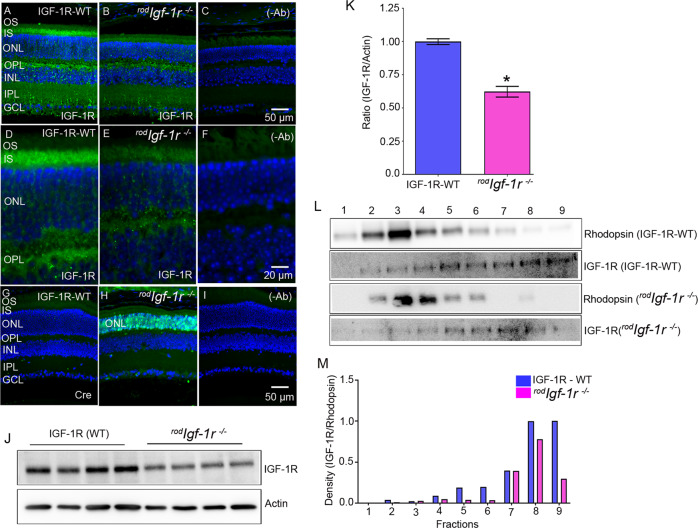
Expression of IGF-1R in wild-type and ^*rod*^*Igf-1r*^*−/−*^ mice. Prefer-fixed sections of 2-month-old WT (**A**, **D**, **G**) and ^*rod*^*Igf-1r*^*−/−*^ (**B**, **E**, **H**) mouse retinas were subjected to immunofluorescence with the IGF-1R (**A**, **B**, **D**, **E**) and Cre (**G**, **H**) antibodies. The sections were imaged at 20× (**A**–**C**) and 60× (**D**–**F**). **C**, **F**, and **I** These represent the omission of primary antibodies. OS, outer segments; IS, inner segments; ONL, outer nuclear layer; OPL, outer plexiform layer; INL, inner nuclear layer; IPL, inner plexiform layer; GCL, ganglion cell layer. Retinal lysates from IGF-1R-WT and ^*rod*^*Igf-1r*^*−/−*^ mice were immunoblotted with IGF-1R and actin antibodies (**J**). Densitometric analysis of IGF-1R from whole retinas of WT and ^*rod*^*Igf-1r*^*−/−*^ mice was normalized to actin (**K**). Data are mean ± *SEM* (*n* = 4). An unpaired parametric test with Welch’s correction was used to determine the statistical significance. *p < 0.0006. Tangential serial cryosections from 2-month-old IGF-1R-WT and ^*rod*^*Igf-1r*^*−/−*^ mice were subjected to immunoblot analysis with rhodopsin and IGF-1R antibodies (**L**). Densitometric analysis of IGF-1R/rhodopsin (**M**).

Wild-type and ^*rod*^*Igf-1r*^*−/−*^ mouse retina sections were stained with IGF-1R and Cre antibodies. The results indicated the loss of IGF1R in the rod inner segment (RIS) and outer plexiform (OPL) layers of the retina compared with wild-type mice (Fig. 1A–F). Our results also showed the proper targeting of Cre-recombinase to rod photoreceptor cells, as we observed the expression of Cre in the outer nuclear layer (ONL) of ^*rod*^*Igf-1r*^*−/−*^ mice, but not in wild-type mice (Fig. 1G–I). These experiments suggest the Cre-mediated deletion of IGF-1R in rod photoreceptor cells. Immunoblot analysis shows a significant decrease in the levels of IGF-1R in ^*rod*^*Igf-1r*^*−/−*^ mouse retinas compared with wild-type retinas (Fig. 1J, K). We carried out serial-tangential cryosectioning with immunoblotting [27] to examine the expression of IGF-1R in rod and cone photoreceptors of wild-type and ^*rod*^*Igf-1r*^*−/−*^ mice, using rhodopsin as a photoreceptor marker (Fig. 1L, M). The sections presented in panel L include both outer segments and inner segments. The peak IGF-1R presence in ^*rod*^*Igf-1r*^*−/−*^ mice can be seen between fractions 5 and 7, whereas the IGF-1R protein presence in littermate controls is seen in fractions 3–9. Furthermore, the IGF-1R levels in peak rhodopsin fractions are much lower than in other fractions (5–9). Our results showed reduced expression of IGF-1R in the photoreceptors of ^*rod*^*Igf-1r*^*−/−*^ mice, compared with wild-type mice. The residual expression of IGF-1R in the ^*rod*^*Igf-1r*^*−/−*^ mouse photoreceptors could most likely come from cones or incomplete recombination of Cre-mediated excision of floxed IGF-1R allele.

### Functional characterization of ^*rod*^*Igf-1r*^*−/−*^mice

**Fig. 2:**
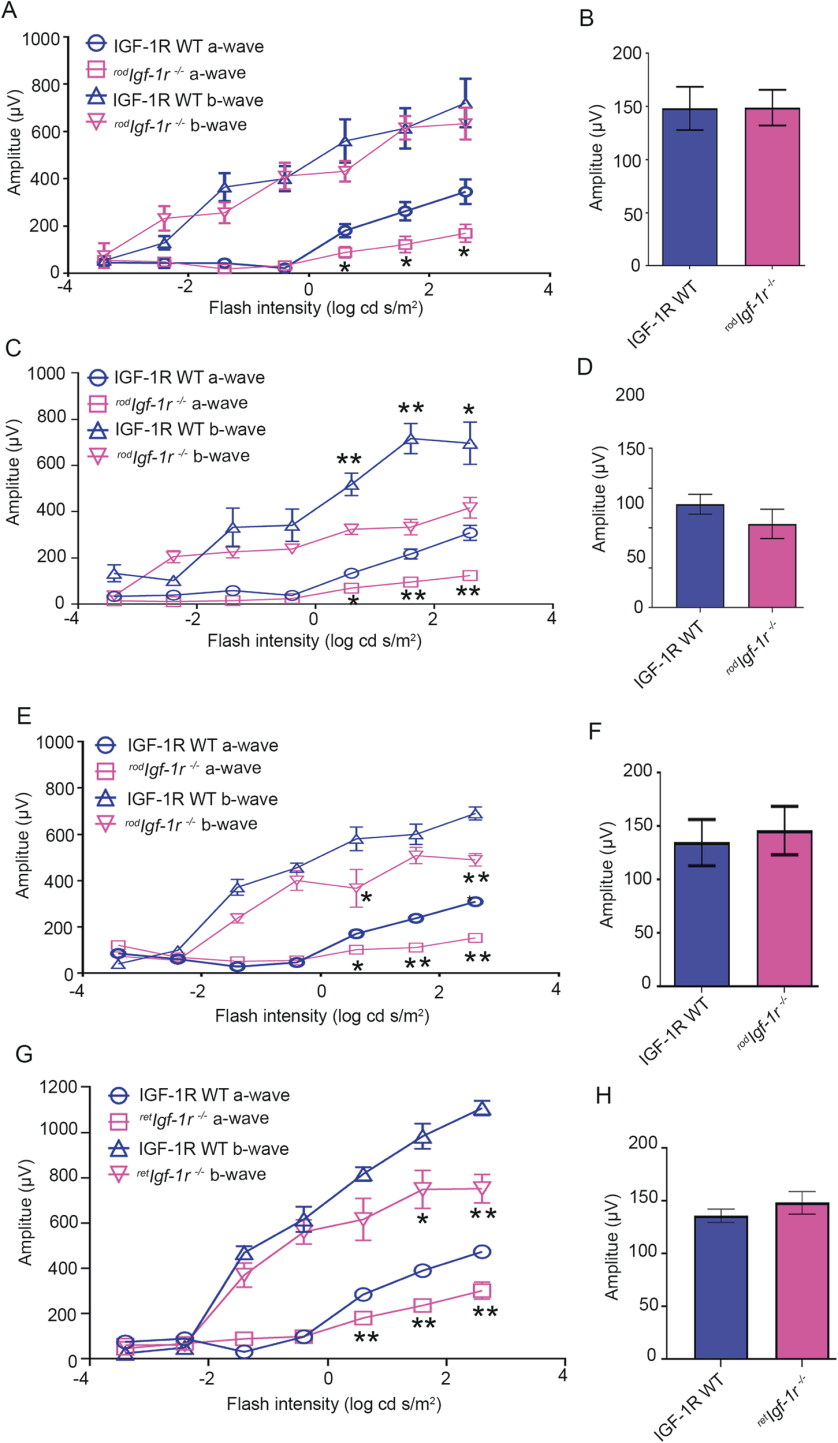
Characterization and retinal function of ^*rod*^*Igf-1r*^*−/−*^ mice. Scotopic a-wave (**A**, **C**, **E**), scotopic b-wave (**A**, **C**, **E**), and photopic b-wave (**B**, **D**, **F**) analyses were performed on 1-month-old (**A**, **B**), 4-month-old (**C**, **D**), and 6-month-old (**E**, **F**) IGF-1R-WT and ^*rod*^*Igf-1r*^*−/−*^ mice. Six-week-old IGF-1R-WT and ^*ret*^*Igf-1r*^*−/−*^ mice were subjected to ERG and we measured scotopic a-wave, scotopic b-wave (**G**), and photopic b-wave (**H**) amplitudes. Scotopic a-wave and scotopic b-wave amplitudes were carried out at different flash intensities (−3.4, −2.4, −1.4, −0.4, 0.6, 1.6, and 2.6 log cd s/m^2^), whereas photopic b-wave amplitudes were performed at a flash intensity of 3.3 log cd s/m^2^. Data are mean ± *SEM* (*n* = 8). Two-way ANOVA was used to determine the statistical significance. The data were corrected for multiple comparisons by controlling the False Discovery Rate using a two-stage linear step-up procedure of the Benjamini, Krieger, and Yekutieli test. *p < 0.05; **p < 0.001.

Retinal function was measured in 1-, 4-, and 6-month-old ^*rod*^*Igf-1r*^*−/−*^ and wild-type littermates. At 1 month, the ^*rod*^*Igf-1r*^*−/−*^ mice showed significantly reduced scotopic a-wave amplitude compared with wild-type mice (Fig. 2A). The scotopic a-wave amplitude progressively declined as the age of ^*rod*^*Igf-1r*^*−/−*^ mice increased (4 months and 6 months) compared with wild-type mice (Fig. 2C, E). There was no significant difference in the scotopic b-wave amplitude at one month between wild-type and ^*rod*^*Igf-1r*^*−/−*^ mice (Fig. 2A). However, as the age of the mice increased, there was a significant decrease in scotopic b-wave amplitude in ^*rod*^*Igf-1r*^*−/−*^ mice at 4 and 6 months compared with wild-type mice (Fig. 2C, E). The difference in photopic b-wave amplitudes was not significant between wild-type and ^*rod*^*Igf-1r*^*−/−*^ mice at each time point (Fig. 2B, D, and F). These experiments suggest that IGF-1R is essential for rod photoreceptor functions.

We generated retina-specific IGF-1R KO mice under the control of a homeobox gene, Chx10 [28]. Six-week-old Chx10-Cre-IGF-1R KO (abbreviated as ^*ret*^*Igf-1r*^*−/−*^) mice exhibited a significant loss of scotopic a-wave and scotopic b-wave amplitudes compared with six-week-old wild-type mice (Fig. 2G). There was no significant difference in the photopic b-wave amplitudes between ^*ret*^*Igf-1r*^*−/−*^ and wild-type mice (Fig. 2H). These observations suggest that loss of IGF-1R in other retinal cells exacerbates the loss of rod function compared with the loss of IGF-1R in rod cells.

### Structural characterization of ^*rod*^*Igf-1r*^*−/−*^ mice

**Fig. 3:**
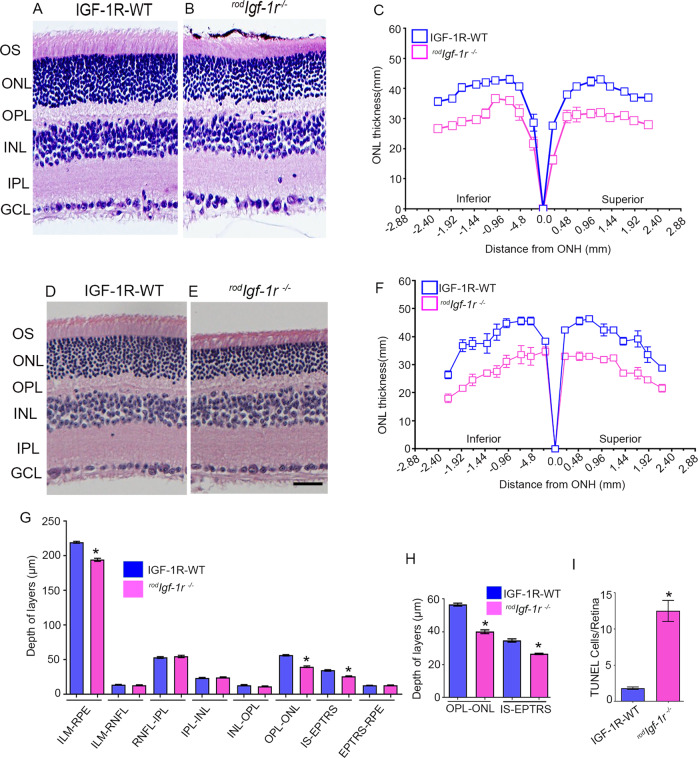
Structural characterization of ^*rod*^*Igf-1r*^*−/−*^mice. Prefer-fixed sections of 2-month-old (**A**, **B**) and 4-month-old (**D**, **E**) IGF-1R-WT and ^*rod*^*Igf-1r*^*−/−*^ mouse retinas were stained with hematoxylin and eosin and examined for morphology. Plots of total retinal thickness of 2-month-old (**C**) and 4-month-old (**F**) IGF-1R-WT and ^*rod*^*Igf-1r*^*−/−*^ mouse retinas were measured from the optic nerve head (ONH) in the inferior and superior regions of the retinas of IGF-1R-WT and ^*rod*^*Igf-1r*^*−/−*^ mice. Data are mean ± *SEM* (*n* = 6). Two-way ANOVA with the Bonferroni test was used to determine the statistical significance. There was a significantly greater loss of rod nuclei in both hemispheres of the ^*rod*^*Igf-1r*^*−/−*^ mouse retinas than in the IGF-1R-WT retinas (*p* < 0.001). OCT showed decreased OPL-ONL and IS-EPTRS in ^*rod*^*Igf-1r*^*−/−*^ mice (**G**, **H**). Data are mean ± *SEM* (*n* = 8). An unpaired *t* test with Welch’s correction was used to determine the significance. *p < 0.001. In situ localization of apoptosis using TUNEL staining (**I**). Data are mean ± *SEM* (*n* = 8). An unpaired nonparametric Mann-Whitney test was used to determine the significance. *p < 0.001.

To determine whether loss of IGF-1R in rods affects retinal structure, we stained retinal sections from 2-month-old ^*rod*^*Igf-1r*^*−/−*^ and wild-type littermate mice with hematoxylin and eosin and examined the morphology. The morphology in ^*rod*^*Igf-1r*^*−/−*^ retina appeared to be thinning of the photoreceptor outer segments (POS) and outer nuclear layer (ONL) compared with wild-type mouse retina (Fig. 3A, B). Quantitative analysis of ONL thickness measured at 0.24-mm intervals from the optic nerve head to the inferior and superior ora serrata indicated that there was a significant loss of rod nuclei as an indicator of photoreceptor degeneration in ^*rod*^*Igf-1r*^*−/−*^ mice compared with wild-type mice (Fig. 3C). These observations suggest that IGF-1R is essential for the maintenance of photoreceptor structure. Four-month-old ^*rod*^*Igf-1r*^*−/−*^ mice had reduced ONL and POS compared with wild-type mice (Fig. 3D, E). Quantitative analysis indicated that there was a significant loss of rod nuclei as an indicator of photoreceptor degeneration in ^*rod*^*Igf-1r*^*−/−*^ mice compared with wild-type mice (Fig. 3F). The optical coherence tomography analysis indicated a significant loss of OPL-ONL and IS-EPTRS (photoreceptor tips) layer thickness in ^*rod*^*Igf-1r*^*−/−*^ mice compared with wild-type mice (Fig. 3G, H). Wild-type and ^*rod*^*Igf-1r*^*−/−*^ mouse retina sections probed with TUNEL staining indicated increased TUNEL-positive staining in ^*rod*^*Igf-1r*^*−/−*^ mice compared with wild-type mice (Fig. 3I), suggesting that loss of IGF-1R in photoreceptor cells causes rod degeneration.

### Characterization of retina-specific IGF-1R KO mice

**Fig. 4:**
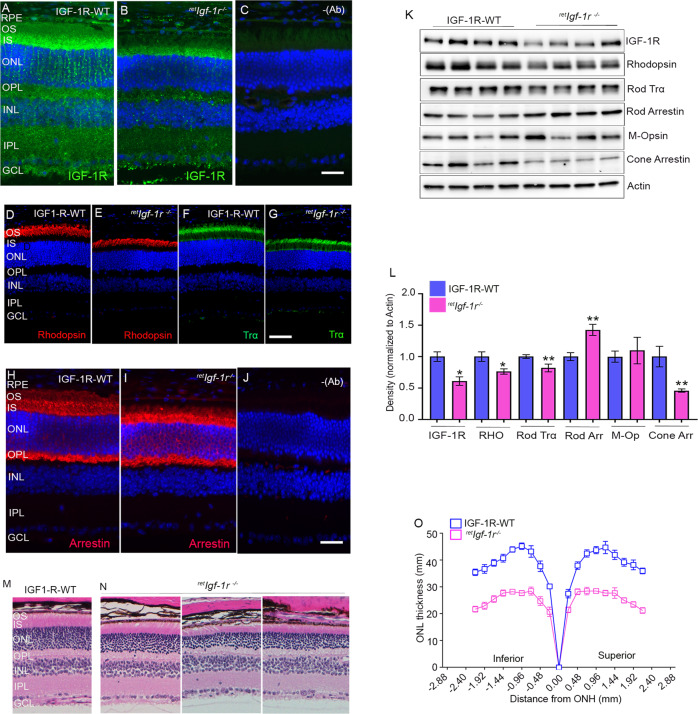
Expression of IGF-1R and structural characterization of ^*ret*^*Igf-1r*^*−/−*^mice. Prefer-fixed sections of six-week-old IGF-1R-WT and ^*ret*^*Igf-1r*^*−/−*^ mouse retinas were subjected to immunofluorescence with IGF-1R (**A**, **B**), rhodopsin (**D**, **E**), rod-Trα (**F**, **G**) and rod arrestin (**H**, **I**) antibodies. (**C** and **J**) represent the omission of primary antibodies. Scale bar = 50 µm. RPE, retinal pigment epithelium; OS, outer segments; IS, inner segments; ONL, outer nuclear layer; OPL, outer plexiform layer; INL, inner nuclear layer; IPL, inner plexiform layer; GCL, ganglion cell layer. Retina lysates from the IGF-1R-WT and ^*ret*^*Igf-1r*^*−/−*^ mice were subjected to immunoblot analysis with IGF-1R, rhodopsin, rod-Trα, rod arrestin, M-opsin, cone-arrestin, and actin antibodies (**K**). Densitometric analysis of retinal proteins normalized to actin (**L**). Data are mean ± *SEM* (*n* = 4). An unpaired nonparametric Mann-Whitney test was used to determine the significance. *p < 0.05; **p < 0.02. Sections from 6-week-old IGF-1R-WT (**M**) and ^*ret*^*Igf-1r*^*−/−*^ (**N**) mice were hematoxylin and eosin-stained and examined for morphology. Plots of total retinal thickness were measured from the optic nerve head (ONH) in the inferior and superior regions of the retinas of 2-month-old IGF-1R-WT and ^*ret*^*Igf-1r*^*−/−*^ mice (**O**). Data are mean ± *SEM* (*n* = 8). Two-way ANOVA was used, corrected for multiple comparisons by controlling the False Discovery Rate using a two-stage linear step-up procedure of the Benjamini, Krieger, and Yekutieli test. There was a significantly greater loss of rod nuclei in both hemispheres of the ^*ret*^*Igf-1r*^*−/−*^ mouse retinas than in the IGF-1R-WT retinas (*p* < 0.001).

Six-week-old wild-type and ^*ret*^*Igf-1r*^*−/−*^ mouse retina sections were stained with IGF-1R, rhodopsin, rod-Trα, and rod arrestin antibodies. The results indicated the significant loss of IGF1R in OPL, inner plexiform layer (IPL), and ganglion cell layer, and some residual IGF-1R expression in the RIS (Fig. 4A–C). The expression of rhodopsin and rod-Trα were significantly reduced in ^*ret*^*Igf-1r*^*−/−*^ mice compared with wild-type mice (Fig. 4D–G). It is interesting to note that arrestin expression in ^*ret*^*Igf-1r*^*-/*^ appears to be higher in IS, ONL, and OPL layers of the retina (Fig. 4 H–J). Immunoblot analysis showed significantly reduced levels of IGF-1R, rhodopsin, transducin alpha (Trα), and cone arrestin (Fig. 4K, L). The levels of rod arrestin were significantly higher in ^*ret*^*Igf-1r*^*−/−*^ mice than in wild-type mice (Fig. 4K, L). Quantitative analysis of ONL thickness showed a significant loss of rod nuclei in ^*ret*^*Igf-1r*^*−/−*^ mice compared with wild-type mice (Fig. 4M–O). These findings show that loss of IGF-1R in pan-retinal cells leads to retinal degeneration, suggesting that IGF-1R is essential for retinal cell survival.

### Effect of circulating IGF-1 on the retina

**Fig. 5:**
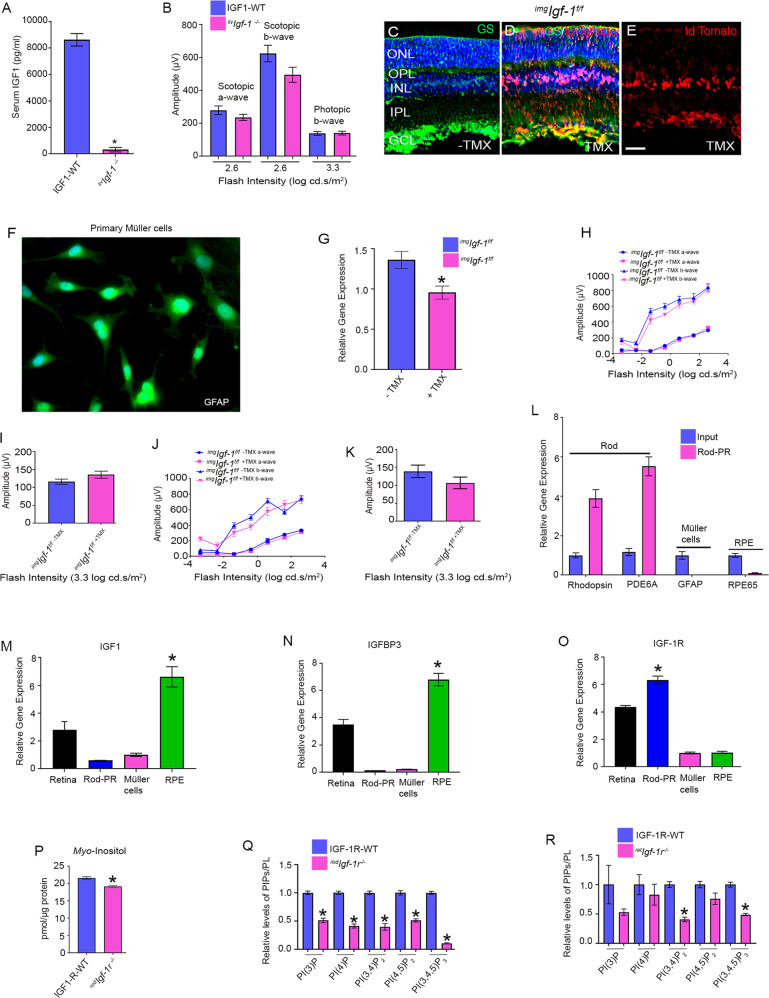
Biochemical and functional studies of IGF-1 and IGF-1R. Serum IGF-1 levels were measured from 2-month-old IGF-1-WT and ^*liv*^*Igf-1*^*−/−*^ mice (**A**). Data are mean ± *SEM*, (*n* = 5). Welch’s *t*-test was used to determine the significance of these two groups. *p < 0.01. Eight-week-old IGF-1-WT and ^*liv*^*Igf-1*^*−/−*^ mice were subjected to ERG and we measured scotopic a-wave, scotopic b-wave, and photopic b-wave amplitudes (**B**). Data are mean ± *SEM*, (*n* = 12). Unpaired nonparametric Mann-Whitney test was used to determine the significance between these two groups, but there was no significant difference. Tamoxifen-inducible GLAST-Cre mediated expression of tdTomato. Two-month-old Ai9 mice expressed robust tdTomato fluorescence following TMX-inducible Cre-mediated recombination. GLAST-tdTomato mice induced with TMX (**D**, **E**) and without TMX (**C**). Sections were stained with GS (green) (**C**, **D**). Red represents the intrinsic fluorescence. **E** This is the same as **D**, without DAPI. Scale bar = 50 µm. Primary Müller cell cultures were prepared from postnatal day 7 (P7) C57Bl6 pups and immunostained with GFAP antibody (**F**). Primary Müller cell cultures were prepared from 2-month-old ^*img*^*Igf-1*^*−/−*^ mice injected with either peanut oil or TMX. RNA was isolated from these cells and converted to cDNA followed by qRT-PCR with IGF-1 primers and the data were normalized to actin (**G**). Data are mean ± *SEM* (*n* = 3), *p < 0.038. ^*img*^*Igf-1*^*−/−*^ mice with and without TMX-treatment were examined for function at 3.5 (**H**, **I**) and 5.5 (**J**, **K**) months and we measured scotopic a-wave, scotopic b-wave (**H**, **J**), and photopic b-wave amplitudes (**I**, **K**). Data are mean ± *SEM* (*n* = 18). Two-way ANOVA was used, corrected for multiple comparisons by controlling the False Discovery Rate using a two-stage linear step-up procedure of the Benjamini, Krieger, and Yekutieli test. There was no significant difference between these two groups. qRT-PCR analysis of the mRNA showed enrichment of rod-specific transcripts and depletion of transcripts from Müller cells and RPE (**L**). Data are mean ± *SEM* (*n* = 3). Multiple unpaired *t*-tests were used to compare the significance between the retina and rod enriched transcripts. *p < 0.0001. qPCR analysis of IGF-1 (**M**), IGFBP3 (**N**), and IGF-1R (**O**) expression was examined from 2-month-old C57Bl6 mouse retina, enriched photoreceptor cells, mouse RPE, and P7 mouse primary Müller cells. Data are mean ± *SEM* (*n* = 3). The statistical difference between various retinal cells was assessed by using one-way ANOVA. *p < 0.0001. *Myo*-inositol levels were determined from 6-week-old IGF-1R-WT and ^*rod*^*Igf-1r*^*−/−*^ mouse retinas (**P**). Data are mean ± *SEM* (*n* = 9). An unpaired *t*-test with Welch’s correction was used to determine the significance between these two groups. *p < 0.0001. Phosphoinositides levels in 3-month-old IGF-1R-WT, ^*rod*^*Igf-1r*^*−/−*^ (**Q**) and ^*ret*^*Igf-1r*^*−/−*^ (**R**) mice were measured using an ELISA assay employing PI(3)P, PI(4)P, PI(3,4)P_2_, PI(4,5)P_2_, and PI(3,4,5)P_3_. Data are mean ± *SEM* (*n* = 3). Retinas were pooled from 5 mice and 15 mice were used for this experiment. An unpaired *t* test with Welch’s correction was used to determine the significance between these two groups. *p < 0.0001.

IGF-1R is activated by the ligand IGF-1, and the liver is the major site of IGF-1 production [29]. To generate liver-specific IGF-1 KO mice, we mated floxed IGF-1 mice with mice carrying Cre-recombinase under the control of albumin promoter. Circulating IGF-1 levels were measured in serum from 8-week-old wild-type and liver-specific IGF-1 KO (abbreviated as ^*liv*^*Igf-1*^*−/−*^) mice. Loss of IGF-1 in the liver resulted in a >95% decrease in IGF-1 in circulation compared with wild-type mice (Fig. 5A). We measured retinal function in eight-week-old wild-type and ^*liv*^*Igf-1*^*−/−*^ mice, and the loss of IGF-1 in the liver had no effect on retinal function (Fig. 5B). These results suggest that IGF-1R activation in the retina may occur through IGF-1 produced outside the liver.

### Müller cell-specific deletion of IGF-1 does not affect retina function

IGF-1 expression has been observed in Müller glia [30]. Using Müller cell-specific glial high-affinity glutamate transporter (GLAST)-Cre mice, we examined whether Müller cell IGF-1 serves as a ligand for photoreceptor IGF-1R [31]. To test the efficiency and specificity of Müller cell-specific GLAST-Cre mice [31], we mated this line with an Ai9 reporter line having a *loxP*-flanked STOP cassette preventing transcription of a CAG promoter-driven red fluorescent protein variant (tdTomato) inserted into the *Gt(ROSA)26Sor* locus [32]. GLAST-Cre-Ai9 mice were orally gavaged with peanut oil (control) or 1 mg tamoxifen (TMX) (dissolved in peanut oil) every alternate day for 3 days. We found robust tdTomato fluorescence in retinas from mice gavaged with TMX (Fig. 5D, E). There was no expression in the absence of TMX (Fig. 5C). The Müller cell marker glutamine synthetase colocalized with tdTomato, indicating Müller cell-specific expression of tdTomato (Fig. 5D). We used GLAST-Cre mice to delete IGF-1(^*img*^*Igf-1*^−/−^) in Müller cells. Primary Müller cells were isolated from P7 C57Bl6 pups and stained with GFAP to confirm that they were indeed Müller cells (Fig. 5F). Primary Müller cells were prepared from 2-month-old ^*img*^*Igf-1*^−/−^ mice that were gavaged with either TMX or peanut oil, and IGF-1 expression was examined with qRT-PCR. Our results indicated significantly reduced levels of IGF-1 in ^*img*^*Igf-1*^−/−^ mice treated with TMX compared with mice treated with peanut oil (Fig. 5G). Examination of retinal function in these mice at 3.5 (Fig. 5H, I) and 5.5 (Fig. 5J, K) months of age indicated that loss of IGF-1 in the Müller cells had no effect on retinal function (Fig. 5H–K), suggesting that Müller cells may not be the source of IGF-1 production.

### Expression of IGF-1, IGF-1R, and IGFBP3 in the retina, rod photoreceptor cells, retinal pigment epithelium, and Müller cells

The RiboTag mouse carries a ribosomal protein gene (*Rpl22*) with a floxed C-terminal exon followed by an identical exon tagged with hemagglutinin (HA) [33]. When RiboTag is crossed to a mouse expressing a cell-type-specific Cre recombinase, expression of the HA-epitope-tagged protein is activated in the cell type of interest [33]. We bred rhodopsin-Cre with Rpl22 floxed mice. Polyribosomal immunoprecipitation with HA-antibody recovered ribosomal-associated mRNA. qRT-PCR analysis of the mRNA showed enrichment of rod-specific rhodopsin and phosphodiesterase 6α transcripts (Fig. 5L). The mRNA isolated from rod cells by polyribosomal immunoprecipitation, total retina, mouse RPE, and primary Müller cells from P7 pups were reverse transcribed to complementary cDNA and subjected to qRT-PCR with primers specific to IGF-1, IGFBP3, and IGF-1R. We normalized the expression to β-actin. Our results indicated that the expression of IGF-1 and IGFBP3 were significantly higher in RPE than in retina, rod photoreceptors, and Müller cells (Fig. 5M, N). The expression of IGF-1R was significantly higher in rod photoreceptor cells than in retina, RPE, and Müller cells (Fig. 5O). These observations suggest that IGF-1R in the rods may be activated by RPE-secreted IGF-1 through paracrine signaling.

### Effect of loss of IGF-1R on phosphoinositides

A *myo*-inositol pool is utilized for phosphatidylinositol (PI) synthesis [34]. We found significantly decreased levels of *myo*-inositol in ^*rod*^*Igf-1r*^*−/−*^ mice compared with IGF-1R wild-type mice (Fig. 5P). The parent molecule PI undergoes phosphorylation, and the phosphorylated products are further phosphorylated and dephosphorylated by phosphoinositide kinases and phosphoinositide phosphatases. These reactions give rise to seven distinct phosphorylated phosphoinositides (PIPs). In the present study, we measured five PIPs: PI(3)P, PI(4)P, PI(3,4)P_2_, PI(4,5)P_2,_ and PI(3,4,5)P_3_. Our results indicated a significant decrease in the levels of five PIPs in ^*rod*^*Igf-1r*^*−/−*^ mice compared with those in rods from wild-type mice (Fig. 5Q). In ^*ret*^*Igf-1r*^*−/−*^ mouse retinas, we found significantly reduced levels of PI(3,4)P_2_ and PI(3,4,5)P_3_ compared with wild-type mice (Fig. 5R). The levels of PI(3)P and PI(4,5)P2 were also decreased in ^*ret*^*Igf-1r*^*−/−*^ mice compared with wild-type mice. However, this difference was not statistically significant. These findings suggest that IGF-1R regulates phosphoinositide metabolism.

### Altered retinal metabolism in rods lacking IGF-1R

**Fig. 6:**
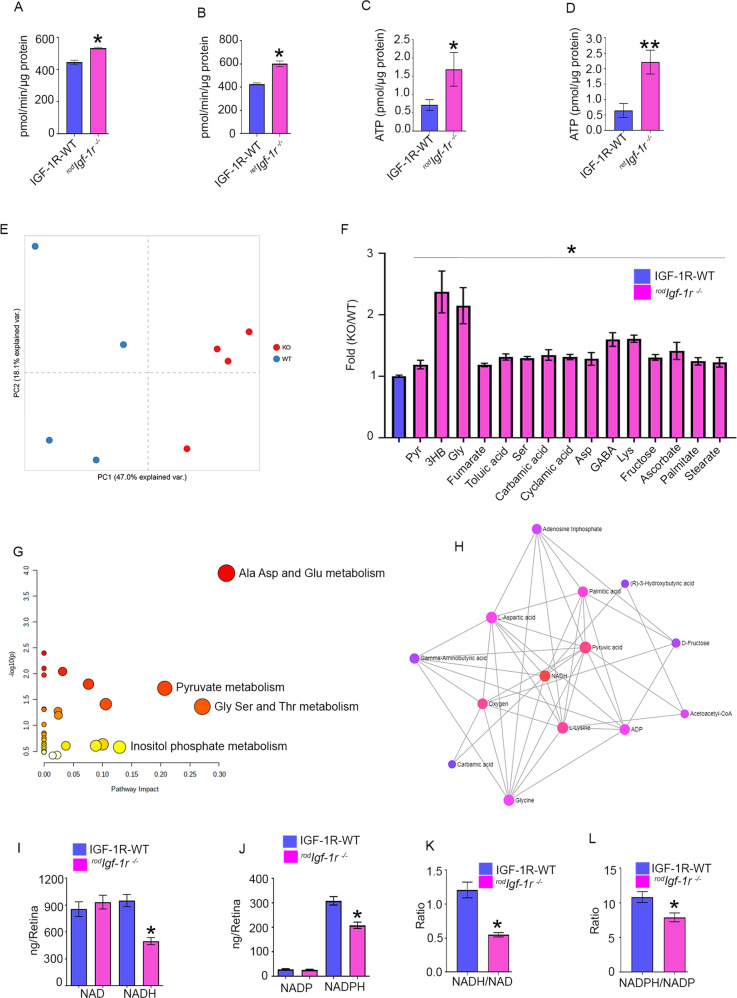
Retinal metabolism in ^*rod*^*Igf-1r*^*−/−*^mice. Pyruvate kinase activity was measured from 2-month-old IGF-1R-WT, ^*rod*^*Igf-1r*^*−/−*^ (**A**) and ^*ret*^*Igf-1r*^*−/−*^ (**B**) mice. Data are mean ± *SEM* (*n* = 12). Unpaired nonparametric Mann-Whitney test was used to determine the significance between these two groups. *p < 0.0001. ATP levels were measured from 2-month-old IGF-1R-WT, ^*rod*^*Igf-1r*^*−/−*^ (**C**) and ^*ret*^*Igf-1r*^*−/−*^mice (**D**). Data are mean ± *SEM* (*n* = 4). Unpaired nonparametric Mann-Whitney test (**C**) and unpaired parametric test with Welch’s correction (**D**) were used to determine the significance. *p < 0.05, **p < 0.01. Principal com*p*onent analysis (PCA) of the samples based on 48 unique compounds demonstrated a clear separation between the tested groups (**E**). Steady-state level retinal metabolites were measured from 2-month-old IGF-1R-WT and ^*rod*^*Igf-1r*^*−/−*^ mouse retina (**F**) and data were subjected to a network (**G**) and interaction analysis (**H**). Data are mean ± *SEM* (*n* = 4). One-way ANOVA with the Kruskal-Wallis test was used to determine the significance. *p < 0.05. NAD, NADH (**I**), NADP, and NADPH (**J**) were measured from 2-month-old IGF-1R-WT mice and ^*rod*^*Igf-1r*^*−/−*^ mice, and NADH/NAD (**K**) and NADPH/NADP (**L**) ratios were calculated. Data are mean ± *SEM* (*n* = 9). Unpaired *t* test with Welch’s correctio*n* was used to de*t*ermine the significance between these two groups. *p < 0.0001.

We measured pyruvate kinase activity in ^*rod*^*Igf-1r*^*−/−*^ (Fig. 6A) and ^*ret*^*Igf-1r*^*−/−*^ (Fig. 6B) mouse retinas. Our results indicated significantly increased pyruvate kinase activity in both ^*rod*^*Igf-1r*^*−/−*^ and ^*ret*^*Igf-1r*^*−/−*^ mouse retinas compared with wild-type mouse retinas (Fig. 6A, B). These observations suggest that increased oxidative phosphorylation, not anabolic metabolism, might occur in ^*rod*^*Igf-1r*^*−/−*^ and ^*ret*^*Igf-1r*^*−/−*^ mice. Consistent with the increased pyruvate kinase activity in ^*rod*^*Igf-1r*^*−/−*^ and ^*ret*^*Igf-1r*^*−/−*^ mice, ATP levels in ^*rod*^*Igf-1r*^*−/−*^ (Fig. 6C) and ^*ret*^*Igf-1r*^*−/−*^ (Fig. 6) mice were significantly higher than levels in wild-type mice.

To determine the effect of loss of IGF-1R on retinal metabolism, we measured steady-state levels of metabolites in IGF-1R floxed and ^*rod*^*Igf-1r*^*−/−*^ mouse retinas. Principal component analysis (PCA) of the samples based on 48 unique compounds demonstrated a clear separation between the tested groups (Fig. 6E). Our analysis indicated that several metabolites, including pyruvate, 3-hydroxybutyrate, glycine, fumarate, toluic aid, serine, carbamic acid, cyclamic acid, aspartic acid, GABA, lysine, fructose, ascorbate, palmitate, and stearate, were significantly increased in ^*rod*^*Igf-1r*^*−/−*^ mouse retinas compared with wild-type mouse retinas (Fig. 6F). The pathway analysis indicated impactful alterations in 1) Ala, Asp, Glu, 2) Pyruvate, and 3) Gly, Ser, and Thr metabolism, and possible alternations in 4) inositol phosphate metabolism (Fig. 6G). We performed a metabolite-metabolite interaction analysis using MetaboAnalyst 5.0 (https://www.metaboanalyst.ca/) to identify possible functional relationships between altered metabolites in ^*rod*^*Igf-1r*^*−/−*^ mouse retinas. Associations from the metabolites were extracted from the STITCH database [35]. The resulting network graph had 398 nodes and 765 edges identifying potentially altered metabolites from the 15 “seed” metabolites identified by our GC-MS analysis (Table S1). We then converted the network to a minimum spanning network to identify alterations in other metabolites that GC analysis did not reveal. The minimum network analysis of the analyzed steady-state metabolites and their interaction indicated that potential changes are centered on NADH (betweenness 14.9) (Fig. 6H, Table S2). Furthermore, the minimum network encompassed metabolites and highlighted potential metabolic changes in oxygen, ATP/ADP, and acetoacetyl-CoA.

We measured reduced and oxidized forms of pyridine nucleotides (NAD, NADH, NADP, and NADPH) in 2-month-old wild-type and ^*rod*^*Igf-1r*^*−/−*^ mice. Our results indicated that there was no significant difference in the levels of oxidized forms of pyridine nucleotides NAD and NADP in wild-type and ^*rod*^*Igf-1r*^*−/−*^ mice (Fig. 6I, J). However, the levels of the reduced form of pyridine nucleotides, NADH and NADPH, were significantly lower in ^*rod*^*Igf-1r*^*−/−*^ mice than in wild-type mice (Fig. 6I, J). The ratios of NADH/NAD (Fig. 6K) and NADPH/NADP (Fig. 6L) were significantly reduced in ^*rod*^*Igf-1r*^*−/−*^ mice compared with wild-type mice, suggesting that IGF-1R signaling regulates the cellular redox, which may further promote photoreceptor survival and function. These studies suggest that IGF-1R is involved in the regulation of retinal metabolism.

### Fatty acid analysis and fatty acid oxidation in ^*rod*^*Igf-1r*^*−/−*^ mice

**Fig. 7:**
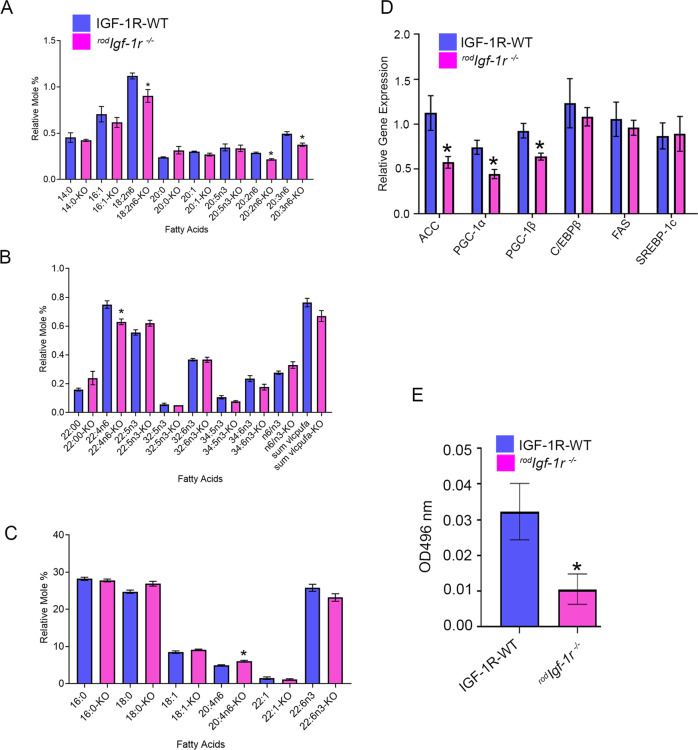
Fatty acid profile, fatty acid synthesis genes, and fatty acid oxidation in ^*rod*^*Igf-1r*^*−/−*^ retinas. Fatty acid profiles of two-month-old IGF-1R-WT and ^*rod*^*Igf-1r*^*−/−*^ mice were assessed (**A–C**), as described in the Materials and Methods section. Data are mean ± SEM (*n* = 3). An unpaired *t*-test with Welch’s correction was used to determine the significance between these two groups. *p < 0.05. qPCR analysis of fatty acid synthesis genes was performed using retinal cDNA prepared from 2-month-old IGF-1R-WT and ^*rod*^*Igf-1r*^*−/−*^ mouse RNA and the gene expression was normalized to 18S ribosomal RNA (**D**). Data are mean ± SEM (*n* = 3). Multiple unpaired *t*-tests were used to compare the significance between the groups. *p < 0.05. Fatty acid β-oxidation was measured in 2-month-old IGF-1R-WT and ^*rod*^*Igf-1r*^*−/−*^ mouse retinas (**E**). Data are mean ± SEM (*n* = 5). Unpaired nonparametric Mann-Whitney test was used to determine the significance. *p < 0.05.

The fatty acid profile was determined from 2-month-old wild-type and ^*rod*^*Igf-1r*^*−/−*^ mouse retinas. We examined the relative levels of 14:0, 16:0, 16:1, 18:0, 18:1, 18:2n6, 20:0, 20:1, 20:5n3, 20:2n6, 20:3n6, 20:4n6, 22:00, 22:1, 22:4n6, 22:5n3, 22:6n3, 32:5n3, 32:6n3, 34:5n3, and 34:6n3 fatty acids. Our analysis showed significantly reduced levels of 18:2n6, 20:2n6, 20:3n6, and 22:4n6 fatty acids in ^*rod*^*Igf-1r*^*−/−*^ mouse retina compared with wild-type retina (Fig. 7A–C). The levels of 20:4n6 fatty acid were significantly increased in ^*rod*^*Igf-1r*^*−/−*^ mouse retina compared with wild-type retina (Fig. 7C). We also examined the expression of genes involved in fatty acid synthesis: ACC, PGC-1α, PGC-1β, C/EBPβ, FAS, PGC1, and SREBP-1c. Our results indicated significantly reduced levels of ACC, PGC-1α, and PGC-1β in ^*rod*^*Igf-1r*^*−/−*^ mice compared with wild-type mice (Fig. 7D). The fatty acid β-oxidation was significantly decreased in ^*rod*^*Igf-1r*^*−/−*^ mouse retinas compared with wild-type retinas (Fig. 7E). These observations suggest an altered fatty acid synthesis and β-oxidation in mouse retinas lacking IGF-1R.

## Discussion

Earlier studies showed that IGF-1 receptors are expressed in the retina and outer and inner segments of the photoreceptors [31, 32]. Our data show that rod-specific deletion of IGF-1R resulted in rod degeneration, and the function gradually decreased with age. This functional loss was exacerbated in mice lacking IGF-1R in other retinal cells, including rod photoreceptor cells. Our studies also suggest that IGF-1R is indispensable for photoreceptor neuroprotection.

It is not known how the retinal IGF-1R is activated by which tissue-/cell-specific production of IGF-1. The liver is the main site for the production of IGF-1 [29]. Our study showed that loss of circulating IGF-1 does not affect the retinal function, suggesting that IGF-1 could be made in the retina. IGF-1 expression has also been observed in Müller glia [30]. We found that loss of IGF-1 in Müller cells does not affect retinal function. The inner photoreceptor matrix (IPM) situated between RPE and photoreceptor cells has previously been shown to have IGF-1 immunoreactivity [36] and also contains high levels of IGF-1 binding protein (IGFBP), which binds IGF-1 and regulates its availability [36]. Cultured human RPE cells have been shown to synthesize and release IGF-1, suggesting that the RPE may serve as a source of IPM-IGF-1 in vivo [36]. The presence of IGF-1 and IGFBP in the IPM, together with the presence of IGF-1 receptors on both photoreceptor and RPE cells [36], suggests the presence of an outer retina autocrine-paracrine system. Consistent with these earlier studies, in the present study, we found increased IGF-1, IGFBP3 protein expression in the RPE, and increased IGF-1R expression in the photoreceptor cells. Our finding on the higher expression of IGF-1R in photoreceptor cells is consistent with a previous study that showed IGF-1R expression is more than 100-fold higher than insulin receptors in photoreceptor cells [37]. Collectively, these findings suggest that RPE may provide the IGF-1 for photoreceptor IGF-1R activation.

IGF1 signaling is known to activate the PI3K pathway [8, 38]. Our studies on ^*rod*^*Igf-1r*^*−/−*^ mice showed decreased levels of *myo*-inositol, which is an important molecule in PI synthesis [34]. We observed significantly lower levels of PI(3)P, PI(3,4)P_2_, and PI(3,4,5)P_3_, suggesting IGF-1R regulates the PI-3 kinases. Interestingly, the reduced levels of PI(4)P and PI(4,5)P_2_ in both ^*rod*^*Igf-1r*^*−/−*^ and ^*ret*^*Igf-1r*^*−/−*^ mice suggest that IGF-1R may regulate phosphatidylinositol 4-kinase (PI4K generates PI(4)P from PI), phosphatidylinositol 5-phosphate 4-kinase (PIP4K generates PI4,5P_2_ from PI(4)P), and phosphatidylinositol 4-phosphate 5-kinase (PIP5K generates PI (4,5)P_2_ from PI(5)P).

The ratios of NADH/NAD and NADPH/NADP are significantly altered in ^*rod*^*Igf-1r*^*−/−*^ mice, suggesting an altered cellular redox and antioxidative imbalance in these mice. Existing evidence suggests that pyridine nucleotides NAD (includes NAD ^+^ and NADH) and NADP (includes NADP^+^ and NADPH) are common biological mediators of several cellular processes, including regulation of cellular redox, antioxidant metabolism, generation of oxidative stress, energy metabolism, gene expression, cell death, mitochondrial function, aging, and calcium homeostasis [39]. NAD regulates mitochondrial function and energy metabolism, whereas NADPH regulates cellular antioxidant metabolism [39]. IGF1 has previously been shown to regulate the tumor form of pyruvate kinase M2, decrease pyruvate kinase activity, and increase AMP/ATP ratio [40]. The retinal cells and rod photoreceptors lacking IGF-1R exhibited increased pyruvate kinase activity and increased ATP production. These studies suggest that IGF-1R activation promotes cellular anabolism. The steady-state metabolite profile showed increased pyruvate levels along with an increase of several metabolites in ^*rod*^*Igf-1r*^*−/−*^ mice. We also observed significant alterations in alanine, aspartate, and glutamate metabolism; pyruvate metabolism; glycine, serine, and threonine metabolism; and a minor impact on inositol phosphate metabolism. It is interesting to note that the interaction of all of these metabolites is centered on NADH and identified potential changes in oxygen, ATP/ADP, and acetoacetyl CoA. The decreased NADH levels in the ^*rod*^*Igf-1r*^*−/−*^ mice resulted in the elevation of ATP and several other metabolites identified in the present study.

IGF-1 has been shown to stimulate de novo fatty acid biosynthesis in Schwann cells during myelination [41]. High-fat-diet-induced insulin resistance and glucose intolerance have been observed in gender-specific IGF-1R heterozygous mice [42]. In ^*rod*^*Igf-1r*^*−/−*^ mice, we found a significant decrease in the expression of fatty acid synthesis genes and decreased levels of some fatty acids. Photoreceptor cells are highly metabolic and glycolytic [43]. Studies also showed that the retina uses fatty acid β-oxidation for energy [44]. A shortage of lipids and glucose in the retina has been shown to promote neovascular age-related macular degeneration [45]. The ^*rod*^*Igf-1r*^*−/−*^ mouse retinas show significantly reduced β-oxidation. The steady-state metabolite analysis showed increased levels of β-hydroxybutyrate (β-HB) in ^*rod*^*Igf-1r*^*−/−*^ mice. The RPE metabolizes fatty acids to produce β-HB, which is transported to the retina and metabolized into various TCA cycle intermediates [44].

In summary, IGF-1R is essential for photoreceptors and retinal neuroprotection. Our findings suggest that IGF-1R maintains photoreceptor structure and function and regulates energy, lipid, and phosphoinositide metabolism. IGF-1 levels have been shown to decrease in the aging brain [46]. The role of IGF-1 in age-related retinal diseases is unknown. Activation of IGF-1R in retinal disease might prevent the loss of photoreceptors in aging.

## Materials and methods

### Antibodies

Polyclonal IGF-1R antibody, mouse monoclonal Cre antibody, and mouse glutamine synthetase antibodies suitable for immunohistochemistry were purchased from Abcam (Cambridge, MA). Rabbit polyclonal IGF-1R antibody was purchased from Protientech (Rosemount, IL). Rabbit polyclonal red/green cone opsin (M-opsin), cone arrestin, actin, and rabbit and mouse secondary antibodies were obtained from Millipore (Billerica, MA). Monoclonal 1D4 rhodopsin antibody was a kind gift from Dr. James F. McGinnis (University of Oklahoma Health Sciences Center). DAPI used for nuclear staining was procured from Invitrogen-Molecular Probes (Carlsbad, CA). The monoclonal anti-arrestin antibody was a kind gift from Dr. Paul Hargrave (University of Florida, Gainesville). Polyclonal glial fibrillary acidic protein (GFAP) was purchased from Dako (Carpinteria, CA).

### Animals

Our study followed the NIH Guide for the Care and Use of Laboratory Animals and the ARVO Statement for the Use of Animals in Ophthalmic and Vision Research. The Institutional Animal Care and Use Committee at the University of Oklahoma Health Sciences Center approved all protocols. The floxed IGF-1R (Stock No: 012251), floxed IGF-1 (Stock No: 016831), Chx10-EGFP/Cre (Stock No: 005105), transgenic glial high-affinity glutamate transporter (GLAST-Cre^ER^) (Stock No: 012586), and albumin-Cre (Stock No: 003574) were purchased from the Jackson Laboratory (Bar Harbor, ME). The generation and efficiency of rhodopsin-Cre (i75Cre) have been described earlier [26]. All mice were screened for *rd1* and *rd8* mutations and were negative for these mutations. The eyes or retinas were harvested after CO_2_ asphyxiation. For metabolic experiments, mouse retinas were harvested under deep anesthesia or retinas removed after decapitation. These tissues were subjected to biochemistry or immunohistochemistry. To eliminate bias, mice of the same sex, age, and genetic strain were randomly assigned to each experimental group. Litters were mixed to prevent litter bias. Once mice were genotyped and provided with unique eartag identifiers, cohorts were selected randomly by the principal investigator (Dr. Rajala), such that research personnel doing experiments were blinded and only knew the eartag number.

### Immunohistochemistry and immunoblot analyses of retinas

Immunohistochemistry and immunoblot analysis were done as previously described [13]. In the current study, blots were incubated with IGF-1R (1:1000), cone arrestin (1:1000), transducin α (1:1000), rhodopsin (1:10,000), rod arrestin (1:1000), M-opsin (1:1000), and actin (1:1000) antibodies (see Table S3) overnight at 4 °C. The blots were then washed and incubated with HRP-coupled anti-mouse or anti-rabbit secondary antibodies (as appropriate) for 60 min at room temperature. After washing, blots were developed with enhanced SuperSignal™ West Dura Extended Duration Substrate (Thermo Fisher Scientific, Waltham, MA) and visualized using a Kodak Imager with chemiluminescence capability.

### Isolation of polyribosomes containing actively translating mRNAs

Using a modified method from Cleuren et al. [47], we isolated polyribosomes containing actively translating mRNAs. Retinas from two mice (2-to-4 months old) were removed and placed in a DMEM medium containing cycloheximide (100 µg/mL) and incubated for 10 min. Then, the retinas were flash-frozen in liquid nitrogen and pulverized with a hand homogenizer. The powder was resuspended in 200 µl of polysome buffer (50 mM Tris-HCl [pH 7.5], 100 mM KCl, 12 mM MgCl_2_, 1% Igepal CA-630, 1 mM dithiothreitol, 200 U/mL RnaseOUT, 1 mg/mL heparin sodium salt, 100 µg/mL cycloheximide plus EDTA-free protease inhibitor cocktail in DEPC water), mixed by pipetting, and centrifuged at 15,000 RPM at 4 °C. The clear lysate was incubated with a purified rabbit monoclonal HA antibody (5 µl/200 µl lysate) for 1 h at 4 °C. Magnetic protein G beads, equilibrated in polysome buffer, were added to the retina lysate containing HA antibody. Beads were then incubated for an additional 30 min at 4 °C. The magnetic beads containing immune-complexes were washed three times with high salt buffer (50 mM Tris-HCl [pH 7.5], 300 mM KCl, 12 mM MgCl_2_, 1% Igepal CA-630, 1 mM dithiothreitol, 200 U/mL RnaseOUT, 1 mg/mL heparin sodium salt, 100 µg/mL cycloheximide plus EDTA-free protease inhibitor cocktail). To the beads, we added 500 µl of TRIzol and isolated RNA using a PureLink RNA Mini Kit (Ambion, Carlsbad, CA). First-strand cDNA was synthesized using Superscript III first-strand synthesis kit (Invitrogen).

### Determination of myo-inositol and phosphoinositides from the retina

The *myo*-inositol concentration was determined as described previously [48]. The phosphoinositide extraction and the levels of PI(3)P, PI(4)P, PI(3,4)P_2_, PI(4,5)P_2,_ and PI(3,4,5)P_3_ were determined as described previously [49]. Lipid phosphorous was determined as described and we normalized the phosphoinositide levels to phospholipid [49].

### Lipid analysis

Fatty acid profiles were determined for the retina. Total lipids were extracted following the method of Bligh and Dyer [50], with modifications [51]. To each lipid extract were added 15:0 and 17:0 as internal standards. The lipid extracts were subjected to acid hydrolysis/methanolysis to generate fatty acid methyl esters (FAMEs) [52]. FAMEs were quantified using an Agilent Technologies 6890N gas chromatograph with a flame ionization detector (GC) [53]. The results were also confirmed by GC-MS.

### Metabolic profiling

Metabolic profiling was performed based on a previously published method [54, 55] with specific (retina) modifications. Briefly, 4–8 mg of frozen retina tissue was pulverized using a tissue grinder Qiagen TissueLyser II containing pre-chilled metal beads, followed by methanol:chloroform: water (1:1:1) extraction. Ribitol, as an internal standard, was added to each sample at the first step of extraction. After 10 min incubation at 4 °C, samples were centrifuged at 20,000 rpm for 5 min, and 600 μL of supernatant was transferred to the new 2-mL tubes and completely dried in a vacuum concentrator for 4 h. Dried residues were derivatized in two steps. First, samples were dissolved in 25 μL of 20 mg/mL methoxyamine hydrochloride in pyridine for 2 h at 37 °C with constant orbital shaking. Second, 35 μL N,O-bis(trimethylsilyl)trifluoroacetamide was added, and the samples were mixed at 37 °C for 30 min. A mixture of alkanes (C10–C24) was used as a retention time standard. After derivatization, the samples were transferred into glass vials and 1 μL was injected into the GC–MS system (Agilent 7890B-5977A) in splitless mode. Each sample was analyzed in duplicate. The full scan from 60 to 600 *m*/*z* was performed. In addition to the tissue samples, three QCs were prepared by pooling an equal amount of each experimental sample, and analytical standards of pyruvate, lactate, glucose, fructose-6-phosphate, hydroxybutyrate, leucine, and isoleucine were prepared and analyzed. All chemicals were purchased from Sigma Aldrich (St. Louis, MO, USA), except pyridine and methoxyamine hydrochloride (Thermo Fisher Scientific, Waltham, MA, USA). Metabolites were annotated using the MassHunter software (Agilent) according to the National Institute of Standards and Technology (NIST, Gaithersburg, USA) library and external standards listed above. The relative abundance of metabolites was calculated by peak area normalized by exact sample weight and internal standard. PCA was performed using an online application (https://scienceinside.shinyapps.io/mvda/) based on R-project (https://scholar.google.com/scholar_lookup?title=R%3A%20A%20Language%20and%20Environment%20for%20Statistical%20Computing&publication_year=2017&author=Team%20RC) on Log-transformed data. Pathway analysis was performed using the MetaboAnalyst, v. 4 online tool (https://currentprotocols.onlinelibrary.wiley.com/doi/10.1002/cpbi.86).

### Real-time qRT-PCR

Messenger RNA (mRNA) levels of ACC, PGC-1α, PGC-1β, C/EBPβ, FAS, SREBP-1c, rhodopsin, GFAP, RPE65, IGF-1, IGFBP3, and IGF-1R were analyzed by quantitative real-time RT-PCR (qPCR) using specific primer pairs (Table 1) using Primer3 software. All primer sets were designed from mRNA sequences spanning big introns to avoid amplification from possible genomic DNA contamination. The primer sequences were checked by a BLAST search to assure sequence specificity. RNA (TRIzol and Pure link RNA kit; Invitrogen) was isolated from two mice (pooled four retinas). The first-strand cDNA was synthesized using Superscript III first-strand synthesis kit (Invitrogen). The RT products were diluted 1:3, and 2 µl of each of the diluted RT products and 3 pmol of primers and Eva green supermix (Bio-Rad) were used for a final volume of 12 µl. The PCR was carried out on a CFX96TM Real-Time System and C1000 Touch Thermal Cycler (Bio-Rad). Fluorescence changes were monitored after each cycle (SYBR Green). Melting curve analysis was performed (0.5 °C/s increase from 55 to 95 °C with continuous fluorescence readings) at the end of 40 cycles to ensure that specific PCR products were obtained. Amplicon size and reaction specificity were confirmed by electrophoresis on a 2.0% agarose gel. All reactions were performed in triplicate. The average CT (threshold cycle) of fluorescence units was used for analysis. Each mRNA level was normalized by either the 18 S rRNA or by actin. Quantification was calculated using the CT of the target signal relative to the 18 S rRNA or actin signal in the same RNA sample. Effects were quantified and expressed by the *x*-fold change method calculated as: mean CQ (quantification cycle) gene − mean CQ housekeeping gene − dCQ and –fold = 2∧ − dCQ.

**Table 1:** Real-Time PCR primers for IGF-1 signaling and fatty acid synthesis genes.

Gene	Forward primer	Reverse primer
Igf1	AAAGCAGCCCCGCTCTATCC	CTTCTGAGTCTTGGGCATGTCA
Igfbp3	AAGCACCTACCTCCCCTCCCAA	TGCTGGGGACAACCTGGCTTTC
Igf1r	GCTTCTGTGAACCCCGAGTATTT	TGGTGATCTTCTCTCGAGCTACCT
Rhodopsin	CAAGAATCCACTGGGAGATGA	GTGTGTGGGGACAGGAGAACT
Pde6α	TCCTTGGGAGCAGCTAAAGG	CCTTCCCCCGGTAGTGAAAG
RPE65	GTTCCCCTGCAGTGATCGTT	GCAACATGAAGCCAAACCCC
GFAP	CAGCCTCAGGTTGGTTTCAT	CTCTCCTGTGCTGGCTACTGT
ACC	GGACAGACTGATCGCAGAGAAAG	GCTGTTCCTCAGGCTCACAT
PGC-1α	AAGCACTTCGGTCATCCCTG	TGAGTCTCGACACGGAGAGT
PGC-1β	GACTTGCCAGAGCTTGACCT	GAAGAGCTCGGAGTCATGGG
C/EBPβ	GCAAGAGCCGCGACAAG	GGCTCGGGCAGCTGCTT
FAS	GCTGCGGAAACTTCAGGAAAT	AGAGACGTGTCACTCCTGGACTT
SEEBP-1c	CTGGATTTGGCCCGGGGAGATTC	TGGAGCAGGTGGCGATGAGGTTC
18S RNA	TTTGTTGGTTTTCGGAACTGA	CGTTTATGGTCGGAACTACGA
β-actin	ACTGGGACGACATGGAGAAG	GGGGTGTTGAAGGTCTCAAA

### Pyruvate kinase enzyme assay

The lactate dehydrogenase (LDH) coupled enzyme assay was used to measure pyruvate kinase (PK) enzyme activity [56]. The assay was carried out in the presence of mouse retinal lysate containing an enzyme buffer mixture (50 mM Tris-HCl [pH 7.4], 100 mM KCl, 5 mM MgCl_2_, 1 mM ADP, 0.5 mM PEP, and 0.2 mM NADH [reduced form of NAD^+^]) and 8 U of LDH with a reaction volume of 1.0 ml. The PK activity was measured spectrophotometrically by monitoring the reduction in the absorbance at 340 nm from the oxidation of NADH.

### Statistical analysis

We estimated the sample size by power analysis [57]. Data were subjected to appropriate statistical evaluation to determine significant changes using GraphPad Prism 7.3 software. We did not exclude any sample from the statistical analysis. We used several statistical methods depending on the type of the experiments. Before determining the statistical analysis, we performed a series of normality tests on the data (Anderson-Darling test, D’Agostino & Pearson test, Shapiro-Wilk test, Kolmogorov-Smirnov test) to determine whether the data were normally distributed in a Gaussian manner or not normally distributed. If the data were not normally distributed, we performed the unpaired non-parametric test to compare the two groups. We ran multiple Man Whitney U tests on the data. To correct for multiple comparisons, we controlled the false discovery rate by setting *Q* = 1% and used the two-stage step of the method of Benjamini Krieger Yekutieli. For the normally distributed data, we performed a parametric test to compare two groups. We ran Welch’s correction to determine statistical significance. The resulting *p* values were used to deduce significance. We also used one-way ANOVA to determine whether there were any statistically significant differences between the means of three or more independent (unrelated) groups. We used a two-way ANOVA to estimate how the mean of a quantitative variable changes according to the levels of two categorical variables.

### Other methods

Electroretinography and optical coherence tomography were carried out as described [58]. Serum IGF-1 levels were measured using a kit from Abcam (ab108874) (Waltham, MA). Fatty acid β-oxidation (FAO) was measured using a kit from AssayGenie (Dublin, Ireland). The FAO activity assay is based on the oxidation of octanoyl-CoA, which is coupled to NADH-dependent reduction of INT to INT-formazan, and exhibits an absorption maximum at 492 nm [59]. NAD, NADH, NADP, and NADPH levels were measured as described [60]. ATP concentration was determined using an EnzyLight™ ATP Assay Kit from BioAssay Systems (Hayward, CA).

## Supplementary information

Uncropped images

Checklist

Author Contribution Statement

Supplementary Tables

Merged Figures File

## Data Availability

All data generated during or analyzed during this study are included in this published article and its Supplementary Information files.
