# Supplementary material for: Insulin-like growth factor 1 receptor mediates photoreceptor neuroprotection
Source: Cell Death Dis. 2022 Jul 15;13(7):613. doi: 10.1038/s41419-022-05074-3 (PMC9287313; doi:10.1038/s41419-022-05074-3)
Supplement: Supplementary file 1 — Uncropped images [file 41419_2022_5074_MOESM1_ESM.pptx]

## Slide 1
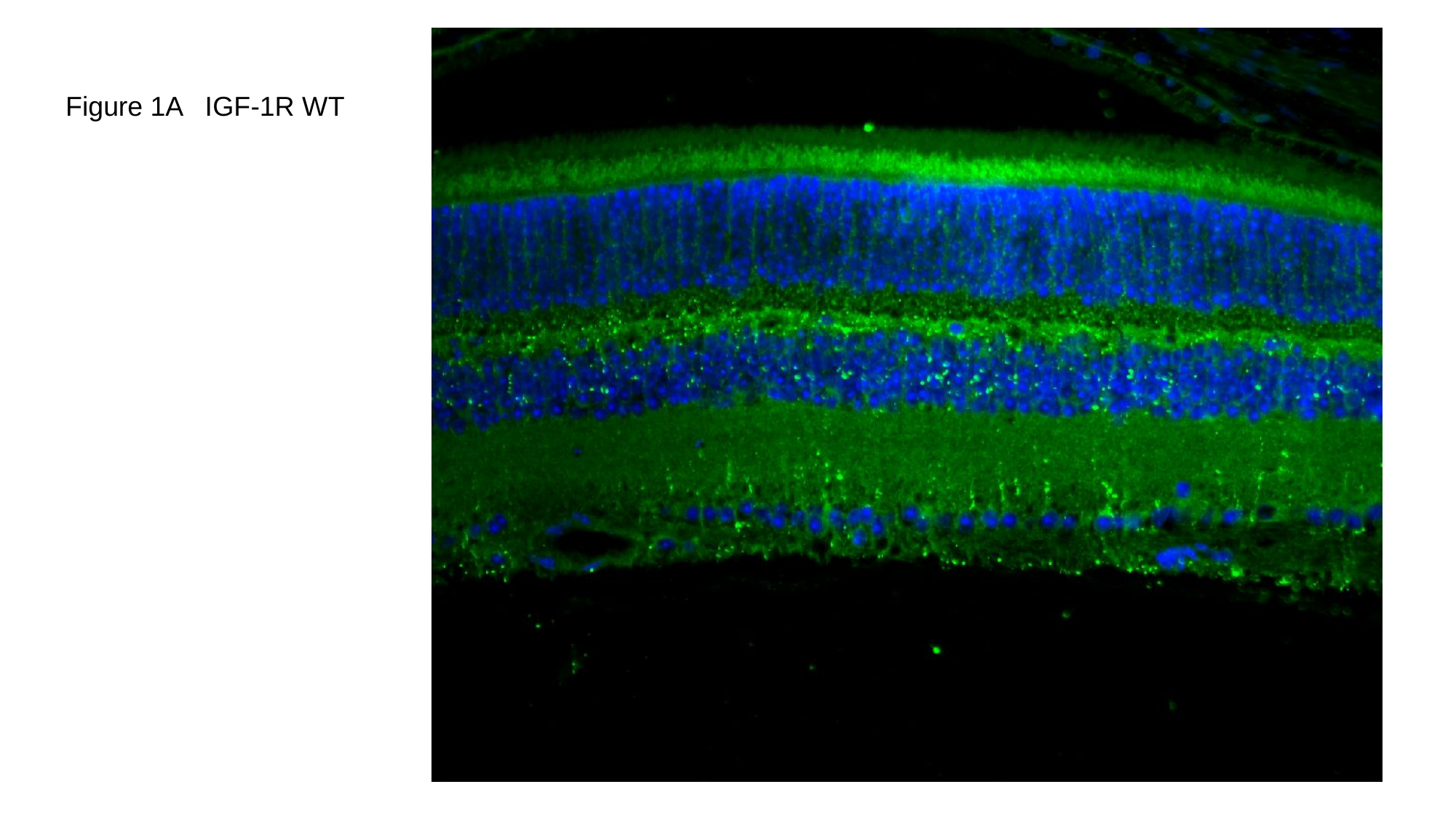

Figure 1A IGF-1R WT

## Slide 2
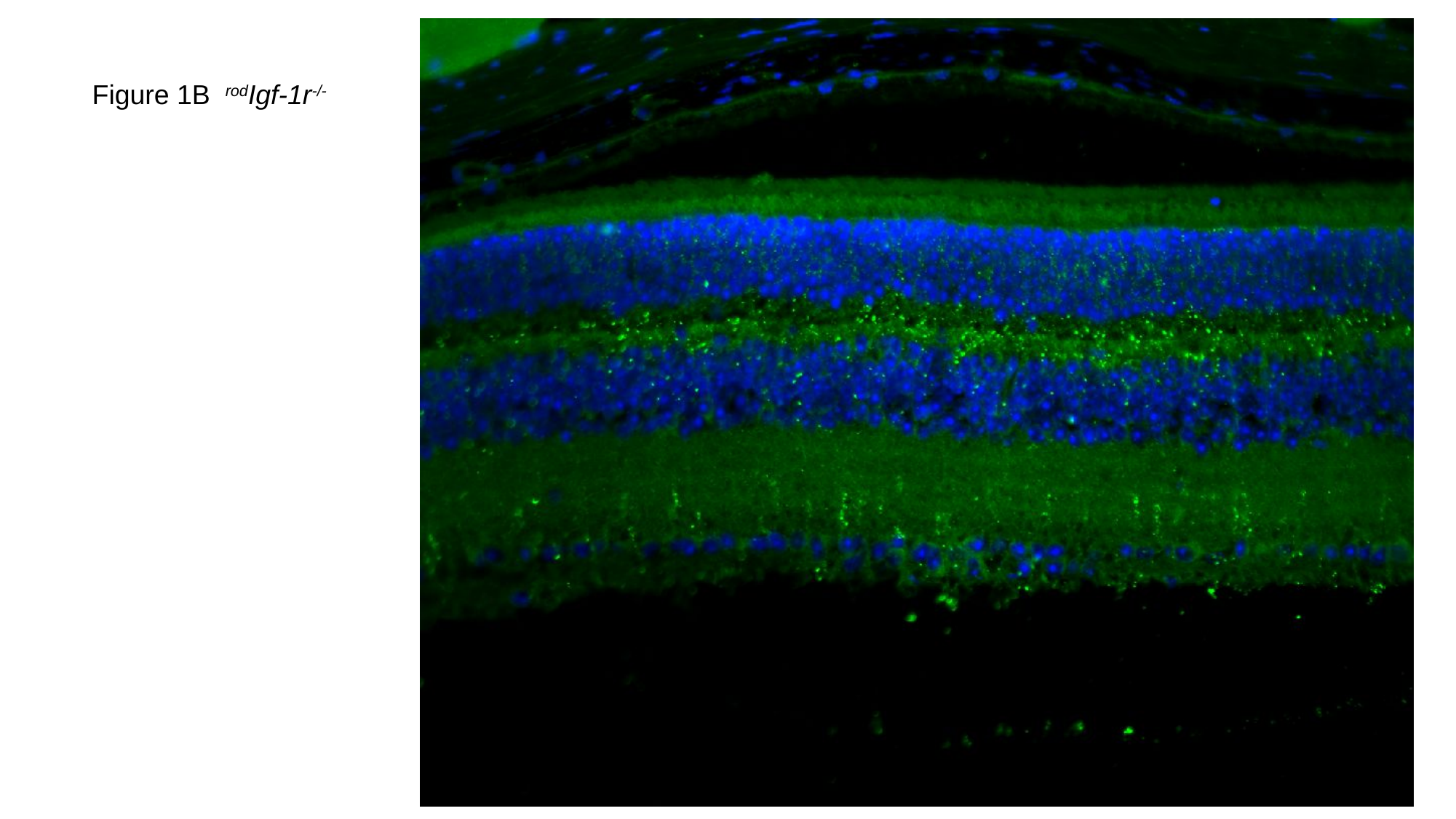

Figure 1B rodIgf-1r-/-

## Slide 3
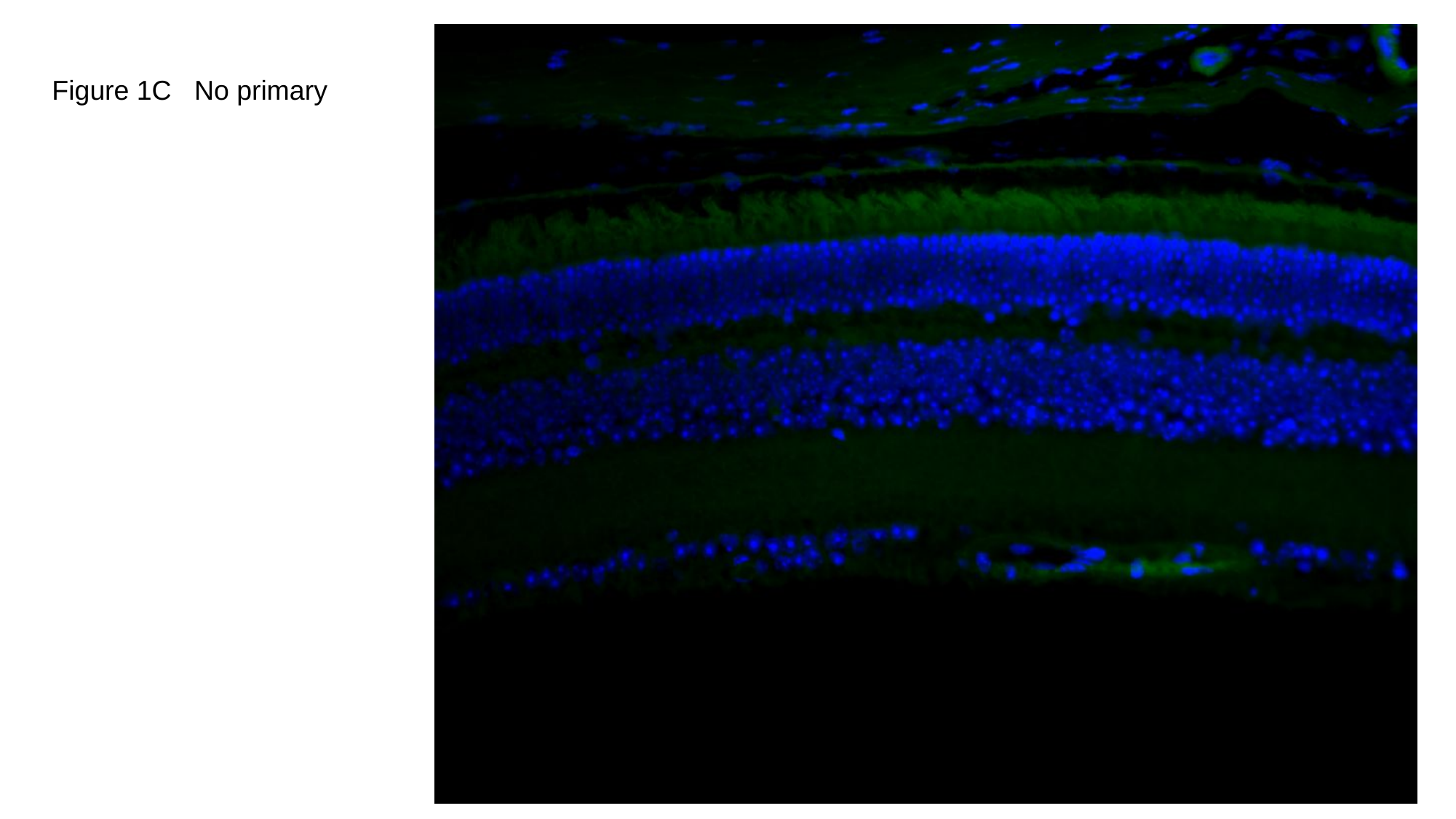

Figure 1C No primary

## Slide 4
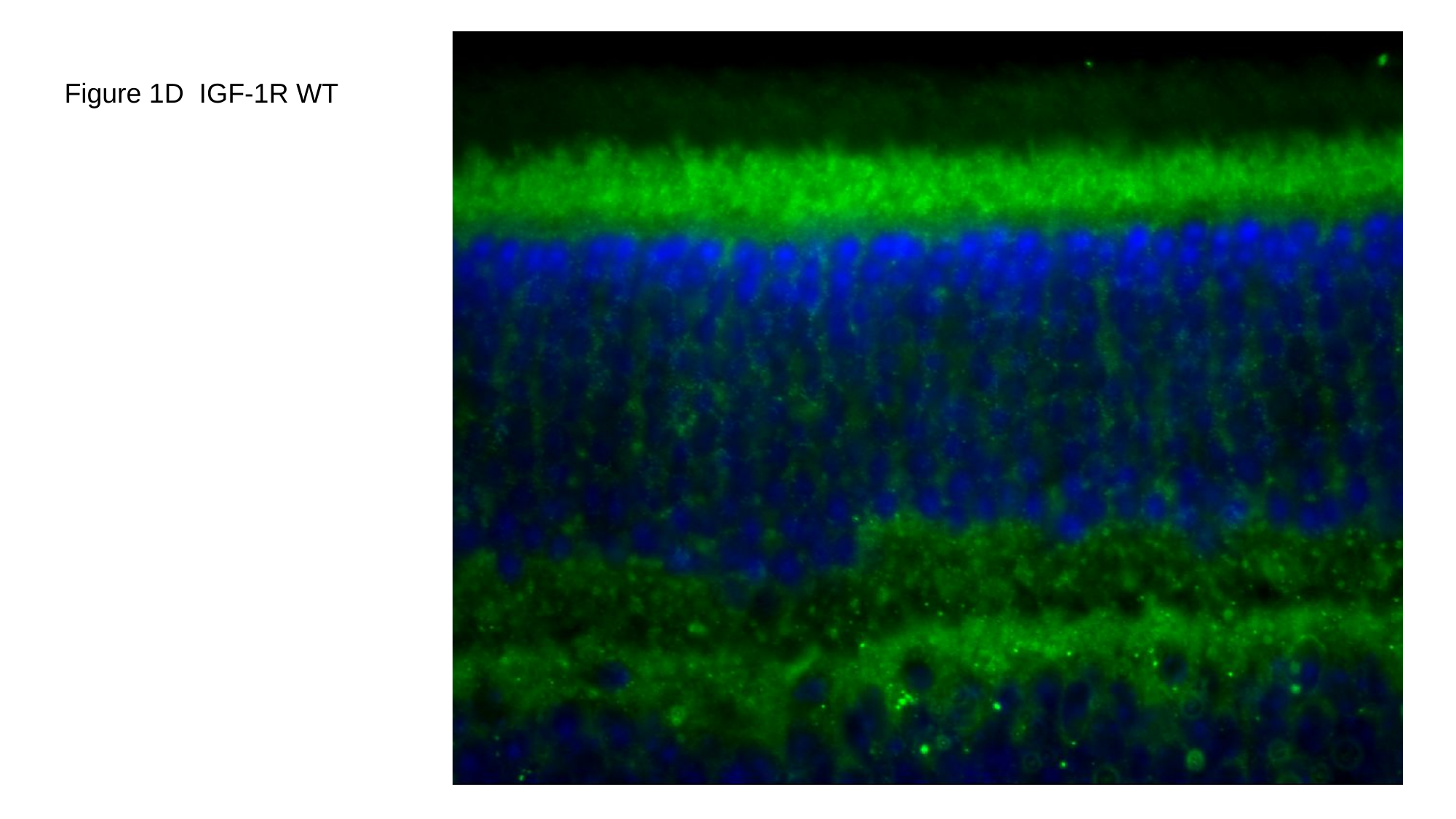

Figure 1D IGF-1R WT

## Slide 5
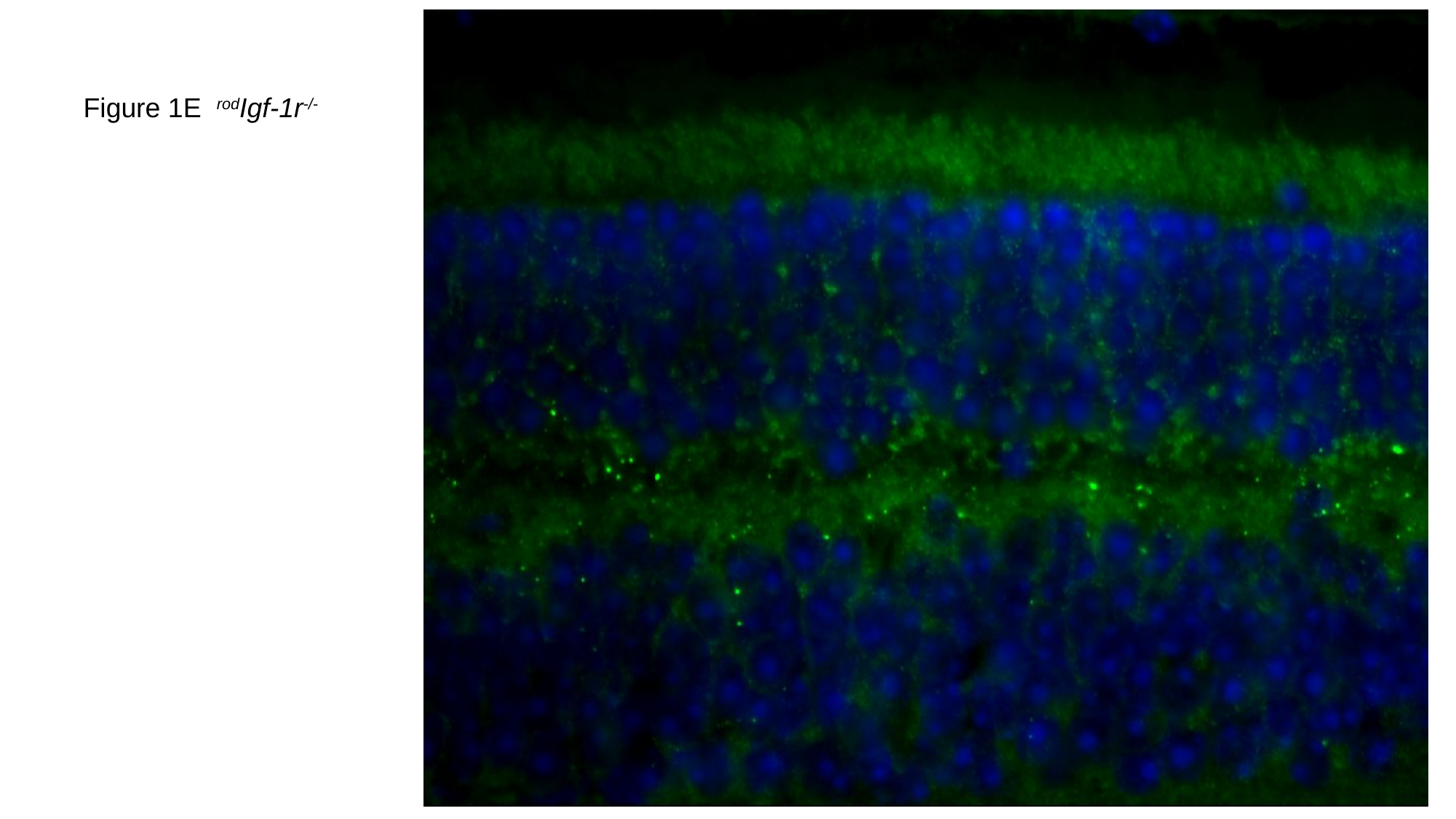

Figure 1E rodIgf-1r-/-

## Slide 6
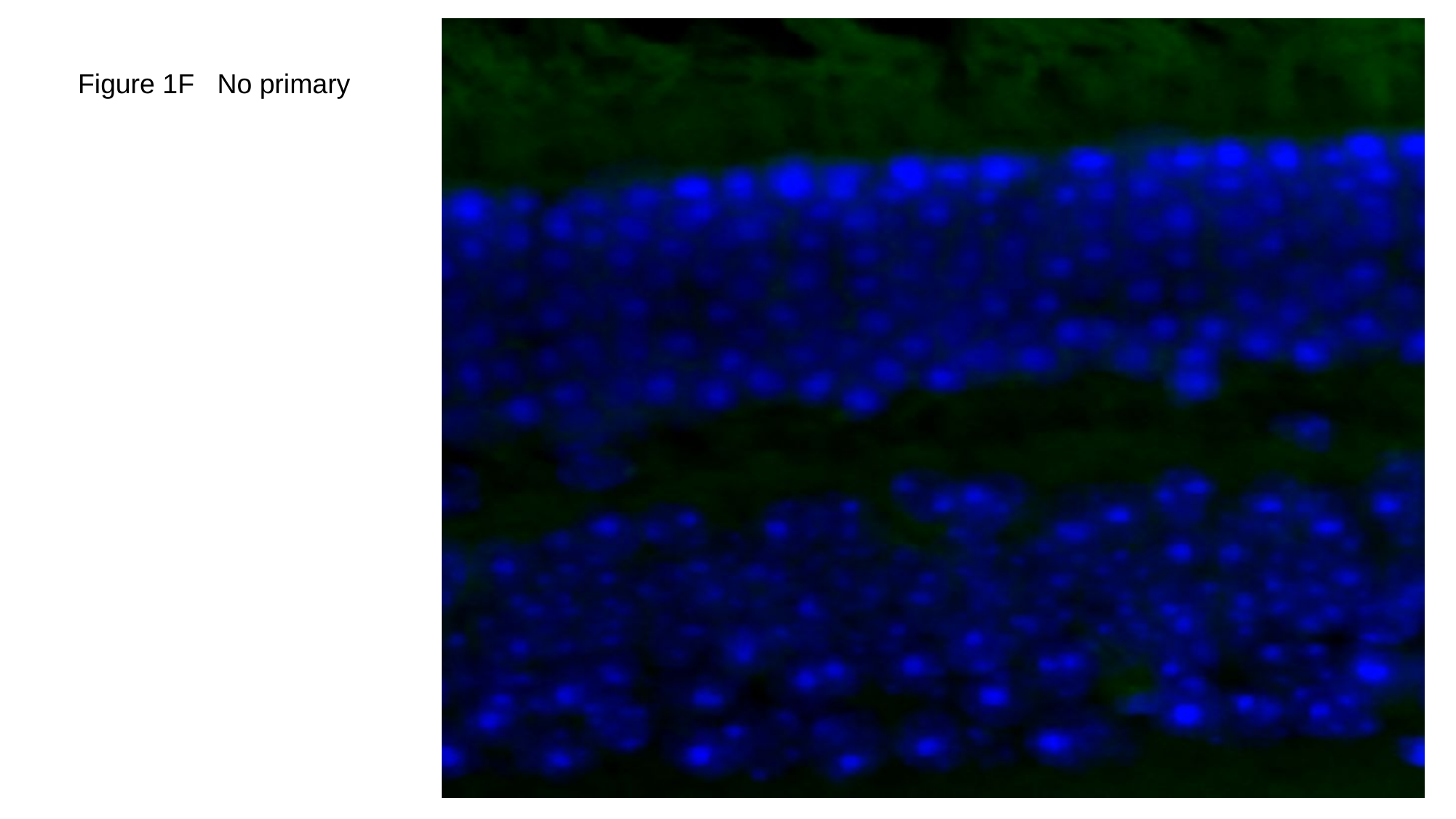

Figure 1F No primary

## Slide 7
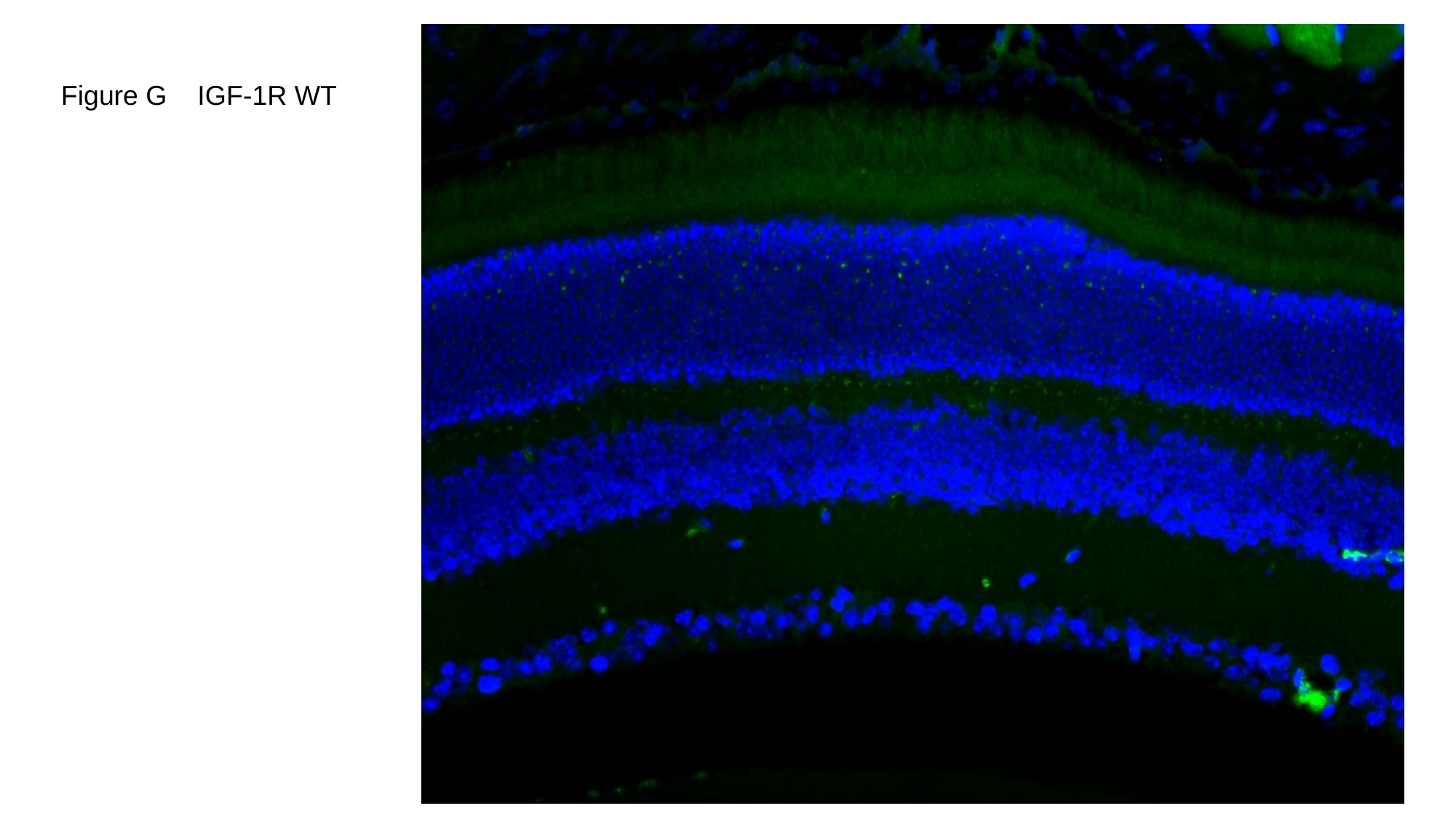

Figure G IGF-1R WT

## Slide 8
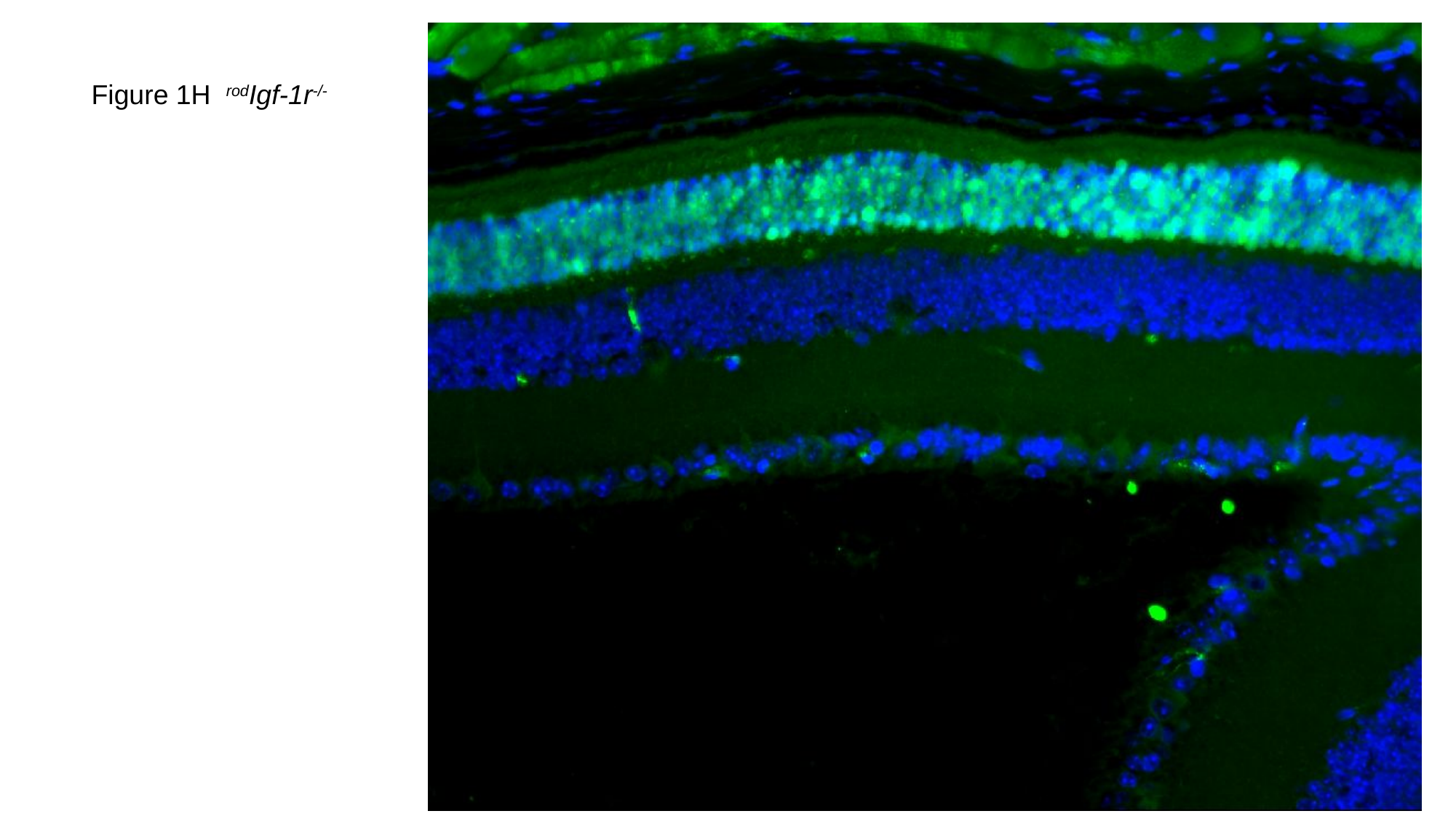

Figure 1H rodIgf-1r-/-

## Slide 9
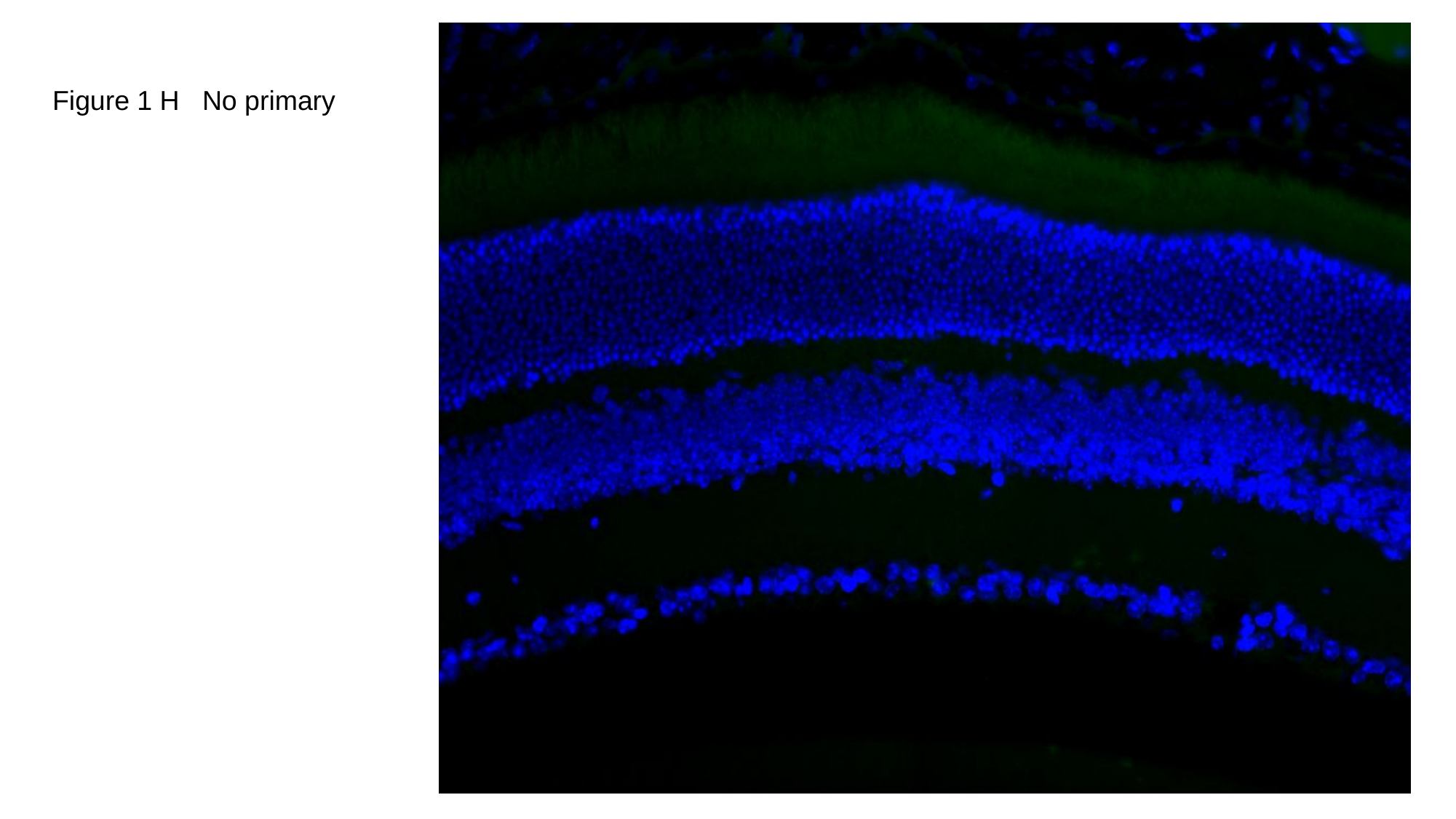

Figure 1 H No primary

## Slide 10
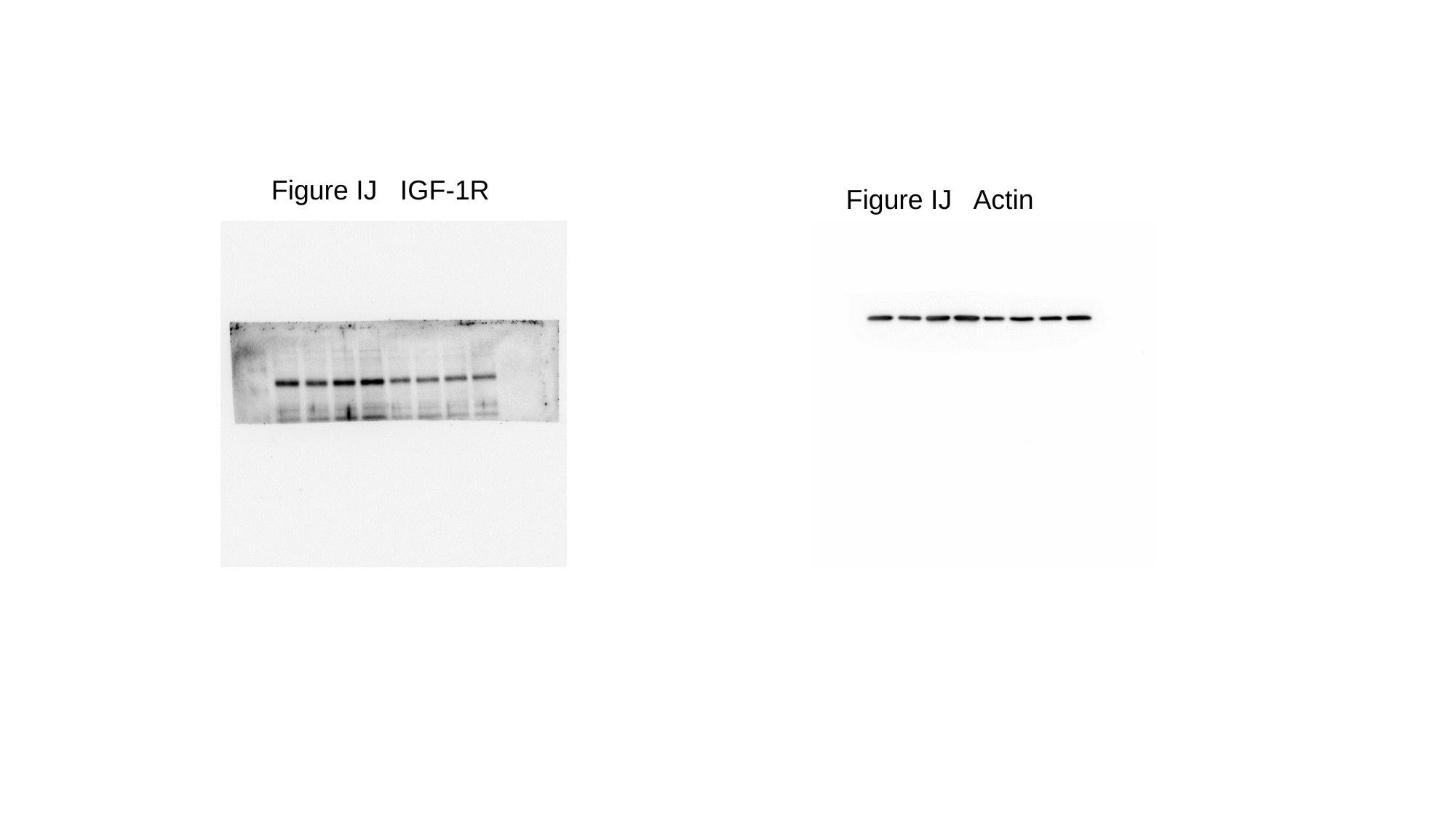

Figure IJ IGF-1R
Figure IJ Actin

## Slide 11
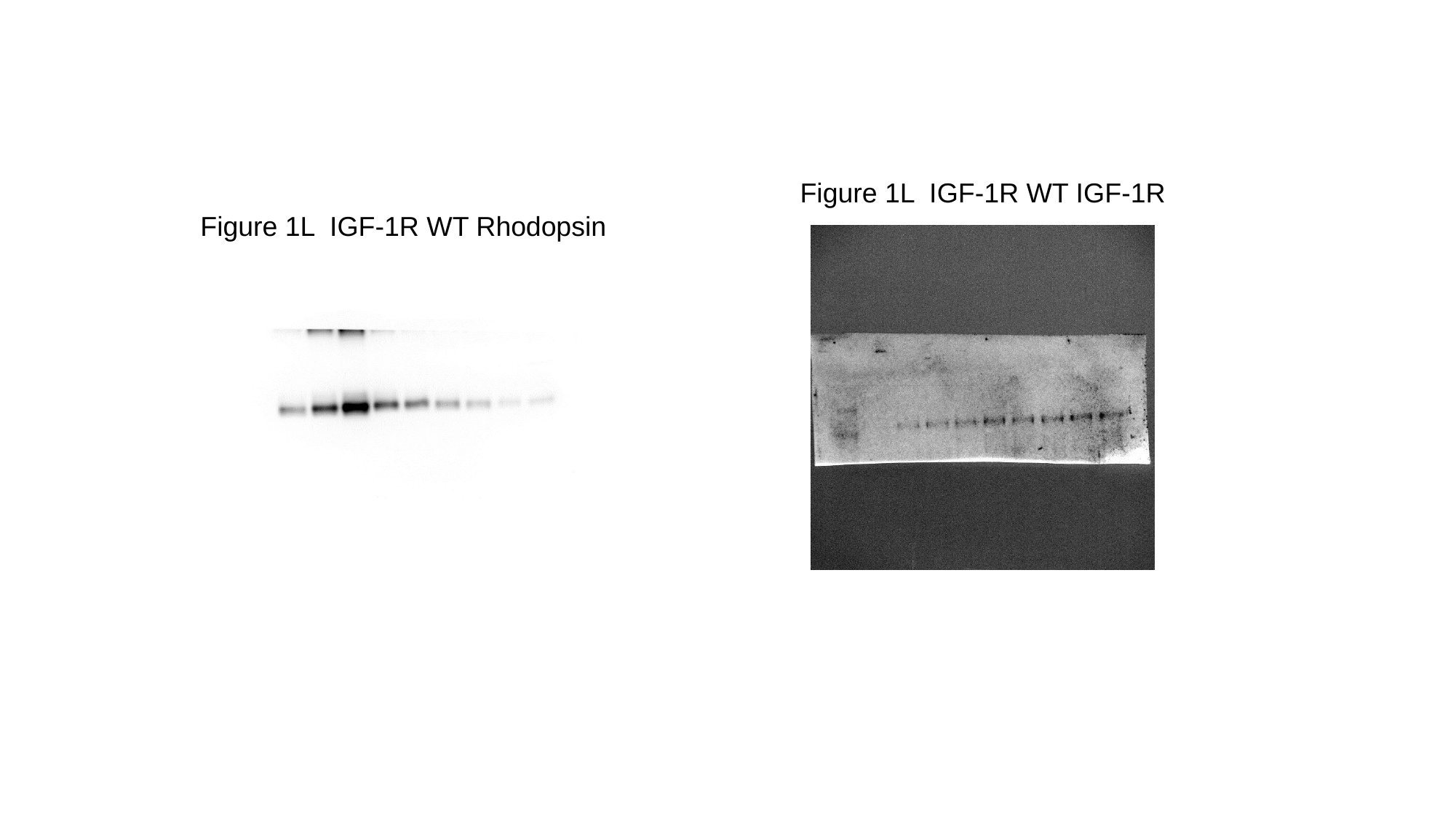

Figure 1L IGF-1R WT IGF-1R
Figure 1L IGF-1R WT Rhodopsin

## Slide 12
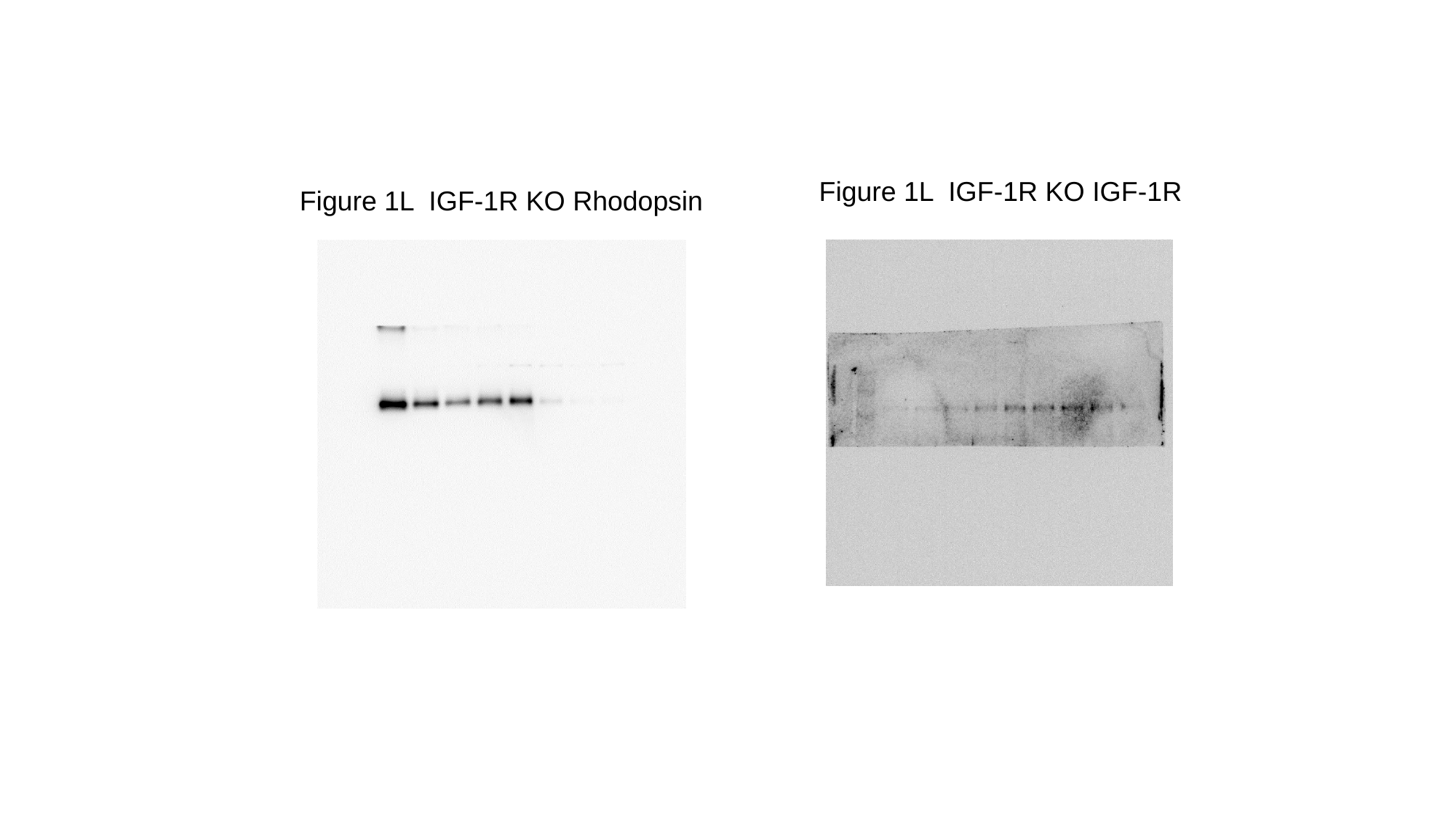

Figure 1L IGF-1R KO IGF-1R
Figure 1L IGF-1R KO Rhodopsin

## Slide 13
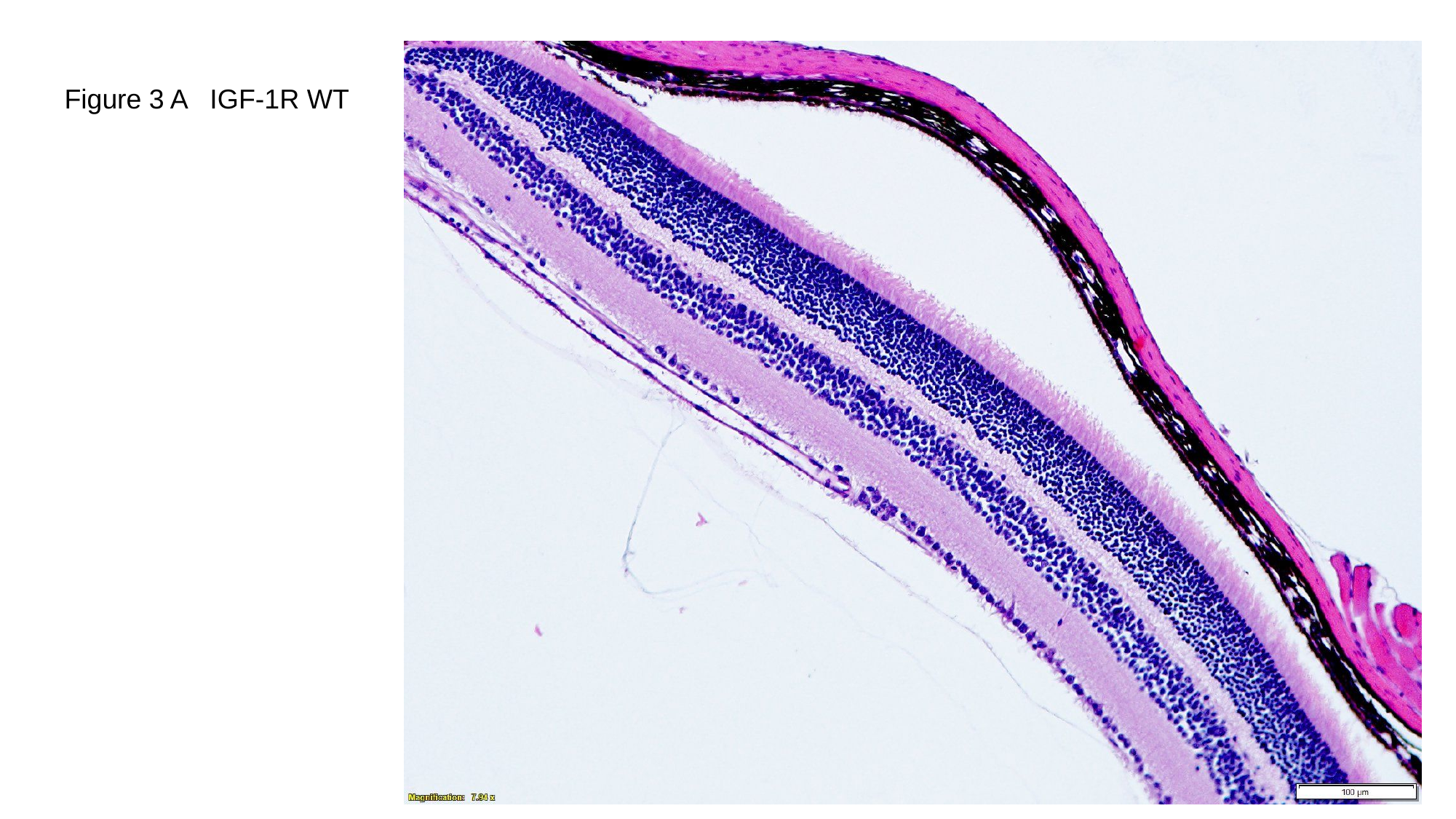

Figure 3 A IGF-1R WT

## Slide 14
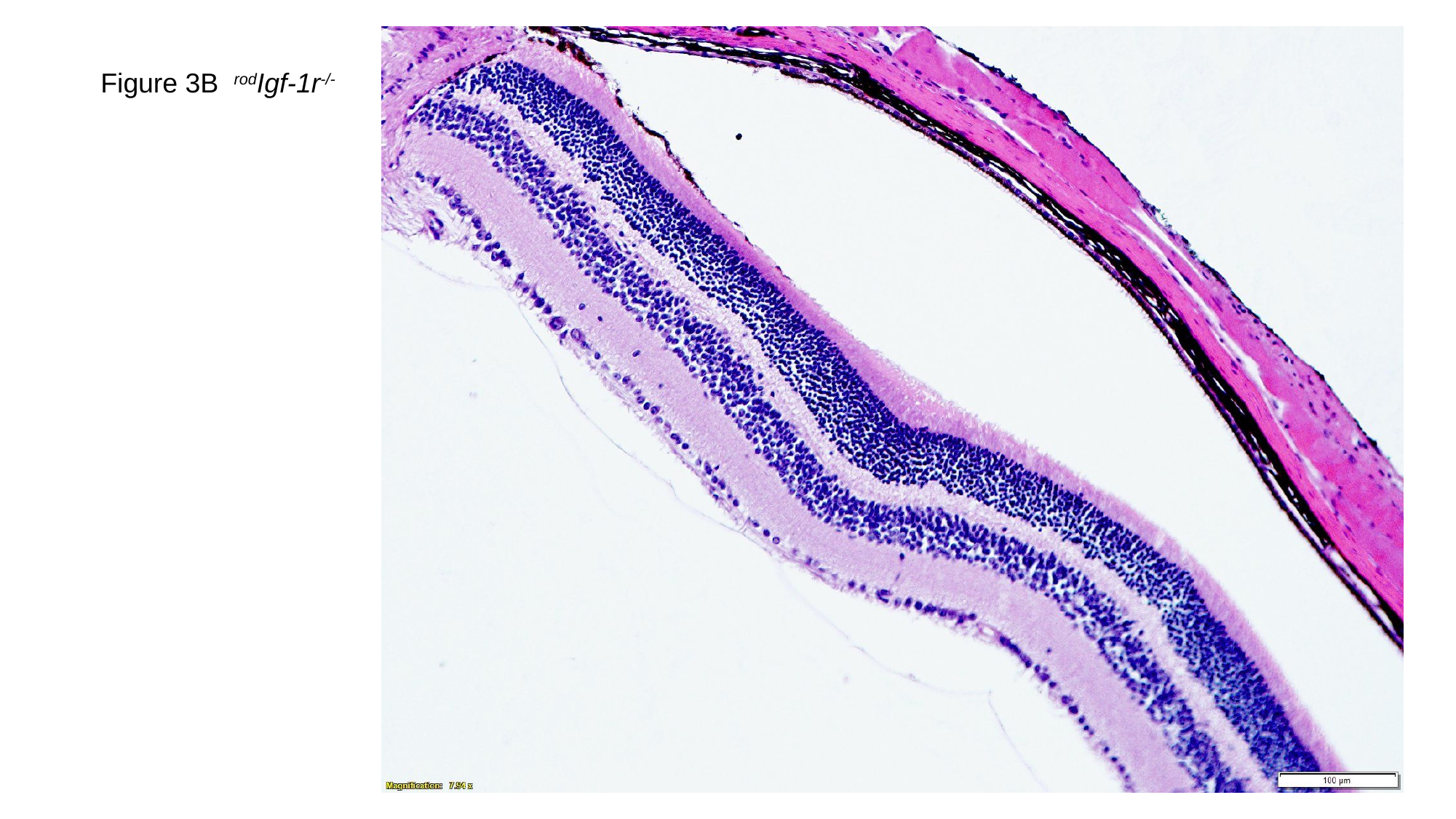

Figure 3B rodIgf-1r-/-

## Slide 15
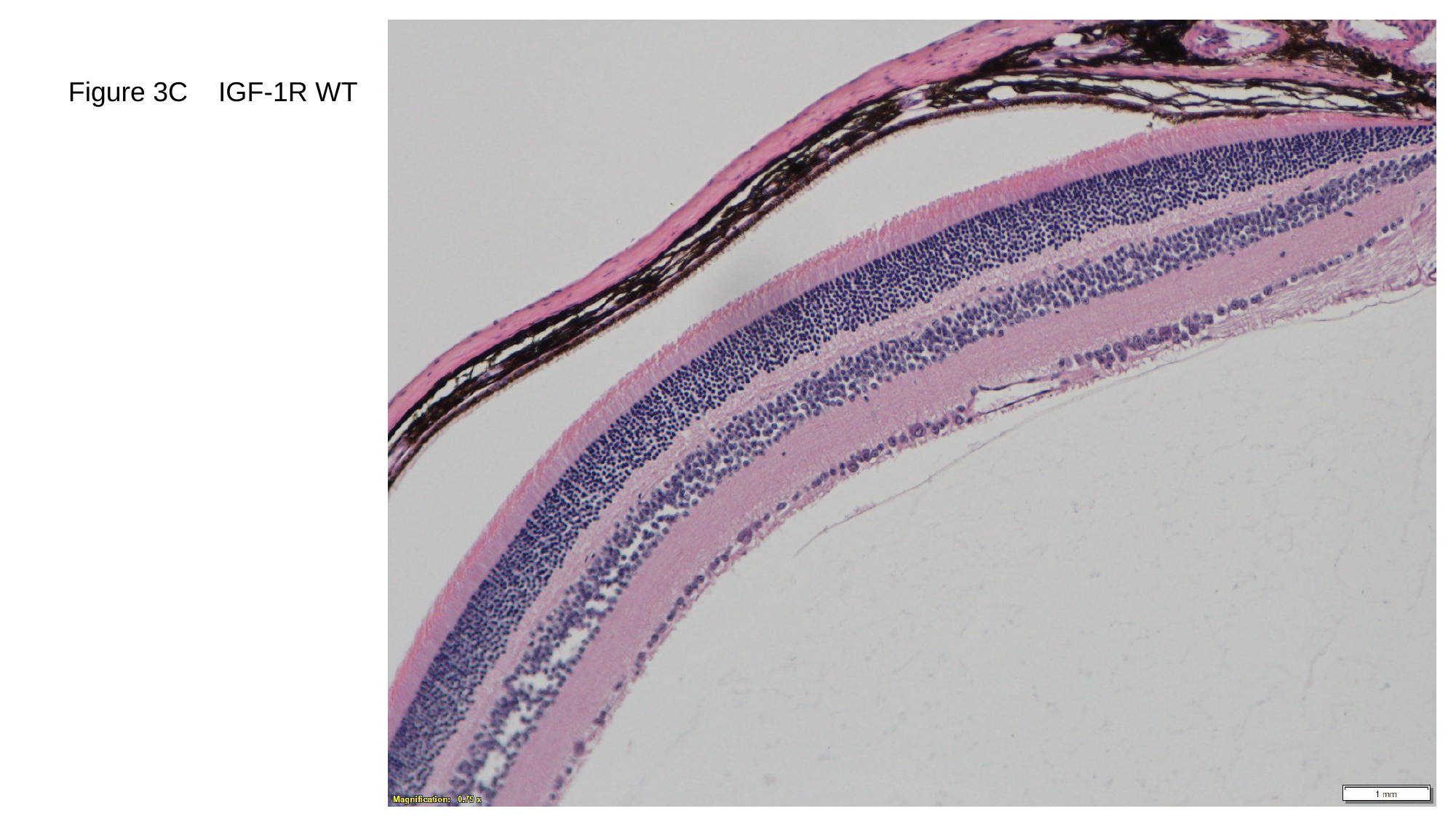

Figure 3C IGF-1R WT

## Slide 16
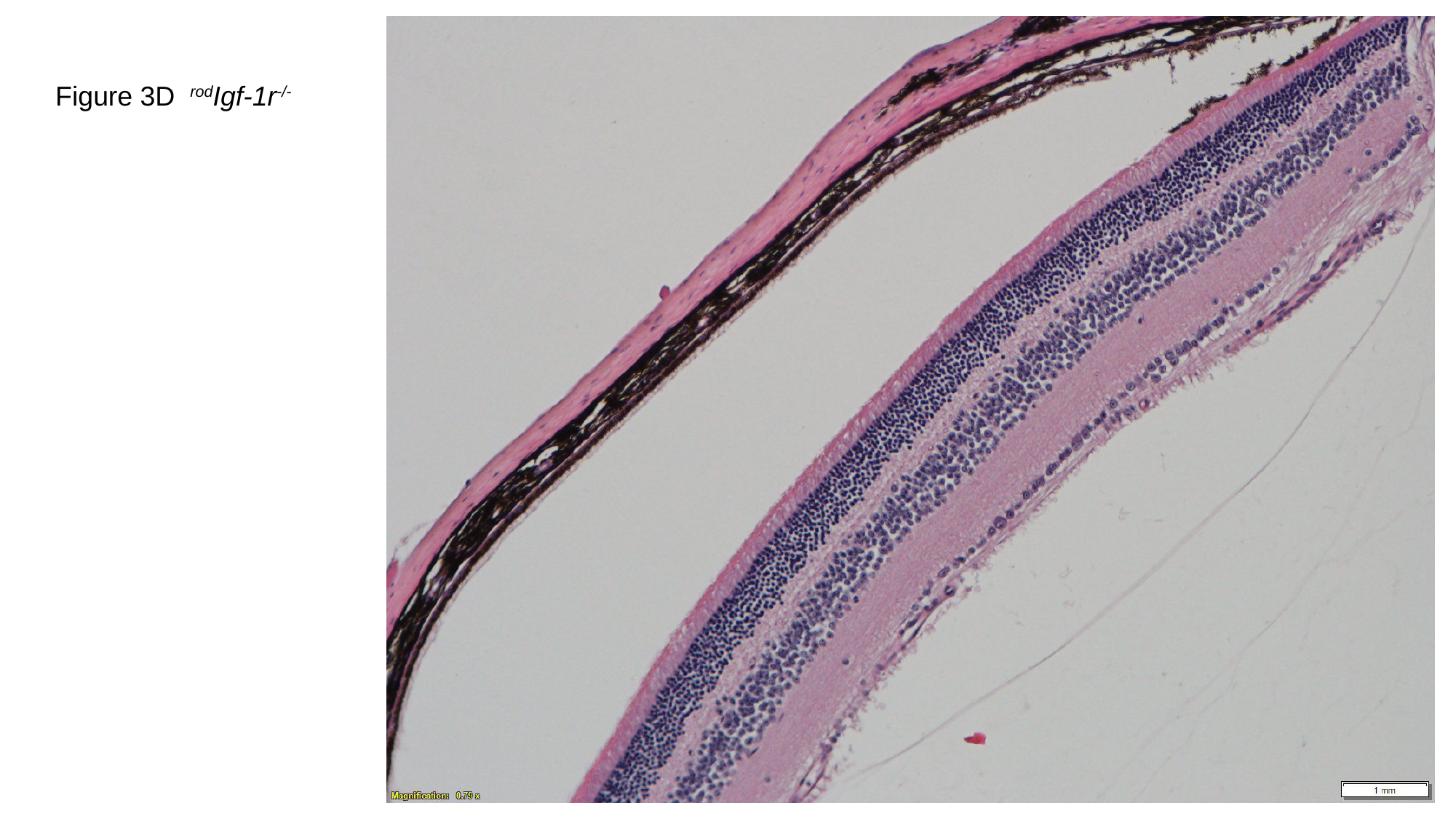

Figure 3D rodIgf-1r-/-

## Slide 17
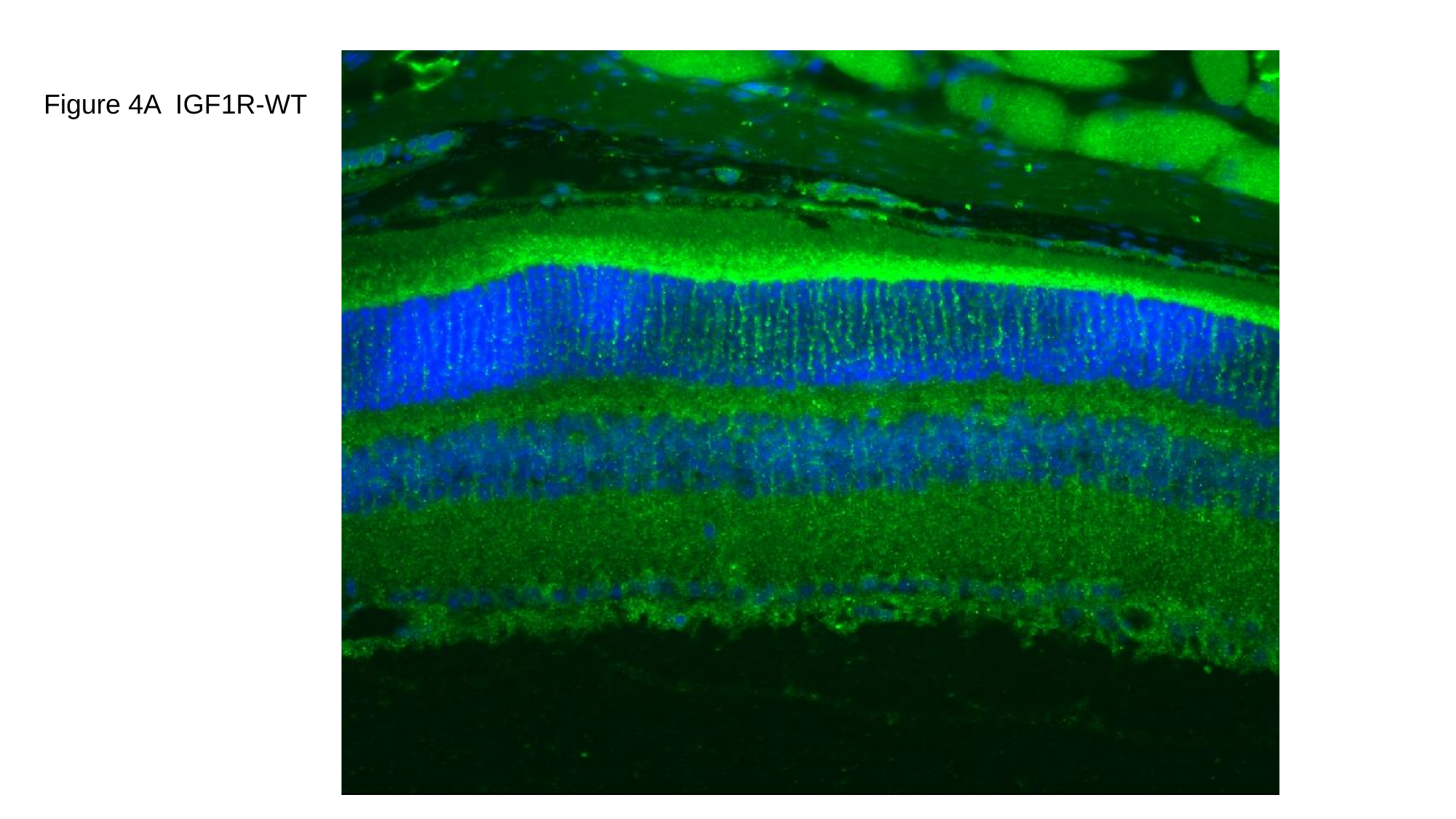

Figure 4A IGF1R-WT

## Slide 18
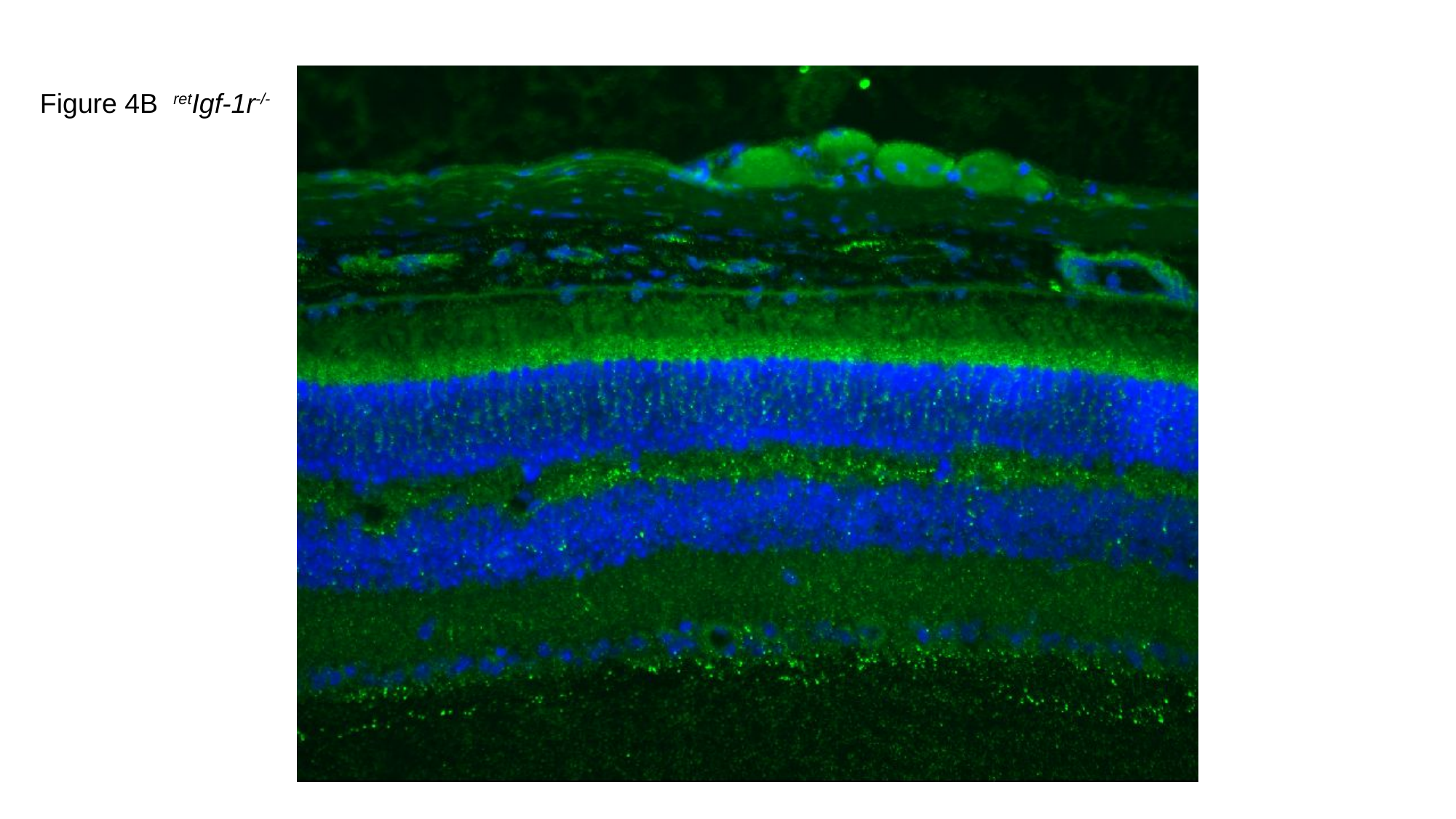

Figure 4B retIgf-1r-/-

## Slide 19
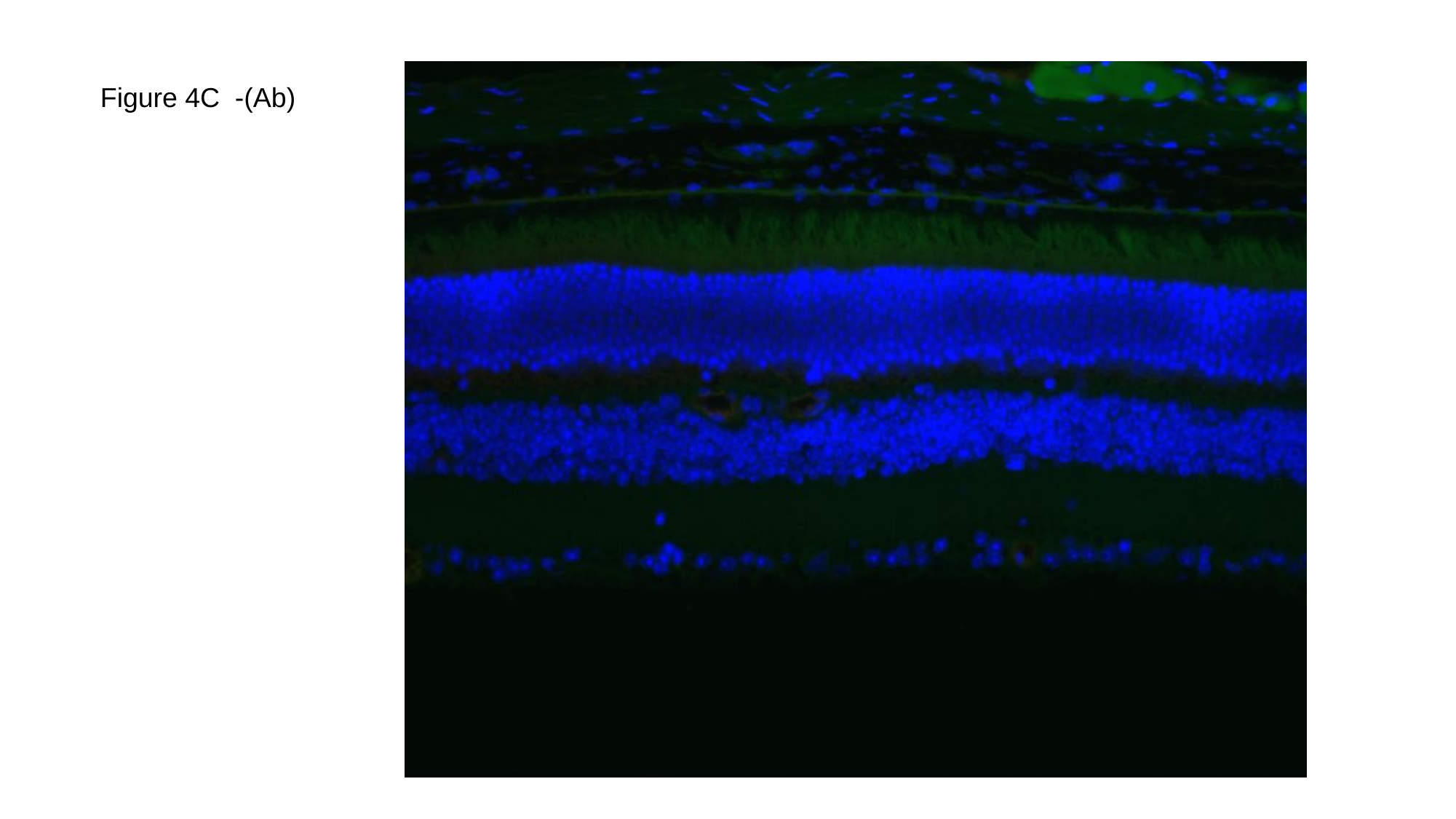

Figure 4C -(Ab)

## Slide 20
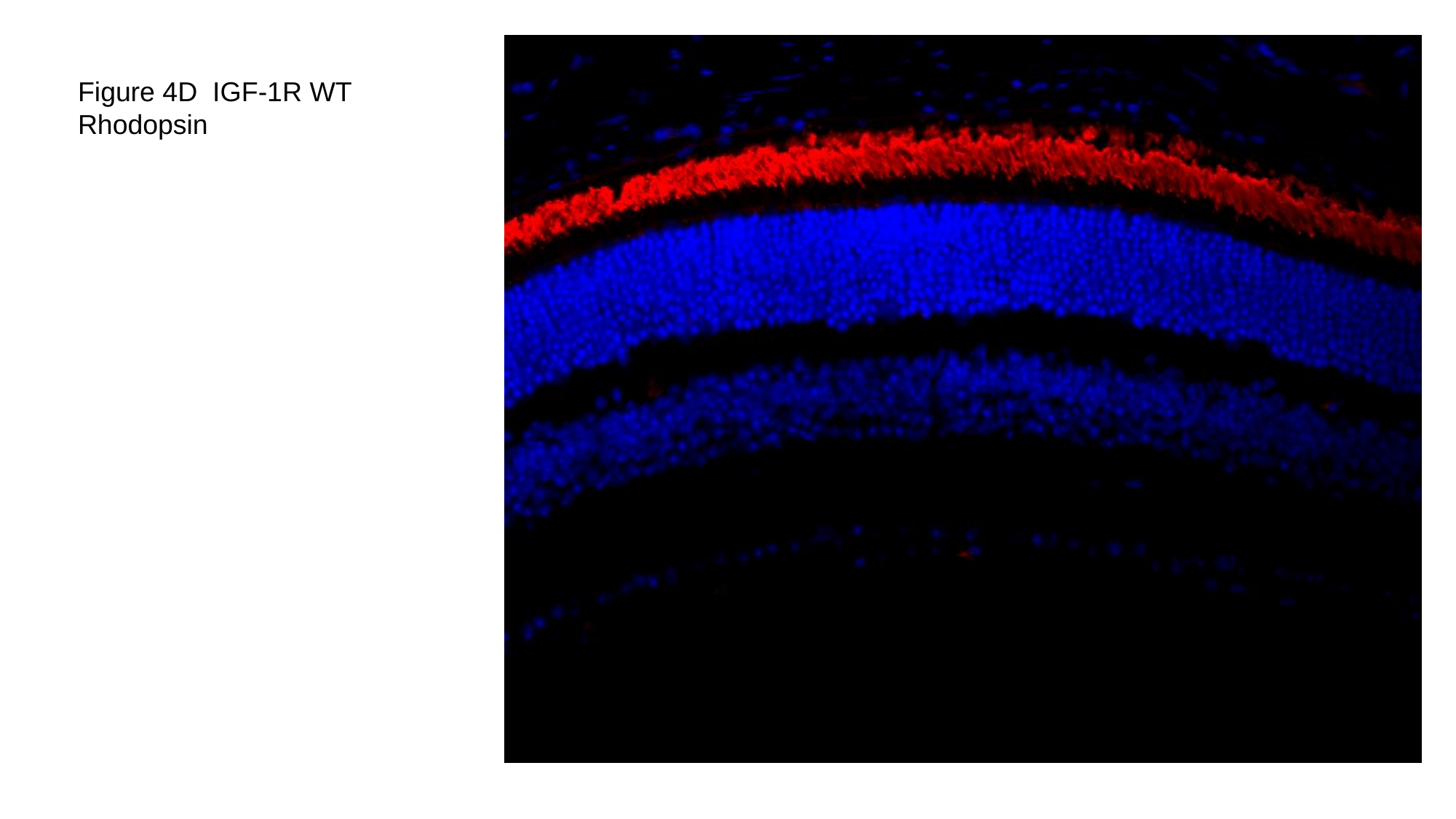

Figure 4D IGF-1R WT
Rhodopsin

## Slide 21
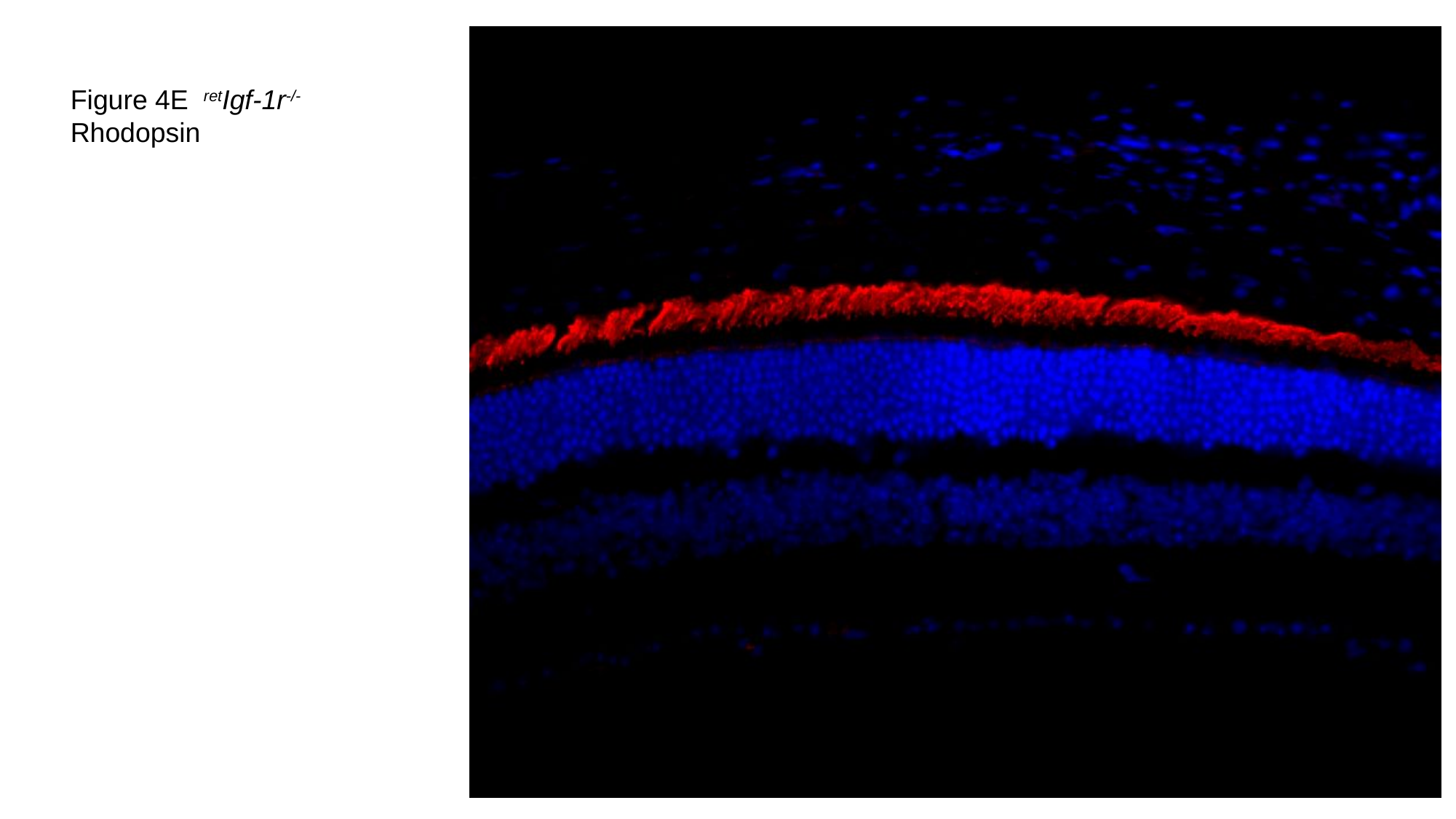

Figure 4E retIgf-1r-/-
Rhodopsin

## Slide 22
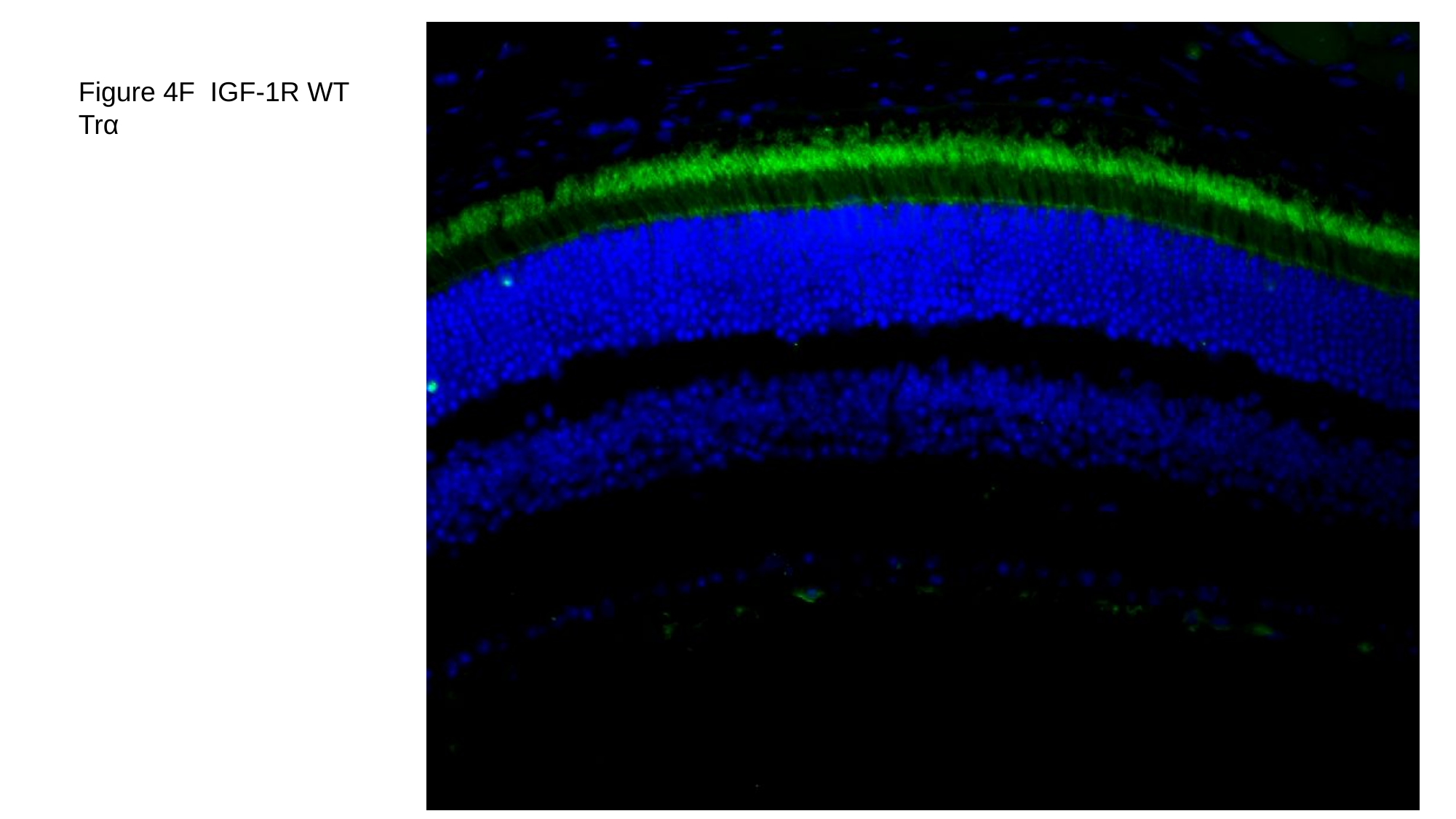

Figure 4F IGF-1R WT
Trα

## Slide 23
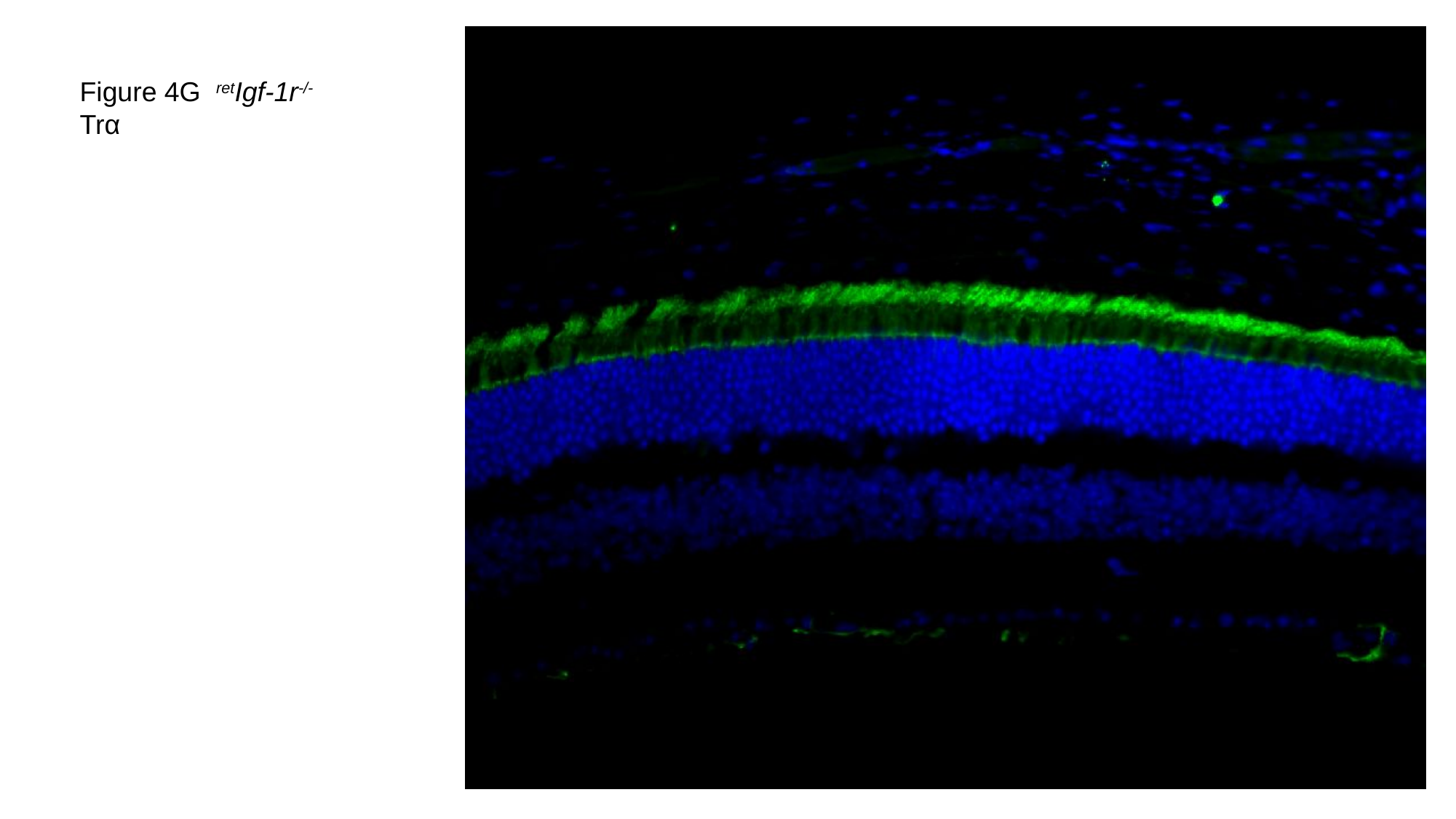

Figure 4G retIgf-1r-/-
Trα

## Slide 24
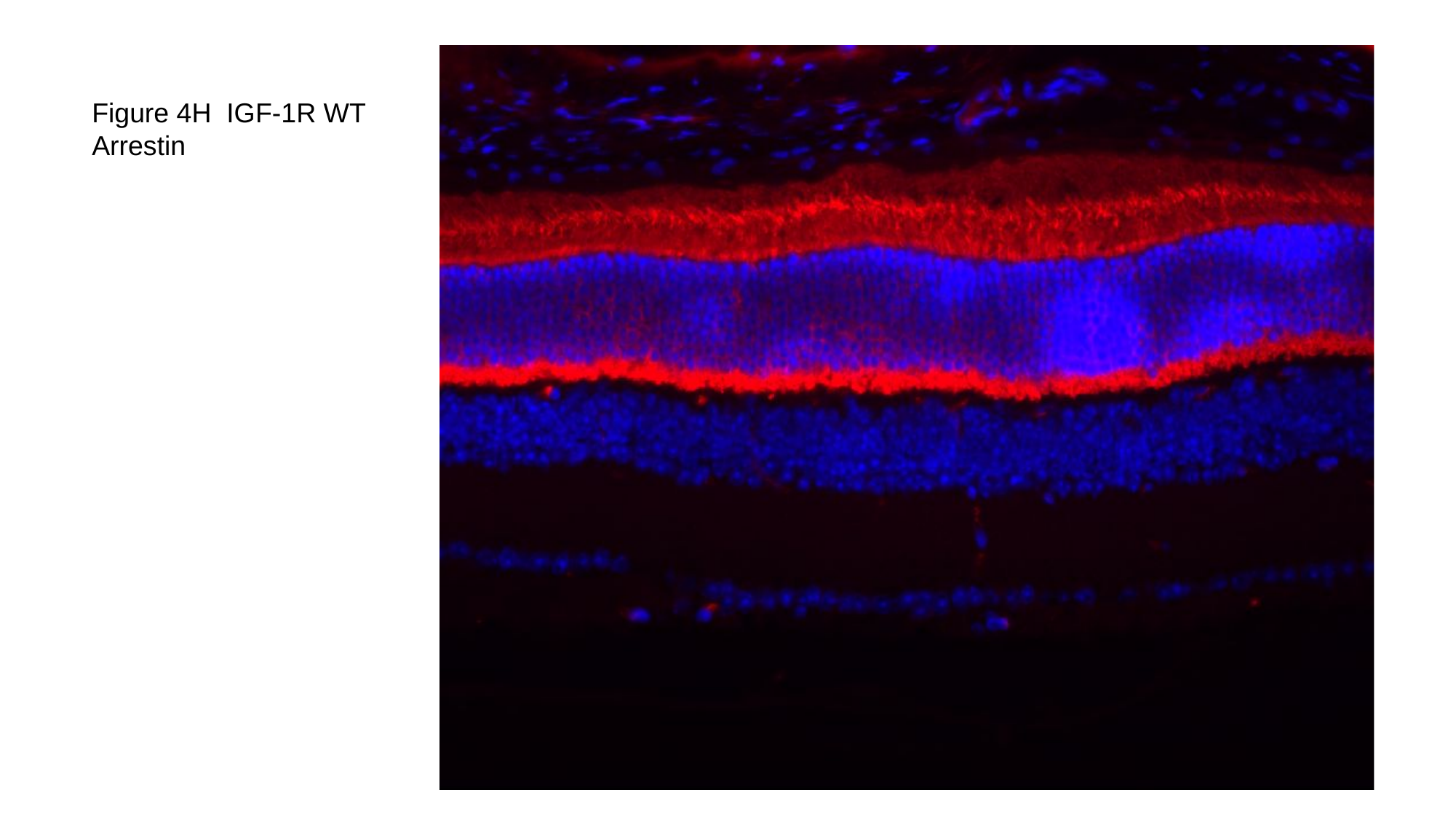

Figure 4H IGF-1R WT
Arrestin

## Slide 25
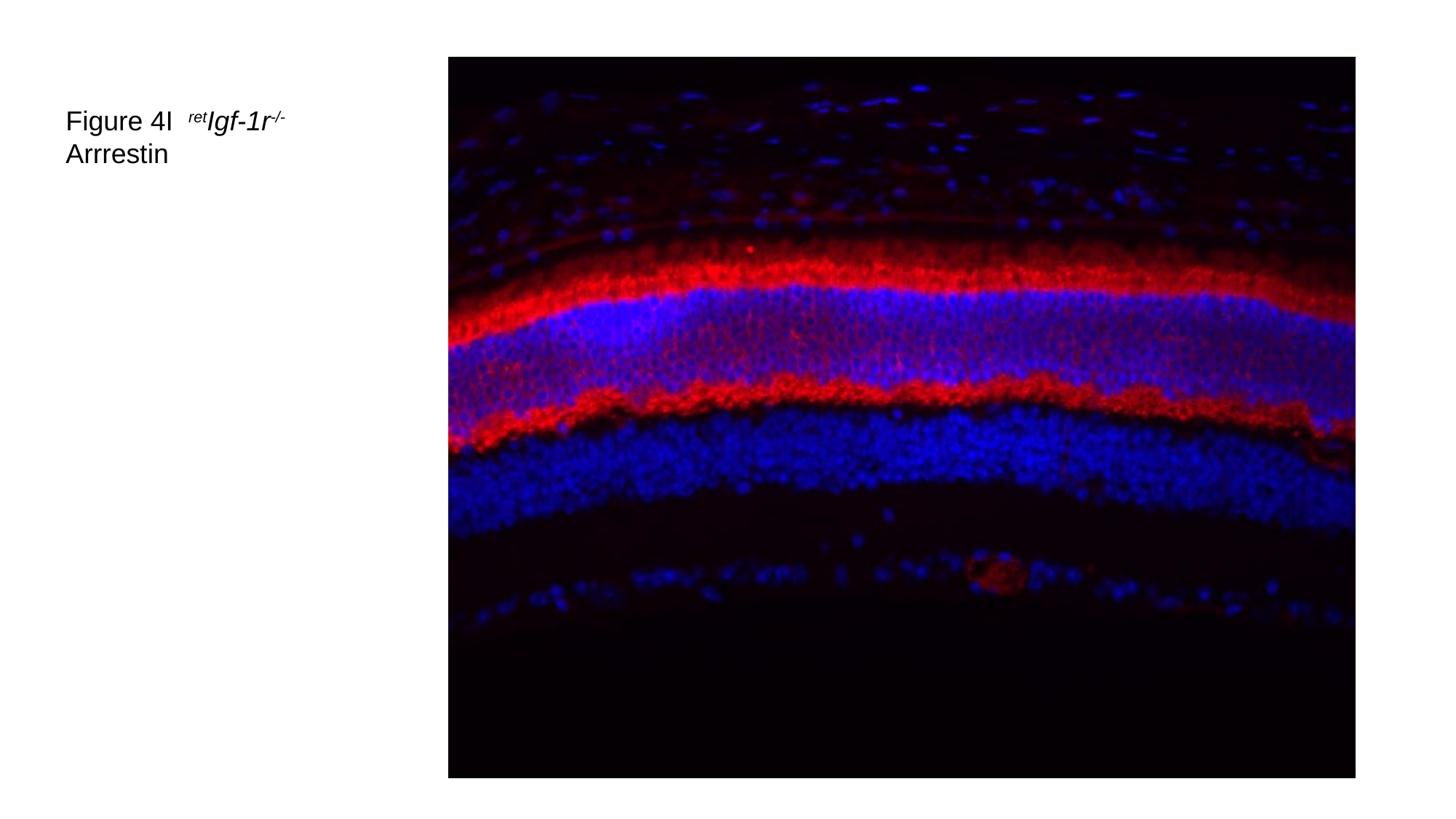

Figure 4I retIgf-1r-/-
Arrrestin

## Slide 26
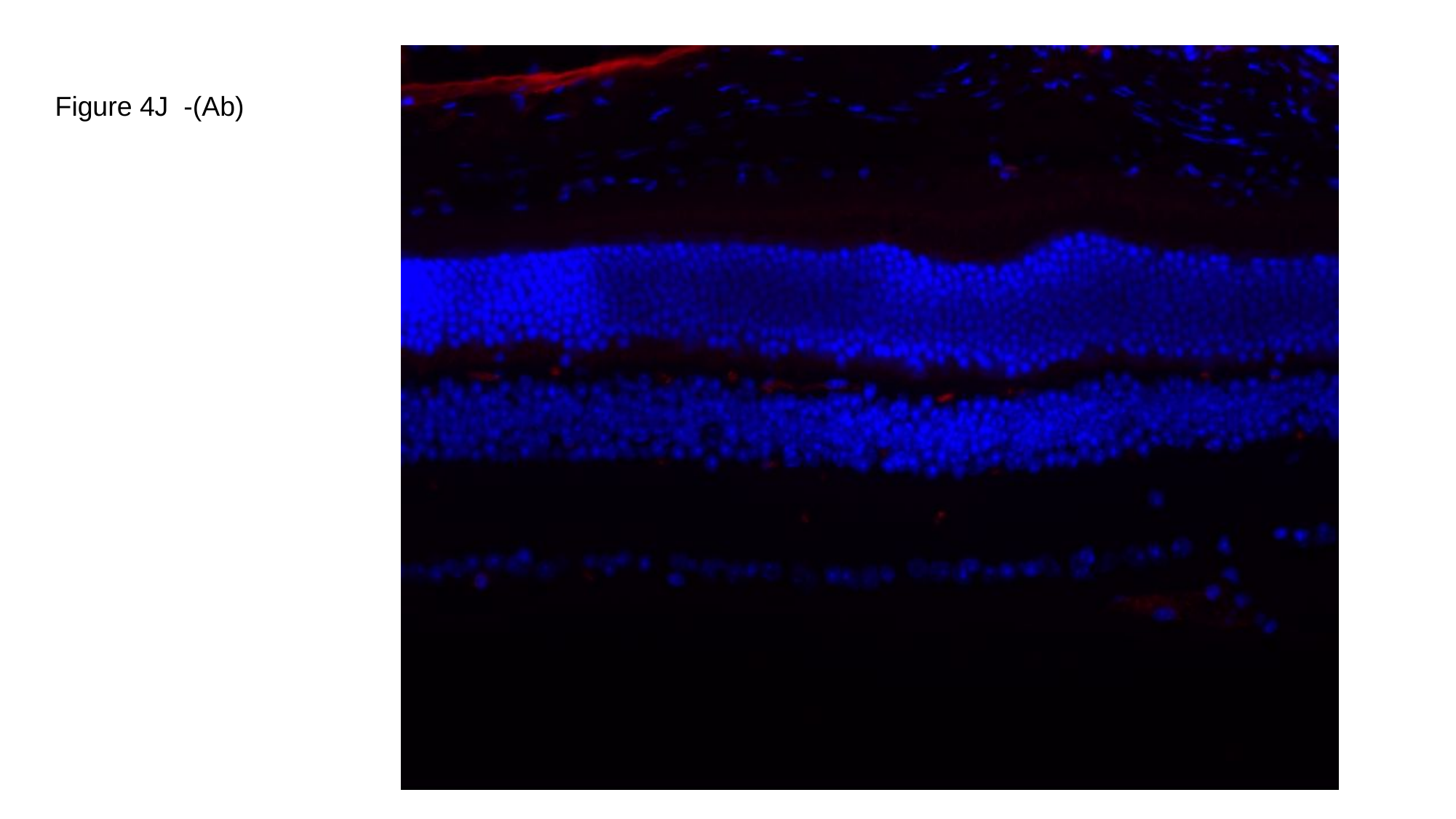

Figure 4J -(Ab)

## Slide 27
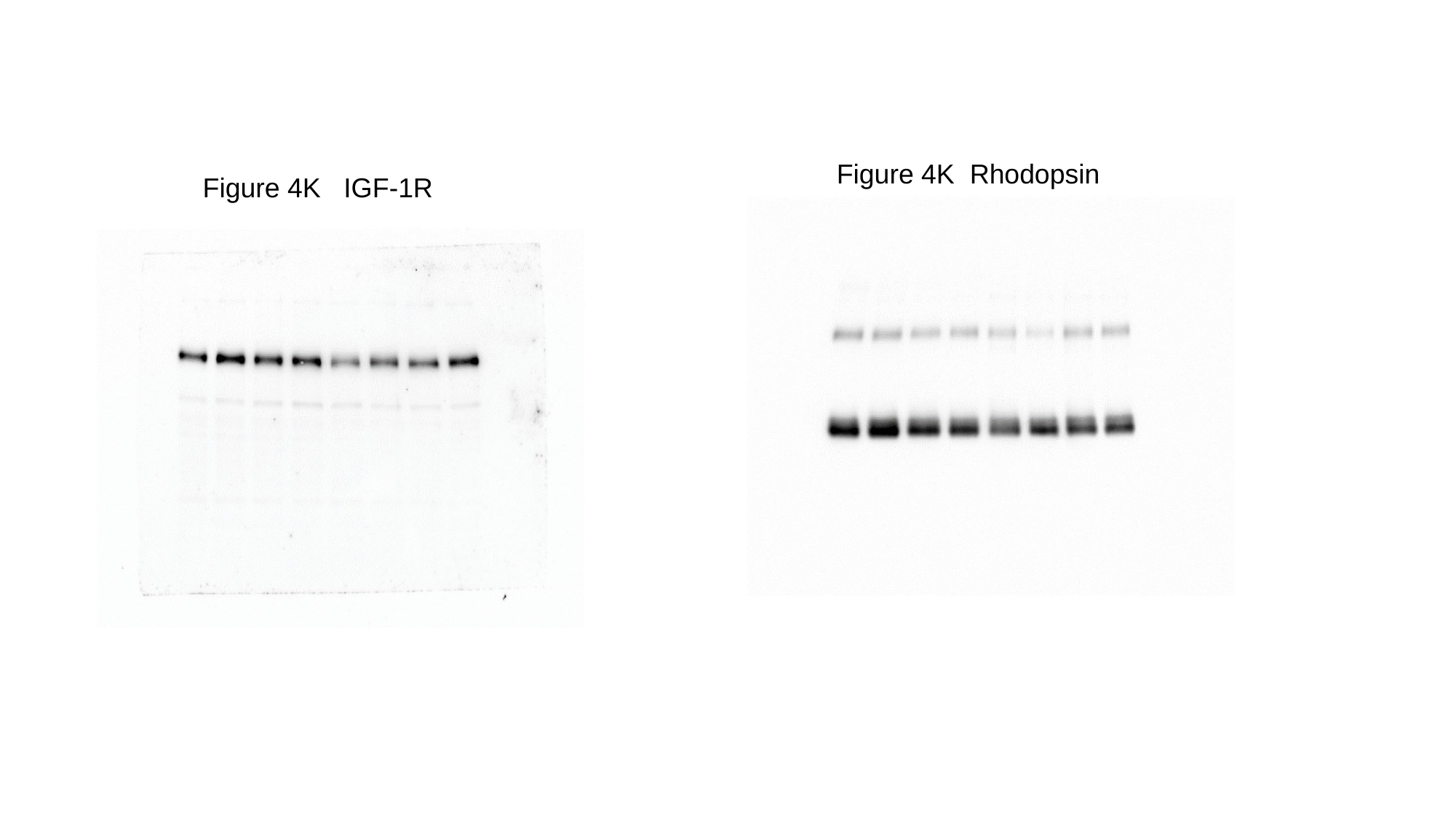

Figure 4K Rhodopsin
Figure 4K IGF-1R

## Slide 28
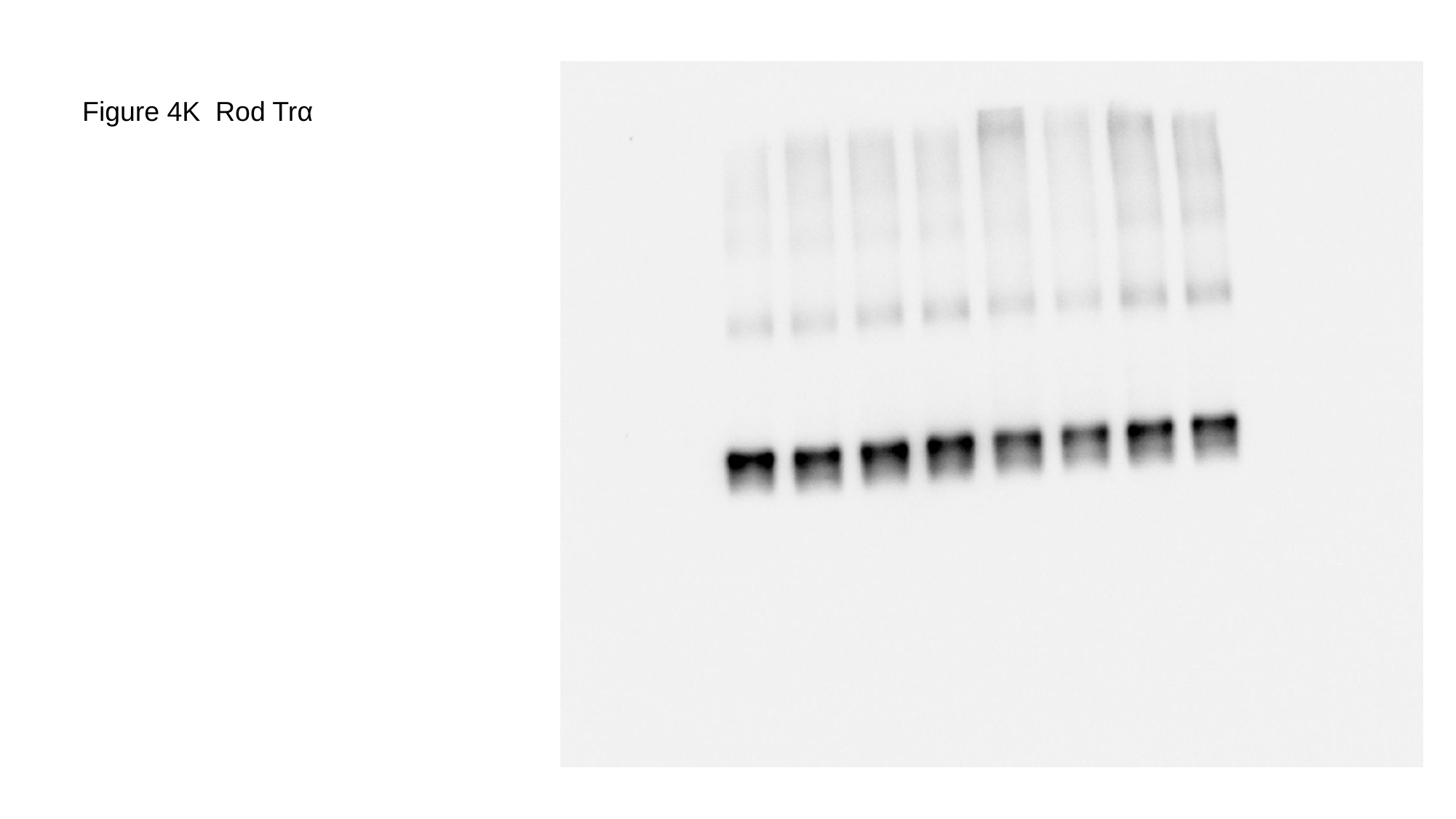

Figure 4K Rod Trα

## Slide 29
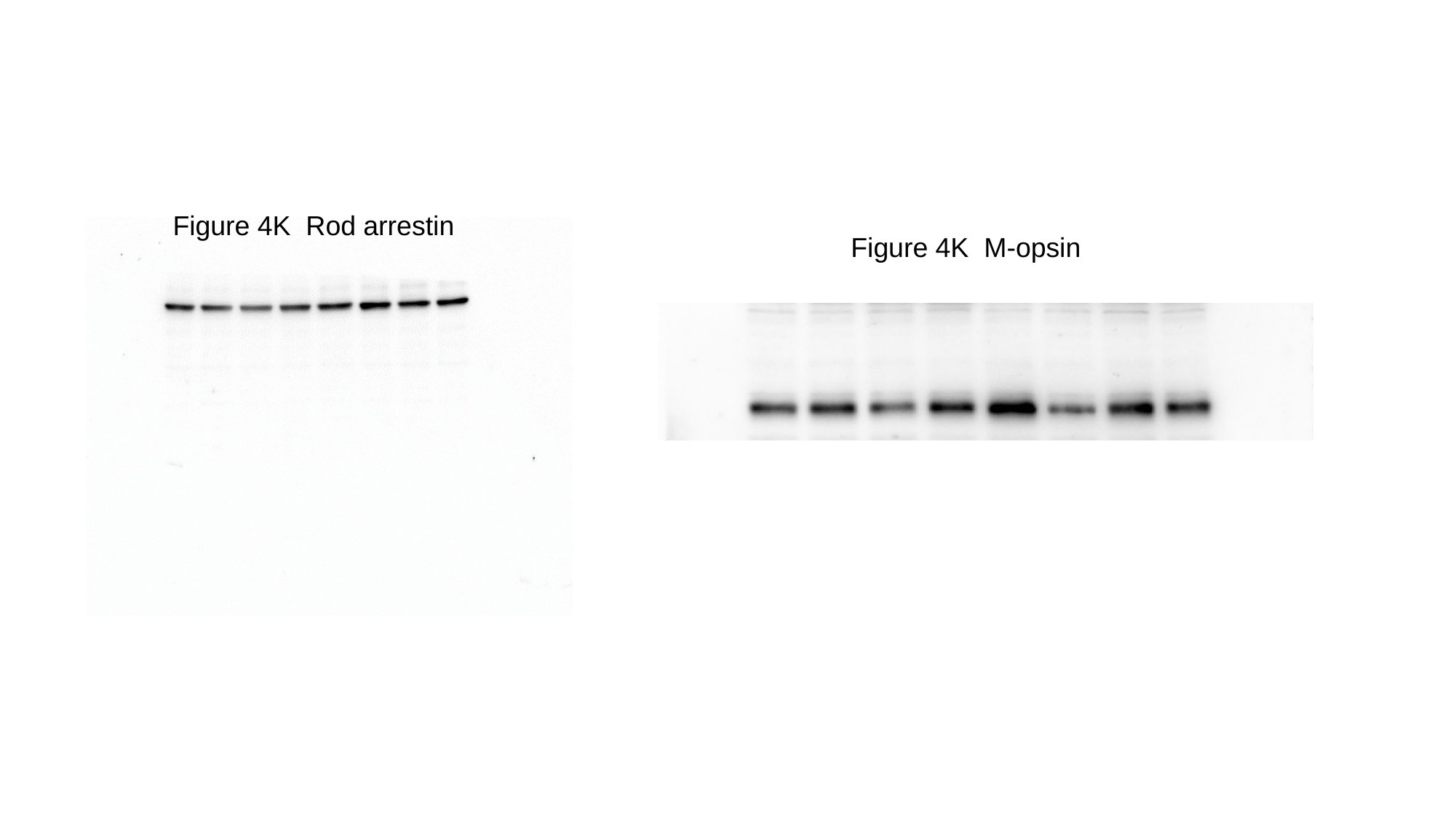

Figure 4K Rod arrestin
Figure 4K M-opsin

## Slide 30
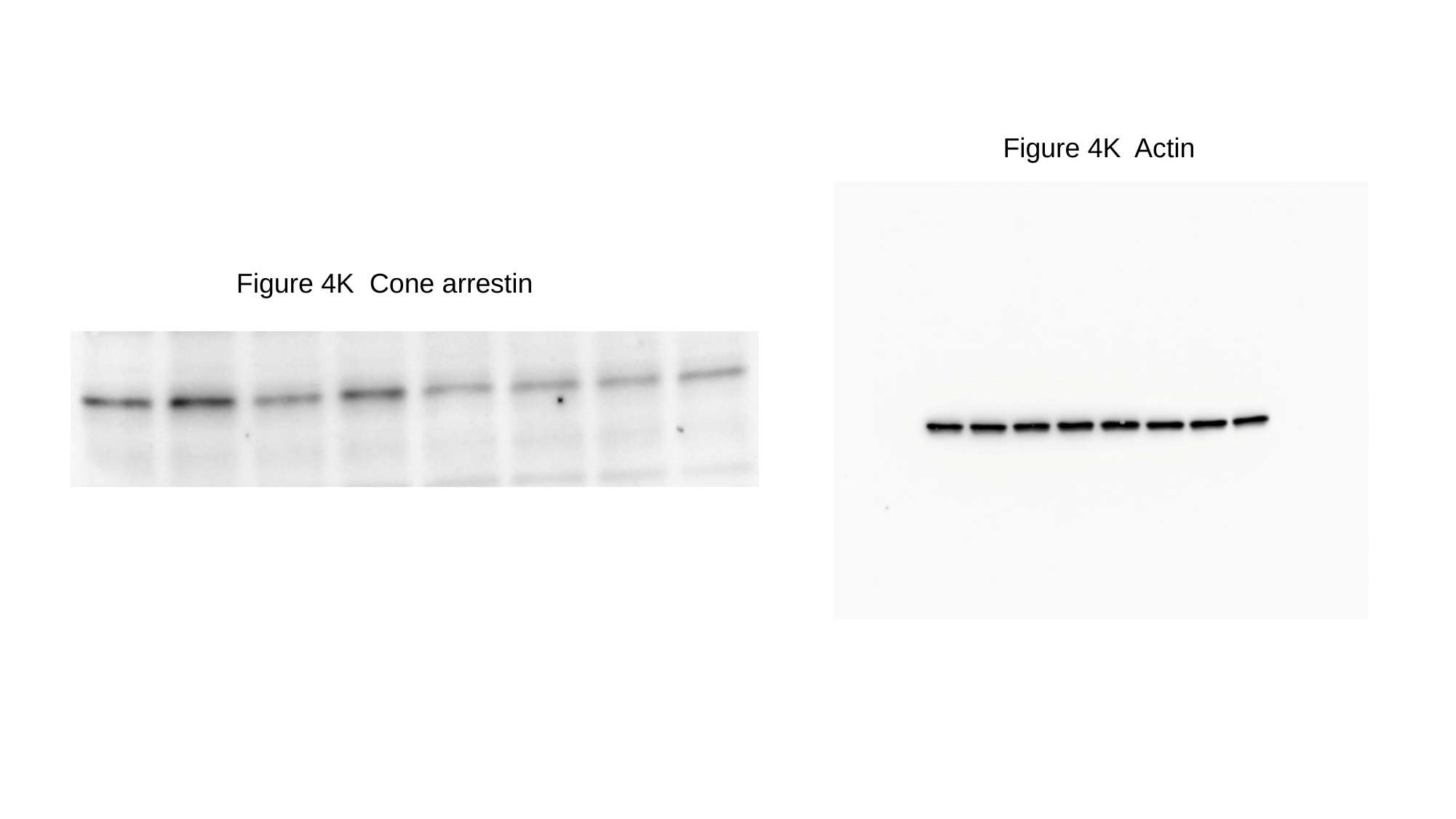

Figure 4K Actin
Figure 4K Cone arrestin

## Slide 31
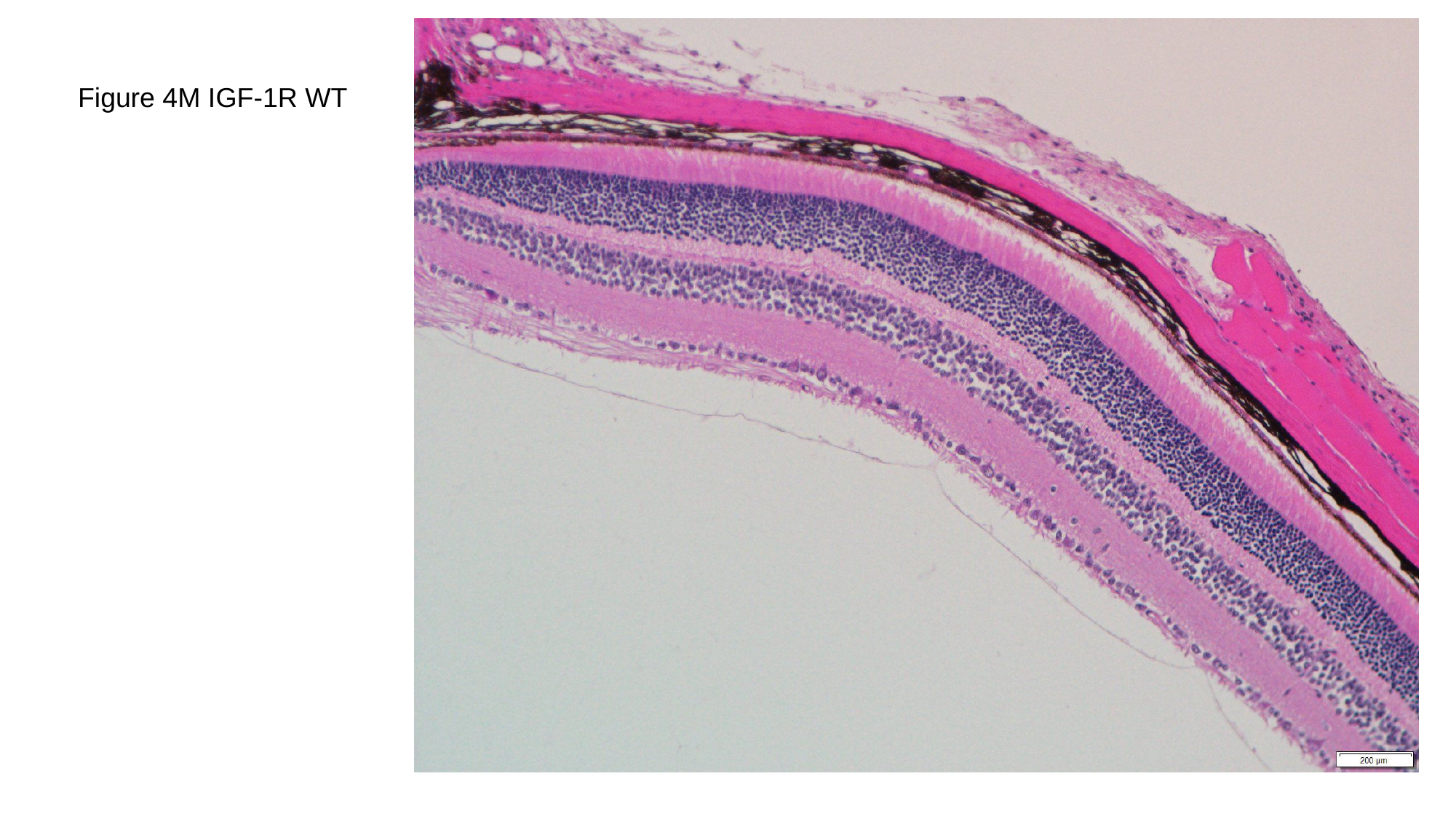

Figure 4M IGF-1R WT

## Slide 32
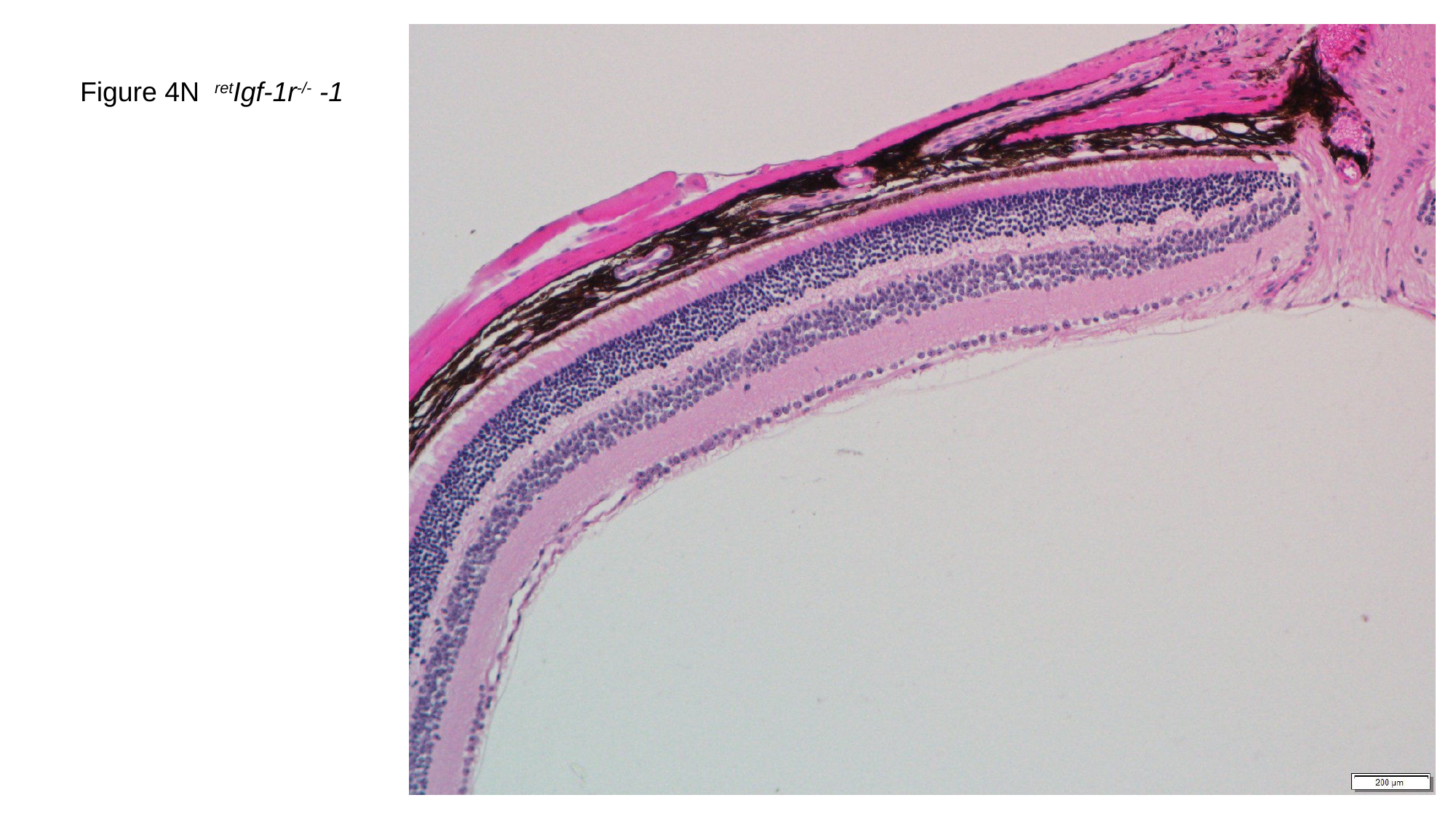

Figure 4N retIgf-1r-/- -1

## Slide 33
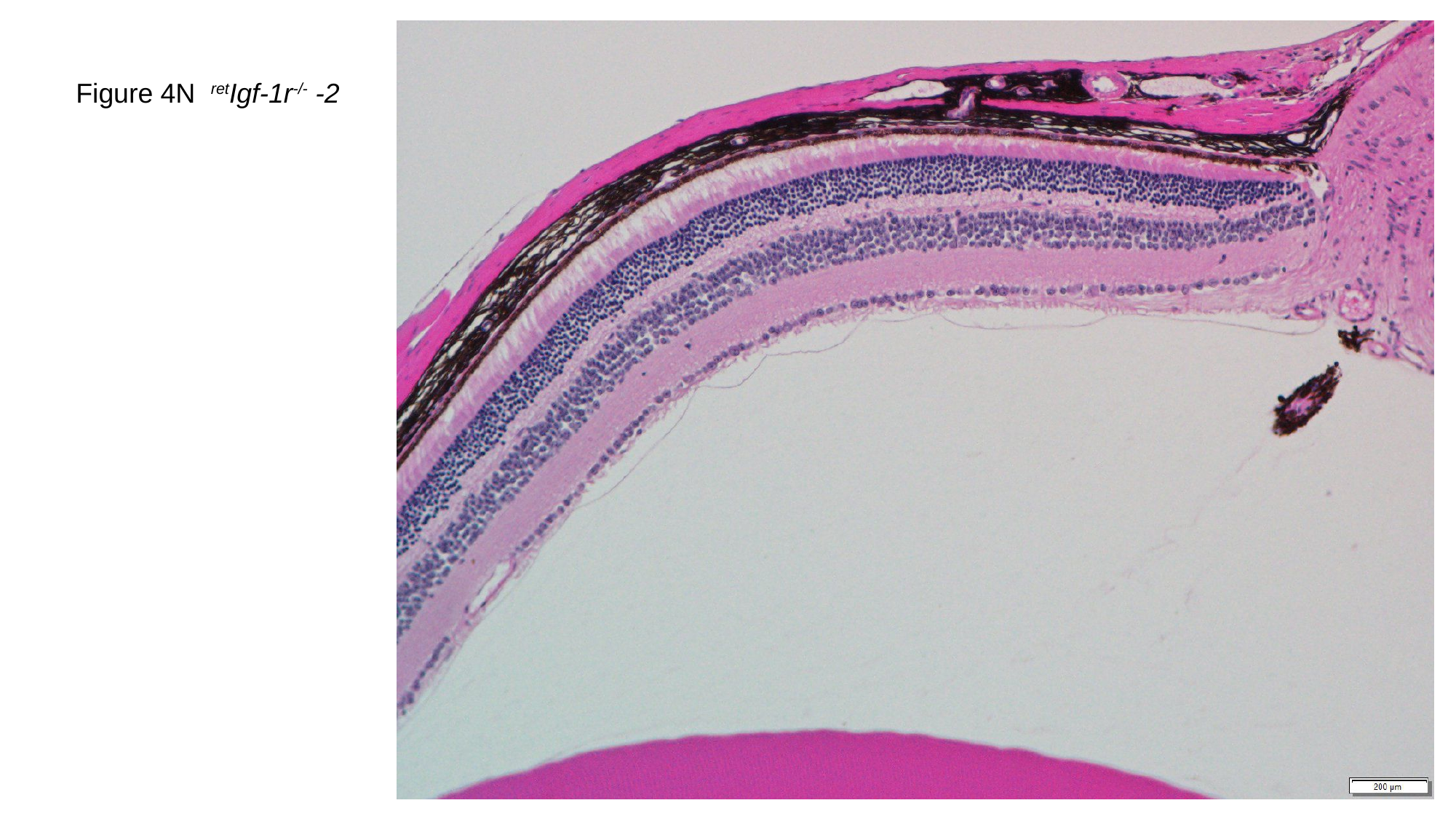

Figure 4N retIgf-1r-/- -2

## Slide 34
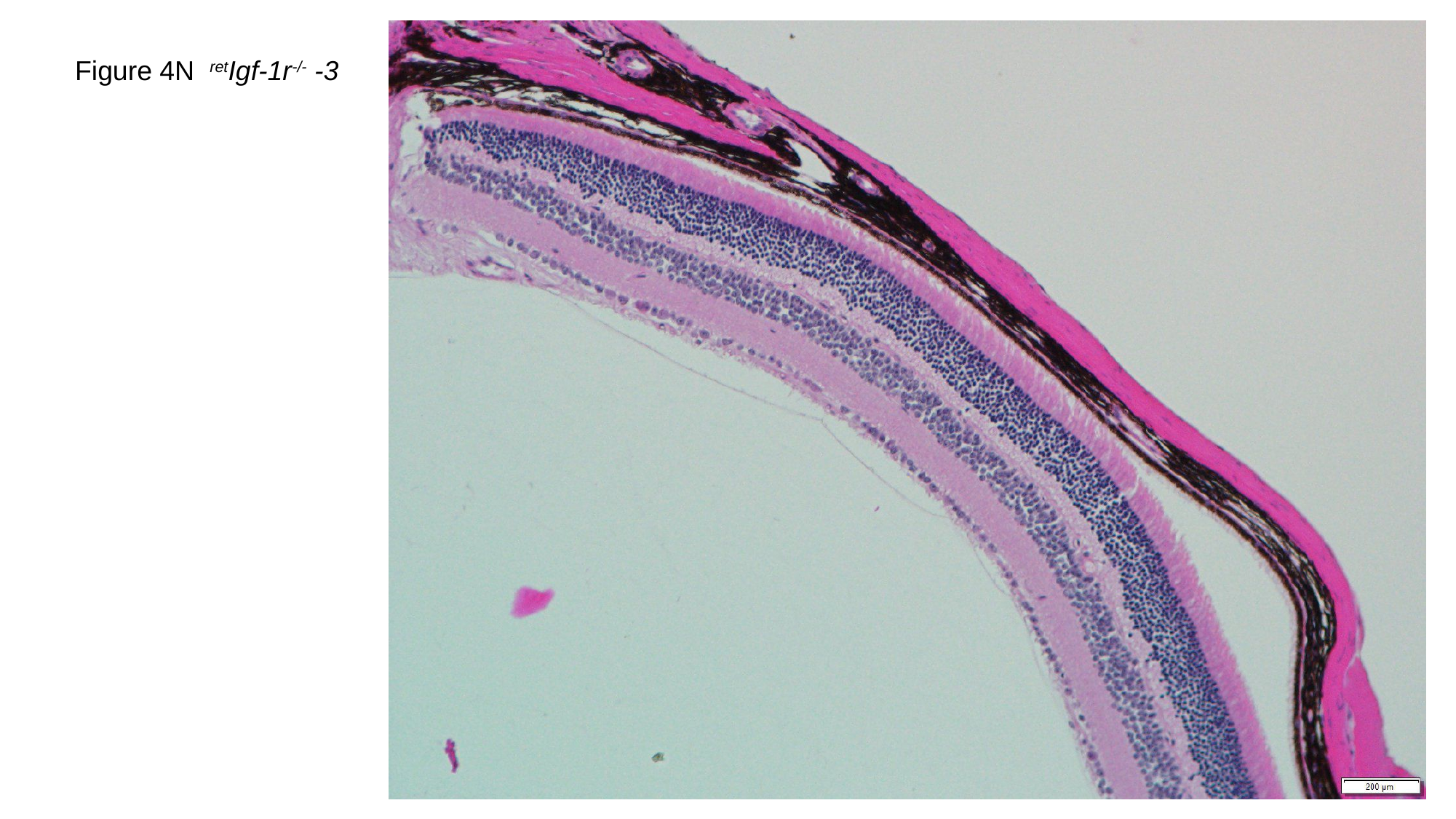

Figure 4N retIgf-1r-/- -3

## Slide 35
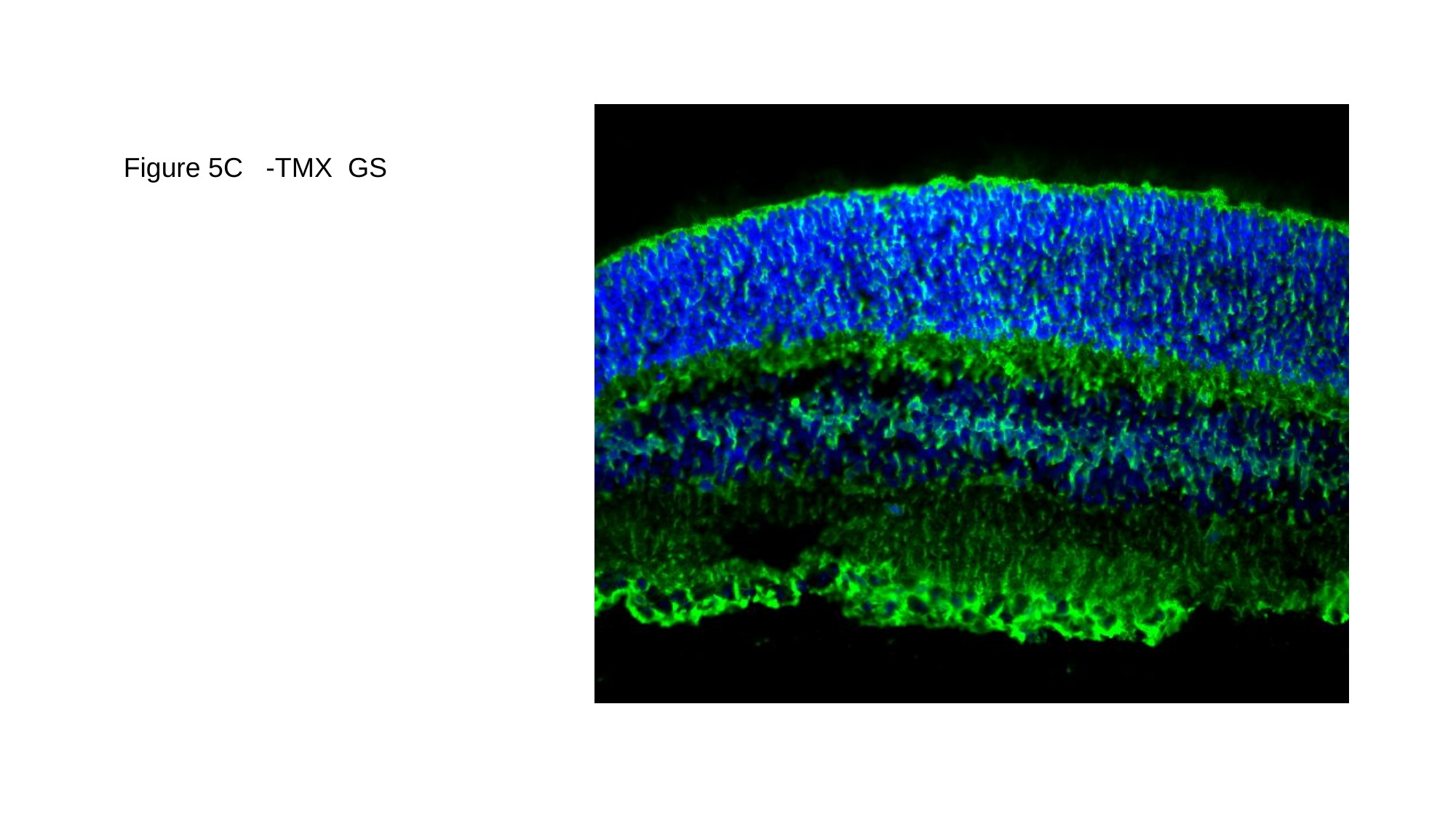

Figure 5C -TMX GS

## Slide 36
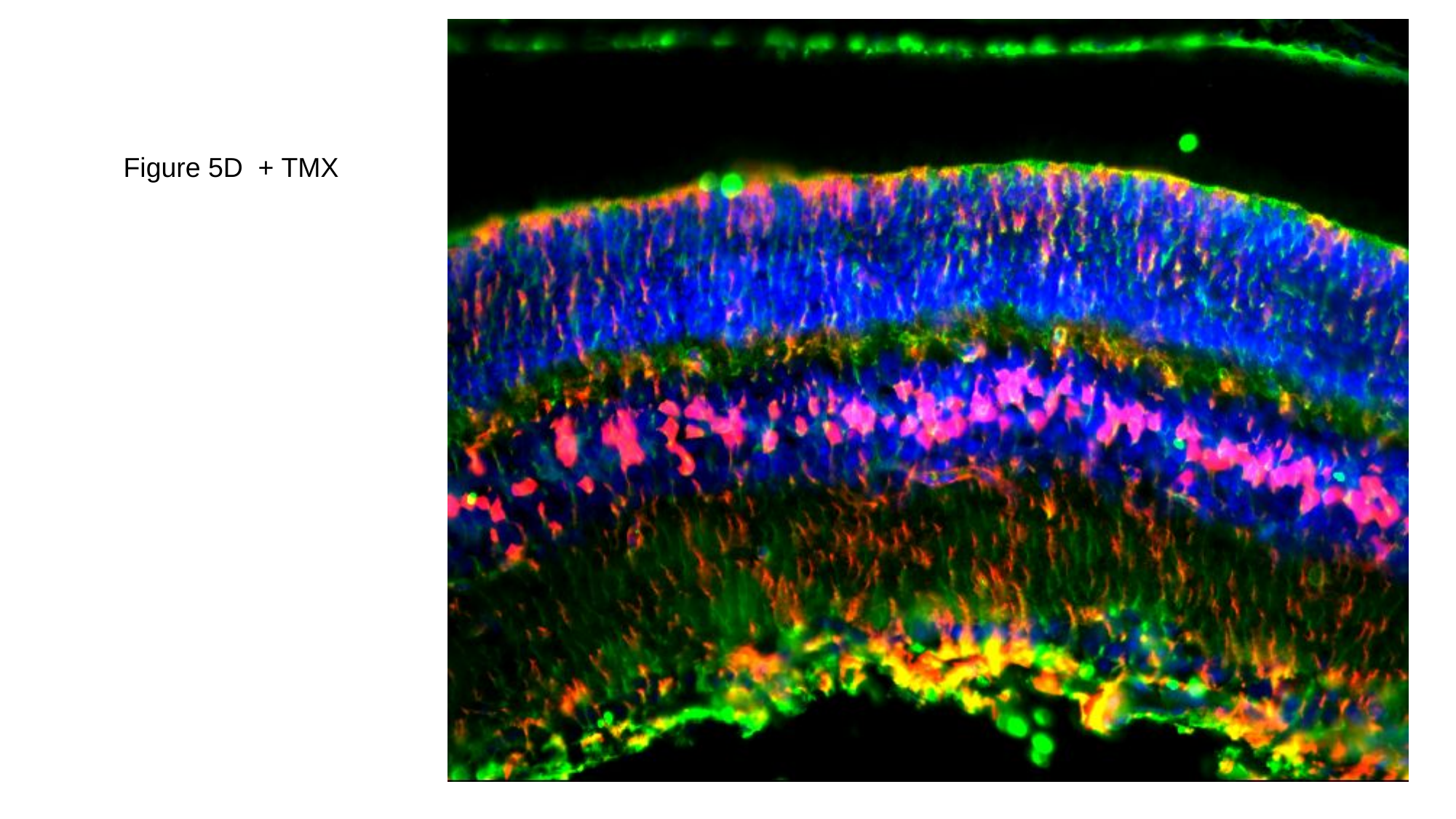

Figure 5D + TMX

## Slide 37
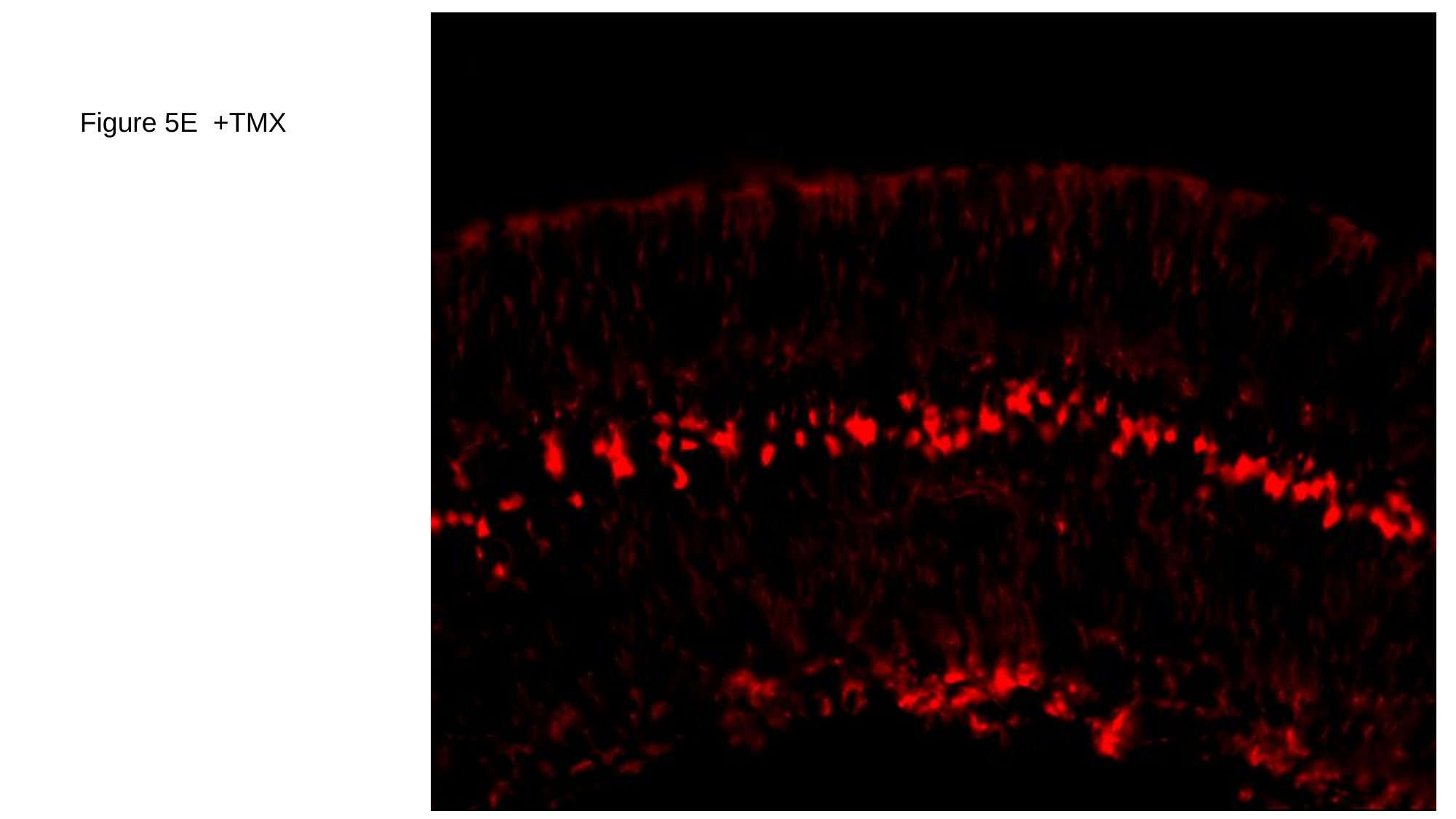

Figure 5E +TMX

## Slide 38
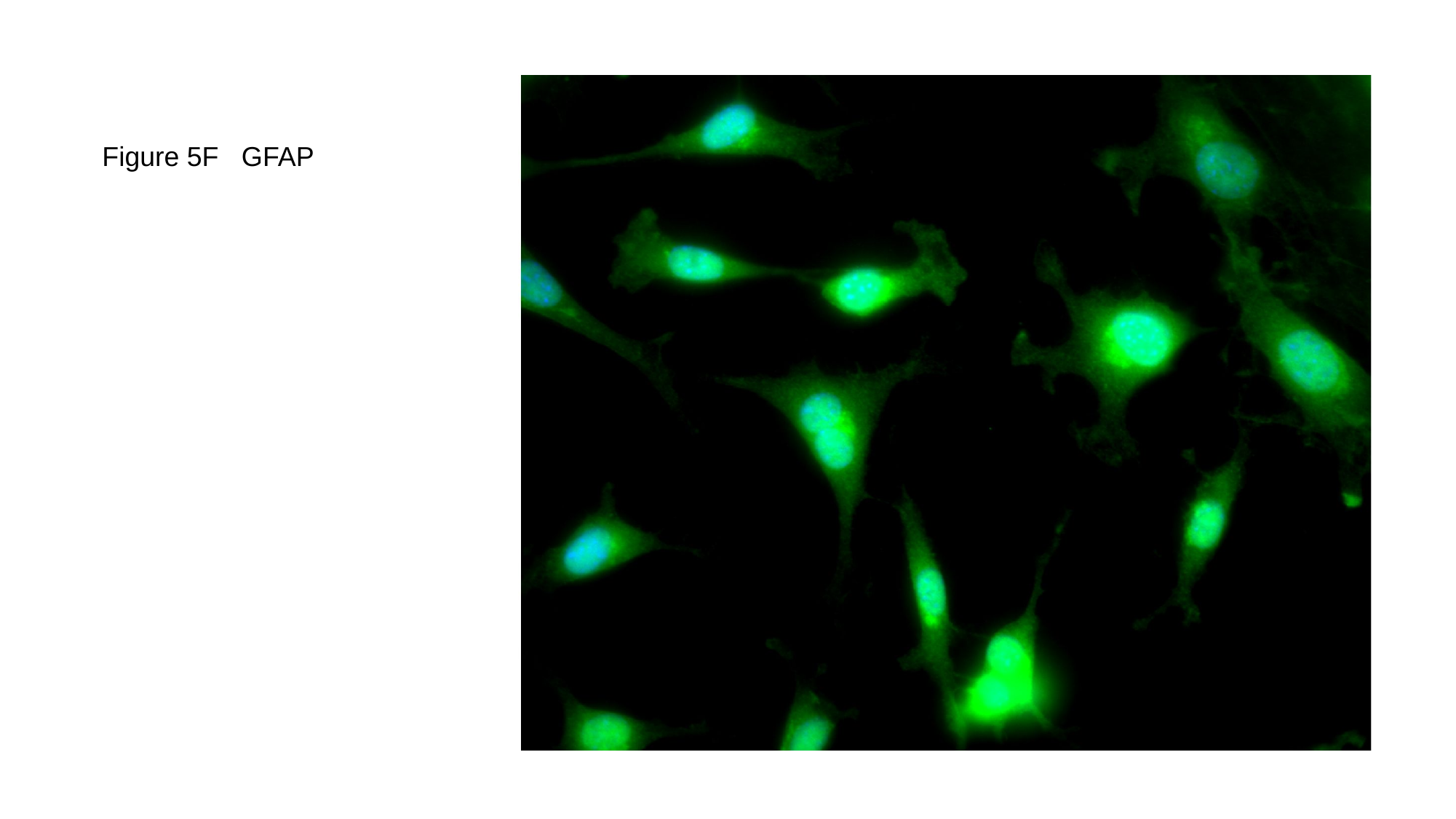

Figure 5F GFAP
