# Supplementary material for: Insulin-like growth factor 1 receptor mediates photoreceptor neuroprotection
Source: Cell Death Dis. 2022 Jul 15;13(7):613. doi: 10.1038/s41419-022-05074-3 (PMC9287313; doi:10.1038/s41419-022-05074-3)
Supplement: Supplementary file 3 — Author Contribution Statement [file 41419_2022_5074_MOESM3_ESM.docx]

RVSR designed the research. RVSR, AR, LP, MAB, and KT performed the research. RVSR, AR, KT, and LP analyzed the data. AB and TG analyzed the retinal metabolites. RVSR interpreted the results. RVSR wrote the paper.
