# Supplementary material for: Insulin-like growth factor 1 receptor mediates photoreceptor neuroprotection
Source: Cell Death Dis. 2022 Jul 15;13(7):613. doi: 10.1038/s41419-022-05074-3 (PMC9287313; doi:10.1038/s41419-022-05074-3)
Supplement: Supplementary file 4 — Supplementary Tables [file 41419_2022_5074_MOESM4_ESM.pdf]

Table S1: S1 Node Table

Id	Label	Degree	Betweenness
C00022	Pyruvic acid	227	44318.25
C00037	Glycine	134	19456.44
C00047	L-Lysine	111	12085.3
C00049	L-Aspartic acid	101	13171.8
C00249	Palmitic acid	86	12168.1
C00334	Gamma-Aminobutyric acid	81	12881.65
C02336	D-Fructose	38	2413.61
	Hexacosanoic acid	15	716.27
C00004	NADH	8	742.11
C00007	Oxygen	7	434.57
C00011	Carbon dioxide	7	406.74
C00008	ADP	7	175.07
C00116	Glycerol	7	175.07
C01330	Sodium	7	175.07
C01563	Carbamic acid	6	792.83
C00002	Adenosine triphosphate	6	98.96
C00020	Adenosine monophosphate	6	98.96
C03878	Beta-N-Acetylglucosamine	6	98.96
C00305	Magnesium	6	98.96
C00006	NADP	6	68.57
C00044	Guanosine triphosphate	6	68.57
C01089	(R)-3-Hydroxybutyric acid	5	399.14
C00042	Succinic acid	5	205.92
C00163	Propionic acid	5	45.64
C00010	Coenzyme A	5	22.93
C00035	Guanosine diphosphate	5	22.93
C00062	L-Arginine	5	22.93
C00077	Ornithine	5	22.93
C00158	Citric acid	5	22.93
C00051	Glutathione	5	22.93
C00026	Oxoglutaric acid	5	14.42
C00027	Hydrogen peroxide	5	14.42
C00025	L-Glutamic acid	5	14.42
C00079	L-Phenylalanine	5	14.42
C00018	Pyridoxal 5'-phosphate	5	14.42
C00041	L-Alanine	5	14.42
C00217	D-Glutamic acid	5	14.42
C00099	Beta-Alanine	5	14.42
C00123	L-Leucine	5	14.42
C00209	Oxalic acid	4	176.17
C02166	Leukotriene C4	4	68.57
C00036	Oxalacetic acid	4	48.79
C00111	Dihydroxyacetone phosphate	4	38.81
C00015	Uridine 5'-diphosphate	4	34.57
C00114	Choline	4	31.22

C00084	Acetaldehyde	4	22.93
C00134	Putrescine	4	14.42
C00078	L-Tryptophan	4	0
C00188	L-Threonine	4	0
C00065	L-Serine	4	0
C00097	L-Cysteine	4	0
C00399	Ubiquinone-1	4	0
C00740	D-Serine	4	0
C05519	L-Allothreonine	4	0
C00091	Succinyl-CoA	3	124.39
C00332	Acetoacetyl-CoA	3	119.27
C00469	Ethanol	3	55.06
C00075	Uridine triphosphate	3	55.06
C00780	Serotonin	3	45.64
C00584	Prostaglandin E2	3	45.64
C01996	Acetylcholine	3	45.64
C00547	Norepinephrine	3	45.64
C00388	Histamine	3	45.64
C06314	Lipoxin A4	3	45.64
C00788	Epinephrine	3	45.64
C00089	Sucrose	3	38.81
C00031	D-Glucose	3	34.57
C01172	Beta-D-Glucose 6-phosphate	3	34.57
C00092	Glucose 6-phosphate	3	34.57
C00159	D-Mannose	3	24.58
C00221	Beta-D-Glucose	3	24.58
C00093	Glycerol 3-phosphate	3	24.58
C00962	Beta-D-Galactose	3	24.58
C00016	FAD	3	22.93
C00061	Flavin Mononucleotide	3	22.93
C00086	Urea	3	14.42
C00383	Malonic acid	3	14.42
C00489	Glutaric acid	3	14.42
C01384	Maleic acid	3	14.42
C00245	Taurine	3	0
C00100	Propionyl-CoA	3	0
C00064	L-Glutamine	3	0
C00048	Glyoxylic acid	3	0
C00441	L-Aspartate-semialdehyde	3	0
C00021	S-Adenosylhomocysteine	3	0
C00024	Acetyl-CoA	3	0
C00192	Hydroxylamine	3	0
C00227	Acetylphosphate	3	0
C00741	Diacetyl	3	0
C00073	L-Methionine	3	0
C00119	Phosphoribosyl pyrophosphate	3	0
C00135	L-Histidine	3	0

C00407	L-Isoleucine	3	0
C00742	Fluoride	3	0
C00250	Pyridoxal	3	0
C00246	Butyric acid	3	0
C00183	L-Valine	3	0
C00263	L-Homoserine	3	0
C00147	Adenine	2	24.58
C01083	Trehalose	2	24.58
C00354	Fructose 1,6-bisphosphate	2	24.58
C00794	Sorbitol	2	24.58
C00379	D-Xylitol	2	24.58
C05345	Beta-D-Fructose 6-phosphate	2	24.58
C00392	Mannitol	2	24.58
C00695	Cholic acid	2	22.93
C00315	Spermidine	2	0
C01672	Cadaverine	2	0
C00390	QH2	2	0
C00101	Tetrahydrofolic acid	2	0
C00082	L-Tyrosine	2	0
C01589	Imidazole	2	0
C00144	Guanosine monophosphate	2	0
C00182	Glycogen	2	0
C00267	Alpha-D-Glucose	2	0
C00179	Agmatine	2	0
C06153	scyllo-Inositol	2	0
C00265	1,4-Dithiothreitol	2	0
C03283	L-2,4-diaminobutyric acid	2	0
C02198	Thromboxane A2	2	0
C00647	Pyridoxamine 5'-phosphate	2	0
C00063	Cytidine triphosphate	2	0
C00258	Glyceric acid	2	0
C00631	2-Phospho-D-glyceric acid	2	0
C00750	Spermine	2	0
C00083	Malonyl-CoA	2	0
C00712	Oleic acid	2	0
C00440	5-Methyltetrahydrofolic acid	2	0
C00180	Benzoic acid	2	0
C01475	Allysine	2	0
C00232	Succinic acid semialdehyde	2	0
C02165	Leukotriene B4	2	0
C00160	Glycolic acid	2	0
C00168	Hydroxypyruvic acid	2	0
C00534	Pyridoxamine	2	0
C00133	D-Alanine	2	0
C02679	Dodecanoic acid	2	0
C00067	Formaldehyde	2	0
C00283	Hydrogen sulfide	2	0

C00430	5-Aminolevulinic acid	2	0
C00149	L-Malic acid	2	0
C00109	2-Ketobutyric acid	2	0
C00497	D-Malic acid	2	0
C00248	Lipoamide	2	0
C00352	Glucosamine 6-phosphate	2	0
C01845	Isopropyl alcohol	2	0
C00130	Inosinic acid	2	0
C00108	2-Aminobenzoic acid	2	0
C01005	Phosphoserine	2	0
C00143	5,10-Methylene-THF	2	0
C01746	Piperidine	2	0
C00555	4-Aminobutyraldehyde	2	0
C16537	Pentadecanoic acid	2	0
C01102	O-Phosphohomoserine	2	0
C00193	Benzaldehyde	2	0
C00148	L-Proline	2	0
C00402	D-Aspartic acid	2	0
C06007	(R) 2,3-Dihydroxy-3-methylvalerate	2	0
C06006	(S)-2-Aceto-2-hydroxybutanoic acid	2	0
C00152	L-Asparagine	2	0
C00644	Mannitol 1-phosphate	2	0
C06010	(S)-2-Acetolactate	2	0
C04272	(R)-2,3-Dihydroxy-isovalerate	2	0
C03972	Tetrahydrodipicolinate	2	0
C06104	Adipic acid	1	0
C00356	3-Hydroxy-3-methylglutaryl-CoA	1	0
C02214	Glutaconic acid	1	0
C00517	Palmitaldehyde	1	0
C05337	Chenodeoxycholoyl-CoA	1	0
C05449	3a,7a-Dihydroxy-5b-24-oxocholestanoyl-CoA	1	0
C00156	4-Hydroxybenzoic acid	1	0
C00154	Palmityl-CoA	1	0
C01794	Choloyl-CoA	1	0
C00234	10-Formyltetrahydrofolate	1	0
C00272	Tetrahydrobiopterin	1	0
C03758	Dopamine	1	0
C00166	Phenylpyruvic acid	1	0
C12147	O-Phosphothreonine	1	0
C05402	Melibiose	1	0
C00535	Testosterone	1	0
C01451	Salicin	1	0
C00689	Trehalose 6-phosphate	1	0
C00043	Uridine diphosphate-N-acetylglucosamine	1	0
C00105	Uridine 5'-monophosphate	1	0
C00085	Fructose 6-phosphate	1	0
C00588	Phosphorylcholine	1	0

C08320	Tetracosanoic acid	1	0
C00575	Cyclic AMP	1	0
C01074	N-Acetylgalactosamine	1	0
C01137	S-Adenosylmethioninamine	1	0
C00219	Arachidonic acid	1	0
C01595	Linoleic acid	1	0
C00696	Prostaglandin D2	1	0
C13856	2-Arachidonylglycerol	1	0
C05467	3a,7a,12a-Trihydroxy-5b-24-oxocholestanoyl-(	1	0
C00251	Chorismate	1	0
C00661	D-Glyceraldehyde 3-phosphate	1	0
C00546	Pyruvaldehyde	1	0
C00568	p-Aminobenzoic acid	1	0
C00169	Carbamoyl phosphate	1	0
C00437	N-Acetylornithine	1	0
C16699	2-O-(6-Phospho-alpha-mannosyl)-D-glycerate	1	0
C07185	Valproic acid	1	0
C06809	Acetylcysteine	1	0
C06868	Carbamazepine	1	0
C16565	Aminopropylcadaverine	1	0
C06428	Eicosapentaenoic acid	1	0
C00510	Oleoyl-CoA	1	0
C01607	Phytanic acid	1	0
C00527	Glutaryl-CoA	1	0
C08316	Erucic acid	1	0
C00212	Adenosine	1	0
C00257	Gluconic acid	1	0
C00189	Ethanolamine	1	0
C01606	Phthalic acid	1	0
C00473	Vitamin A	1	0
C00132	Methanol	1	0
C00745	L(-)-Nicotine	1	0
C00739	D-Lysine	1	0
C00155	Homocysteine	1	0
C00346	O-Phosphoethanolamine	1	0
C00357	N-Acetyl-D-Glucosamine 6-Phosphate	1	0
C00256	D-Lactic acid	1	0
C06427	Alpha-Linolenic acid	1	0
C08261	Azelaic acid	1	0
C04092	D-1-Piperideine-2-carboxylic acid	1	0
C00449	Saccharopine	1	0
C00408	Pipecolic acid	1	0
C00463	Indole	1	0
C06429	Docosahexaenoic acid	1	0
C08362	Palmitoleic acid	1	0
C00921	7,8-Dihydropteroic acid	1	0
C00415	Dihydrofolic acid	1	0

C00222	Malonic semialdehyde	1	0
C06866	Capsaicin	1	0
C01179	4-Hydroxyphenylpyruvic acid	1	0
C15767	4-(Glutamylamino) butanoate	1	0
C01013	Hydroxypropionic acid	1	0
C00253	Nicotinic acid	1	0
C00879	Galactaric acid	1	0
C00364	5-Thymidylic acid	1	0
C00106	Uracil	1	0
C01697	Galactitol	1	0
C00243	Alpha-Lactose	1	0
C04751	5-amino-1-(5-phospho-D-ribosyl)imidazole-4-c	1	0
C03373	5-Aminoimidazole ribonucleotide	1	0
C00186	L-Lactic acid	1	0
C00378	Thiamine	1	0
C11437	1-Deoxy-D-xylulose 5-phosphate	1	0
C00184	Dihydroxyacetone	1	0
C02154	Glyceraldehyde	1	0
C06195	Imidazolone	1	0
C00146	Phenol	1	0
C06424	Myristic acid	1	0
C03557	Ciliatine	1	0
C00777	All-trans-retinoic acid	1	0
C00823	1-Hexadecanol	1	0
C12269	N-Methyl-D-aspartic acid	1	0
C06501	O-Phosphotyrosine	1	0
C16527	Adrenic acid	1	0
C04144	Tetrahydropteroyltri-L-glutamate	1	0
C04489	5-Methyltetrahydropteroyltri-L-glutamate	1	0
C02291	L-Cystathionine	1	0
C00366	Uric acid	1	0
C00262	Hypoxanthine	1	0
C00081	Inosine triphosphate	1	0
C04823	SAICAR	1	0
C03090	5-Phosphoribosylamine	1	0
C03242	8,11,14-Eicosatrienoic acid	1	0
C06426	Gamma-Linolenic acid	1	0
C04257	N-Acetyl-D-mannosamine 6-phosphate	1	0
C00237	Carbon monoxide	1	0
C00424	Lactaldehyde	1	0
C00669	gamma-Glutamylcysteine	1	0
C03576	Mesna	1	0
C00719	Betaine	1	0
C00170	5'-Methylthioadenosine	1	0
C07588	Salicyluric acid	1	0
C00805	Salicylic acid	1	0
C01127	4-Hydroxy-2-oxoglutaric acid	1	0

C00349	2-Methyl-3-oxopropanoic acid	1	0
C06002	(S)-Methylmalonic acid semialdehyde	1	0
C00141	Alpha-ketoisovaleric acid	1	0
C00120	Biotin	1	0
C00683	Methylmalonyl-CoA	1	0
C04146	Octaprenyl diphosphate	1	0
C07086	Phenylacetic acid	1	0
C00618	3-Dehydro-L-gulonate	1	0
C13425	3-Hexaprenyl-4-hydroxybenzoic acid	1	0
C00633	4-Hydroxybenzaldehyde	1	0
C00931	Porphobilinogen	1	0
C01169	(S)-Succinyldihydrolipoamide	1	0
C00579	Dihydrolipoamide	1	0
C14732	5-KETE	1	0
C16513	Docosapentaenoic acid	1	0
C05361	Hydrazine	1	0
C00793	D-Cysteine	1	0
C00294	Inosine	1	0
C00329	Glucosamine	1	0
C01598	Melatonin	1	0
C00884	Homocarnosine	1	0
C00431	5-Aminopentanoic acid	1	0
C01042	N-Acetyl-L-aspartic acid	1	0
C16641	Irinotecan	1	0
C00376	Retinal	1	0
C00957	3-Mercaptopyruvic acid	1	0
C03017	Propionylcarnitine	1	0
C00300	Creatine	1	0
C04677	AICAR	1	0
C00581	Guanidoacetic acid	1	0
C00327	Citrulline	1	0
C01586	Hippuric acid	1	0
C08278	Suberic acid	1	0
C00230	Protocatechuic acid	1	0
C17349	Guanidine	1	0
C00580	Dimethylsulfide	1	0
C06672	Vanillic acid	1	0
C07443	Phenytoin	1	0
C00445	5,10-Methenyltetrahydrofolic acid	1	0
C01142	(3S)-3,6-Diaminohexanoate	1	0
C01419	Cysteinylglycine	1	0
C02218	2-Aminoacrylic acid	1	0
C00979	O-Acetylserine	1	0
C01008	Trimethyl sulfonium	1	0
C00153	Niacinamide	1	0
C04734	Phosphoribosyl formamidocarboxamide	1	0
C00664	5-Formiminotetrahydrofolic acid	1	0

C00697	Nitrogen	1	0
C01205	(R)-b-aminoisobutyric acid	1	0
C02226	Citraconic acid	1	0
C00213	Sarcosine	1	0
C07064	Lactulose	1	0
C01424	Gallic acid	1	0
C03541	Tetrahydrofolyl-[Glu](2)	1	0
C00365	dUMP	1	0
C01026	Dimethylglycine	1	0
C00818	Glucaric acid	1	0
C01216	2-Keto-3-deoxy-D-gluconic acid	1	0
C04442	2-Keto-3-deoxy-6-phosphogluconic acid	1	0
C02656	Pimelic acid	1	0
C01250	N-Acetyl-L-glutamate 5-semialdehyde	1	0
C04225	cis-2-Methylaconitate	1	0
C04133	N-Acetyl-L-glutamyl 5-phosphate	1	0
C20157	Beta-Carboline	1	0
C00954	Indoleacetic acid	1	0
C06337	Terephthalic acid	1	0
D00362	Lisinopril	1	0
C05672	2-Amino-3-phosphonopropionic acid	1	0
C01732	Mesaconic acid	1	0
C03406	Argininosuccinic acid	1	0
C05145	3-Aminoisobutanoic acid	1	0
C06860	Bupropion	1	0
C03765	4-Hydroxyphenylacetaldehyde	1	0
C04043	3,4-Dihydroxyphenylacetaldehyde	1	0
C00337	L-Dihydroorotic acid	1	0
C05598	Phenylacetylglycine	1	0
C02378	Aminocaproic acid	1	0
C00254	Prephenate	1	0
C00601	Phenylacetaldehyde	1	0
C00666	Diaminopimelic acid	1	0
C11143	Dimethyl sulfoxide	1	0
C00438	Ureidosuccinic acid	1	0
C03044	(2R,3R)-2,3-Butanediol	1	0
C01146	Tartronate semialdehyde	1	0
C00236	Glyceric acid 1,3-biphosphate	1	0
C00627	Pyridoxine 5'-phosphate	1	0
C01165	L-Glutamic gamma-semialdehyde	1	0
C00127	Oxidized glutathione	1	0
C00582	Phenylacetyl-CoA	1	0
C05979	Propyl alcohol	1	0
C00196	2-Pyrocatechuic acid	1	0
C00466	Acetoin	1	0
C03794	Adenylsuccinic acid	1	0
C02727	N6-Acetyl-L-lysine	1	0

C05840	Iminoaspartic acid	1	0
C01043	N-Carbamoylsarcosine	1	0
C08270	L-Canaline	1	0
C00308	Canavanine	1	0
C00971	4-Pyridoxolactone	1	0
C05936	N4-Acetylaminobutanal	1	0
C03340	L-2,3-Dihydrodipicolinate	1	0
C05396	Lactose 6-phosphate	1	0
C01186	(3S,5S)-3,5-Diaminohexanoate	1	0
C03451	S-Lactoylglutathione	1	0
C00990	5-Aminopentanamide	1	0
C01035	4-Guanidinobutanoic acid	1	0
C03771	2-Oxoarginine	1	0
C00940	2-Keto-glutaramic acid	1	0
C01259	3-Hydroxy-N6,N6,N6-trimethyl-L-lysine	1	0
C01149	4-Trimethylammoniobutanal	1	0
C01239	N-Acetyl-b-glucosaminylamine	1	0
C04540	Aspartylglycosamine	1	0
C15668	1-Pyrroline	1	0
C00531	Itaconyl-CoA	1	0
C01438	Methane	1	0
C03232	Phosphohydroxypyruvic acid	1	0
C00872	Aminomalonic acid	1	0
C01136	S-Acetyldihydrolipoamide	1	0

Table S2: S2 Node Table

Id	Label	Degree	Betweenness
C00022	Pyruvic acid	10	8.62
C00047	L-Lysine	9	6.87
C00049	L-Aspartic acid	9	2.99
C00004	NADH	8	14.9
C00037	Glycine	8	2.33
C00007	Oxygen	7	6.9
C00249	Palmitic acid	7	3.98
C00008	ADP	7	2.42
C00002	Adenosine triphosphate	6	1.5
C00334	Gamma-Aminobutyric acid	6	0.92
C02336	D-Fructose	4	0.97
C00332	Acetoacetyl-CoA	3	1.79
C01089	(R)-3-Hydroxybutyric acid	2	0.53
C01563	Carbamic acid	2	0.29

Table S3. Antibodies used for Immunofluorescence and Immunoblot Analysis

Antibody raised against	Host species	Dilution	Manufacturer	Catalog number
IGF-1R	Rabbit	1:1000	Abcam	Ab131476
IGF-1R	Rabbit	1:1000	Proteintech	20254-1-AP
Cre	Rabbit	1:1000	Novagen	69050
Rhodopsin	Mouse	1:1000	In-house	Gift from Dr. Jim McGinnis (OUHSC)
Rod-Transducin α	Rabbit	1:1000	Santa Cruz	SC-389
Rod-Arrestin	Mouse	1:1000<br>1:500 (IF)	In-house	Gift from Dr. Paul Hargrave (University of Florida)
M-opsin	Rabbit	1:1000<br>1:100 (IF)	Millipore Sigma	AB5405
Cone-Arrestin	Rabbit	1:1000	Millipore Sigma	AB15282
Actin	Mouse	1:1000	Thermo Fisher Scientific	MA1-744
Glutamine synthetase (GS)	Mouse	1:50 (IF)	Abcam	Ab64613
GFAP	Rabbit	1:100 (IF)	Dako	20334
