# Supplementary figures and images for: Insulin-like growth factor 1 receptor mediates photoreceptor neuroprotection
Source: Cell Death Dis. 2022 Jul 15;13(7):613. doi: 10.1038/s41419-022-05074-3 (PMC9287313; doi:10.1038/s41419-022-05074-3)

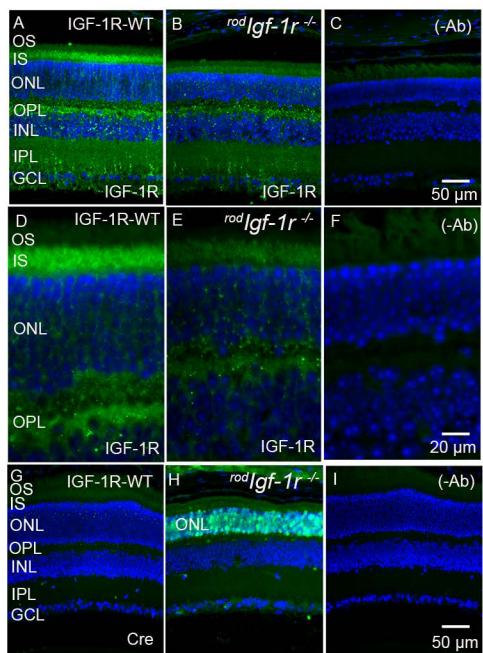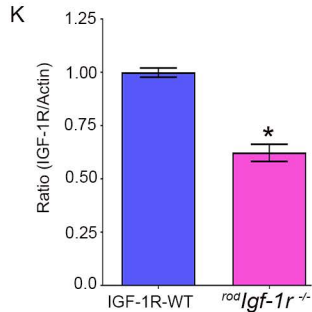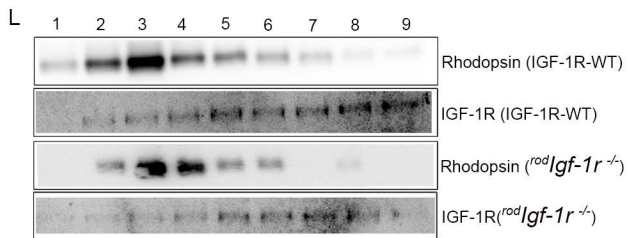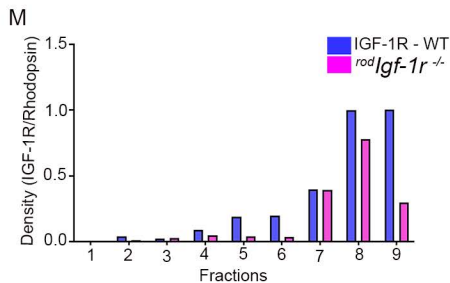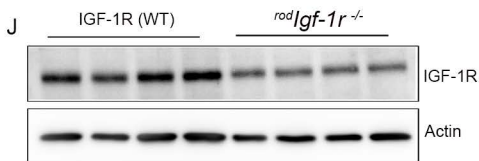

Figure 1

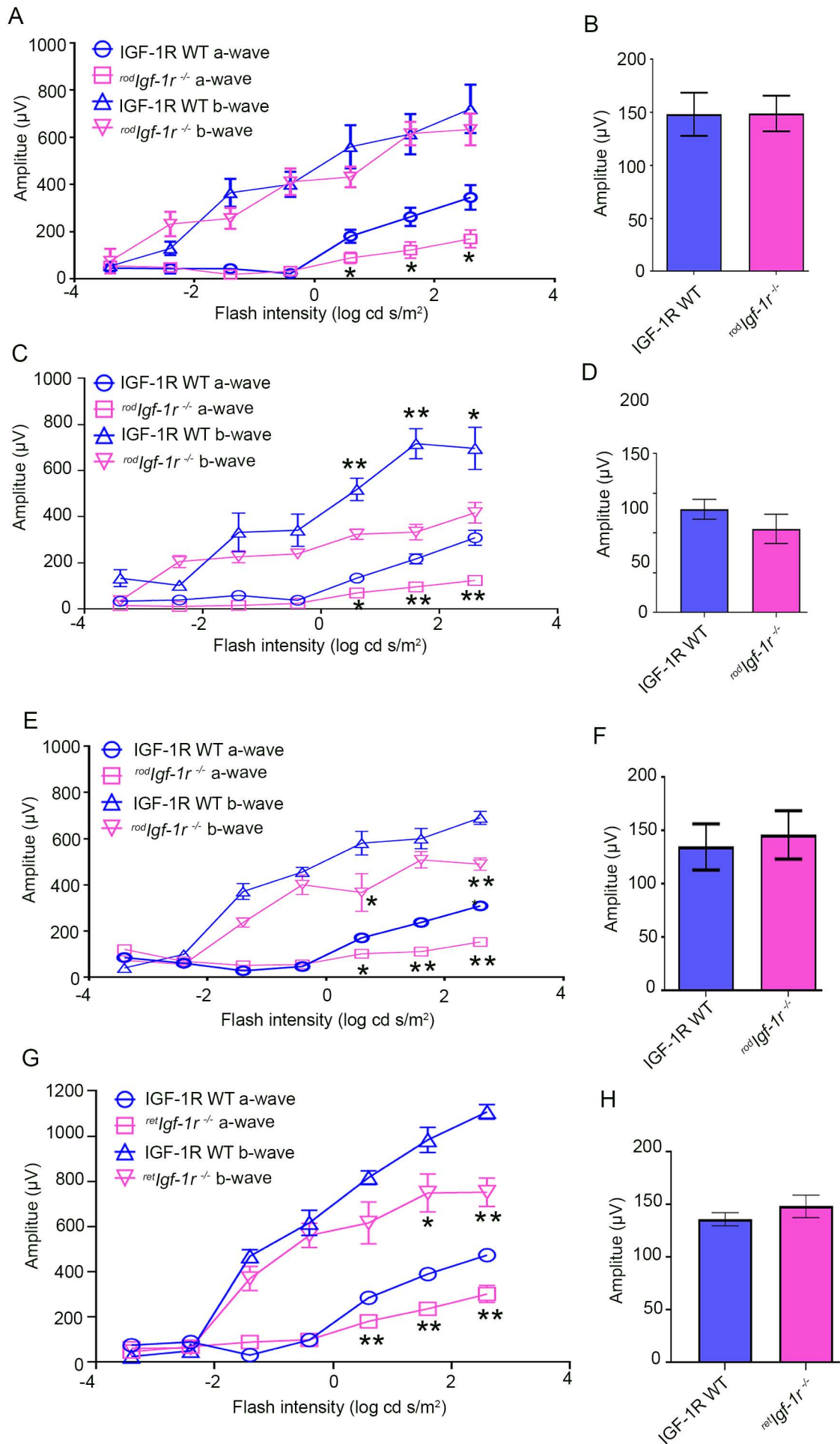

Figure 2

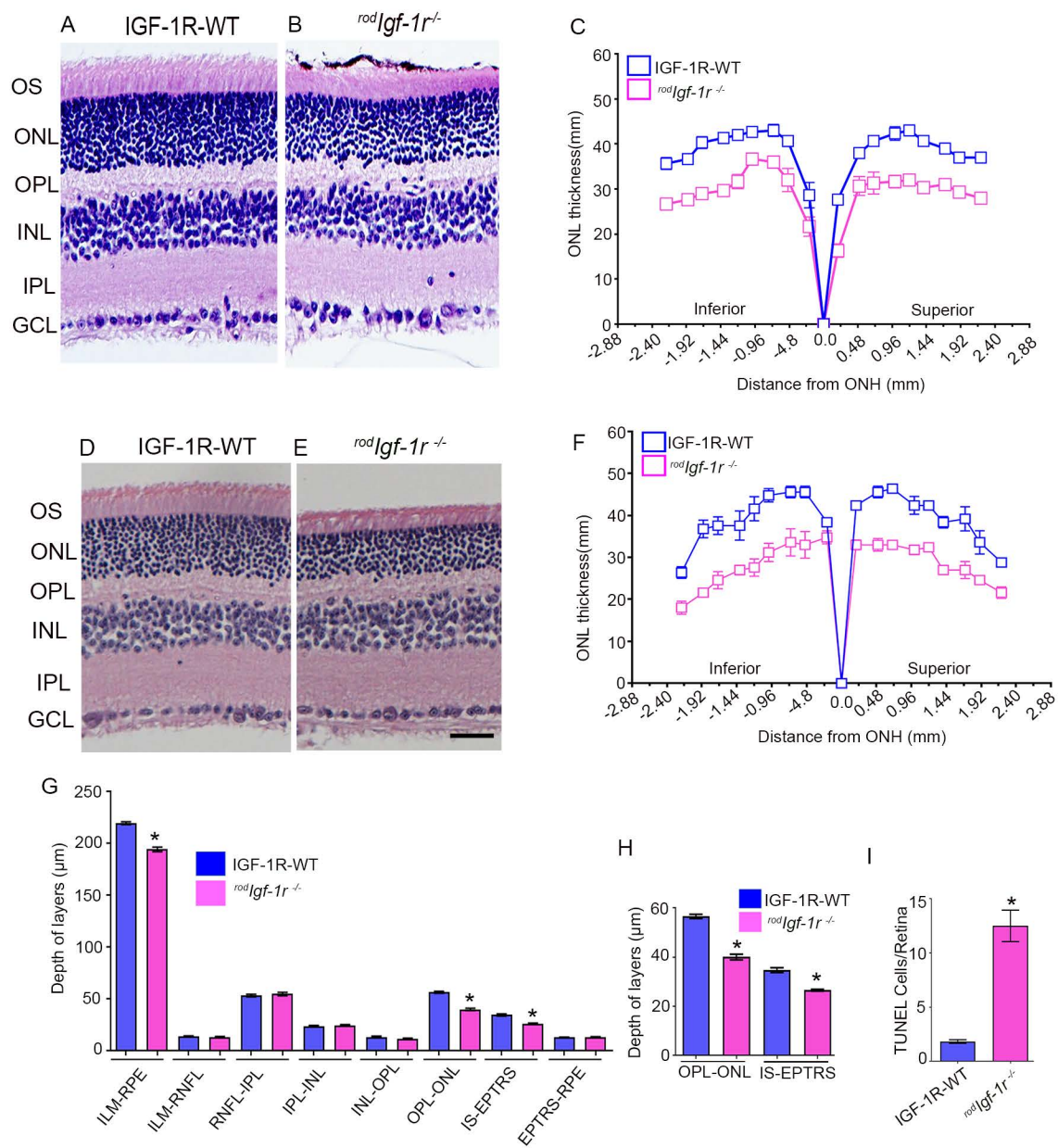

Figure 3

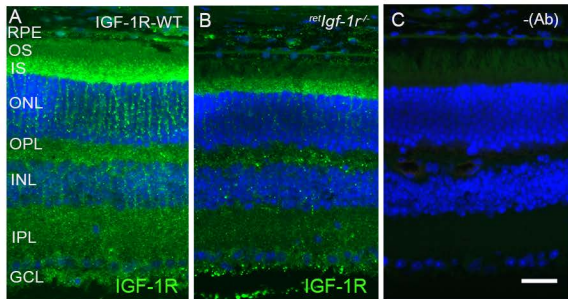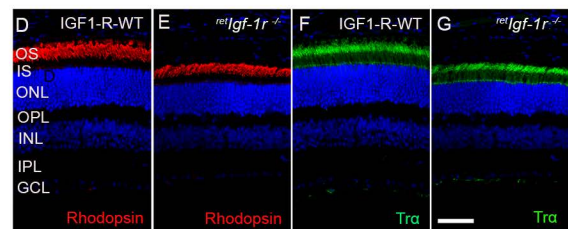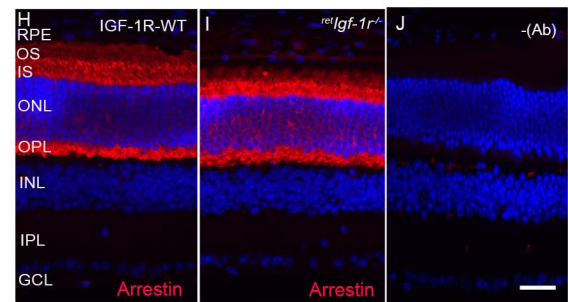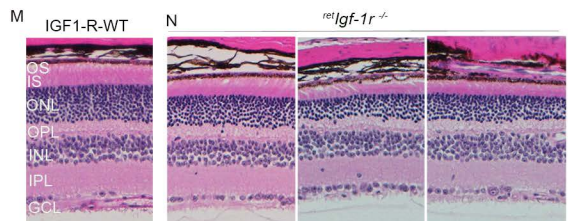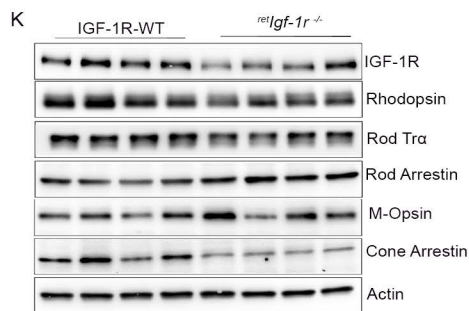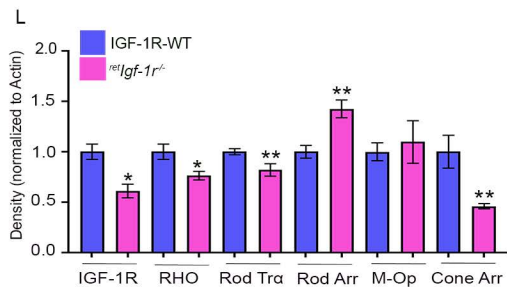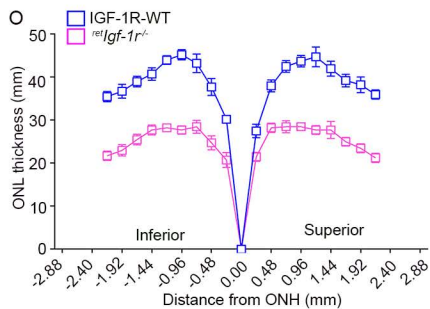

Figure 4

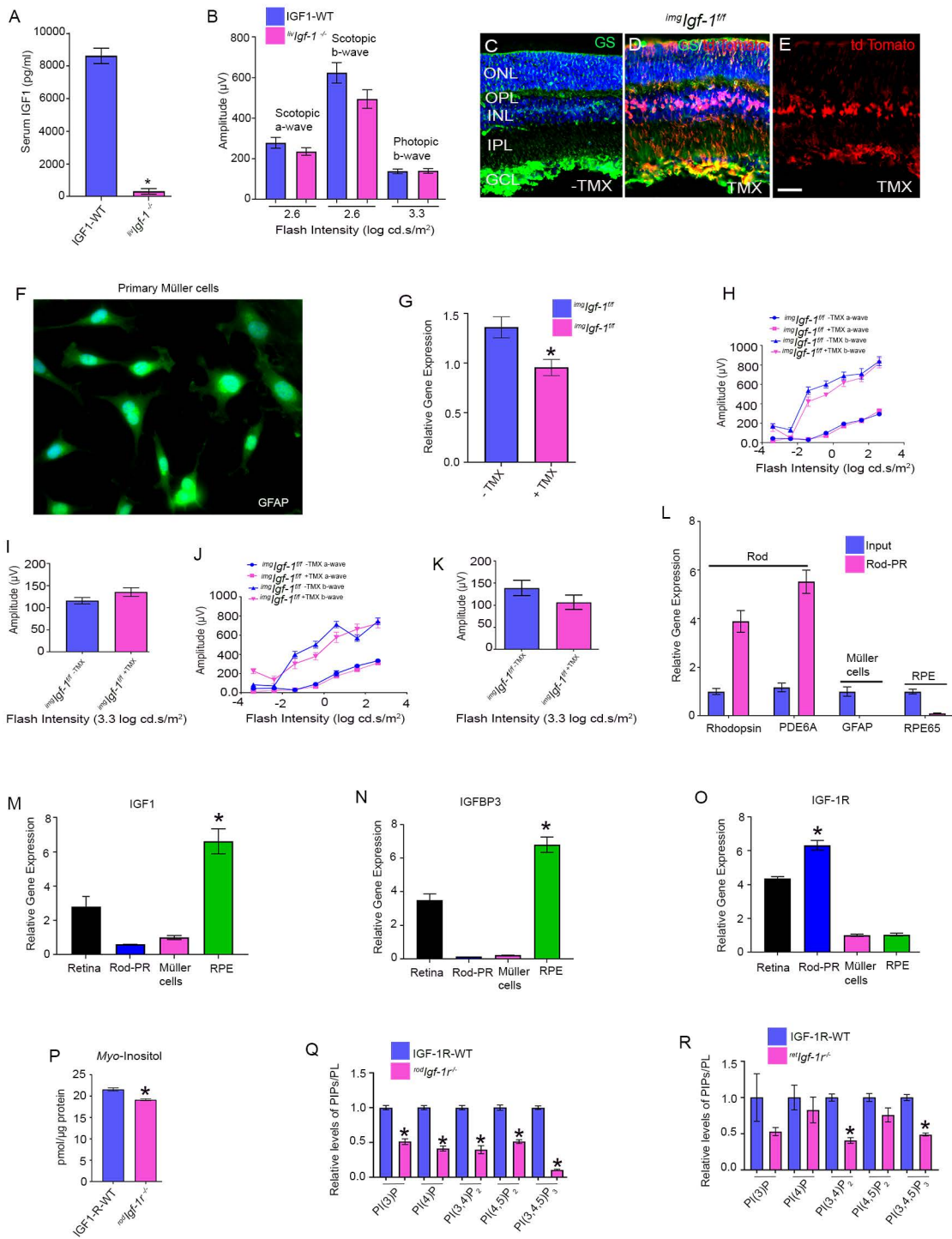

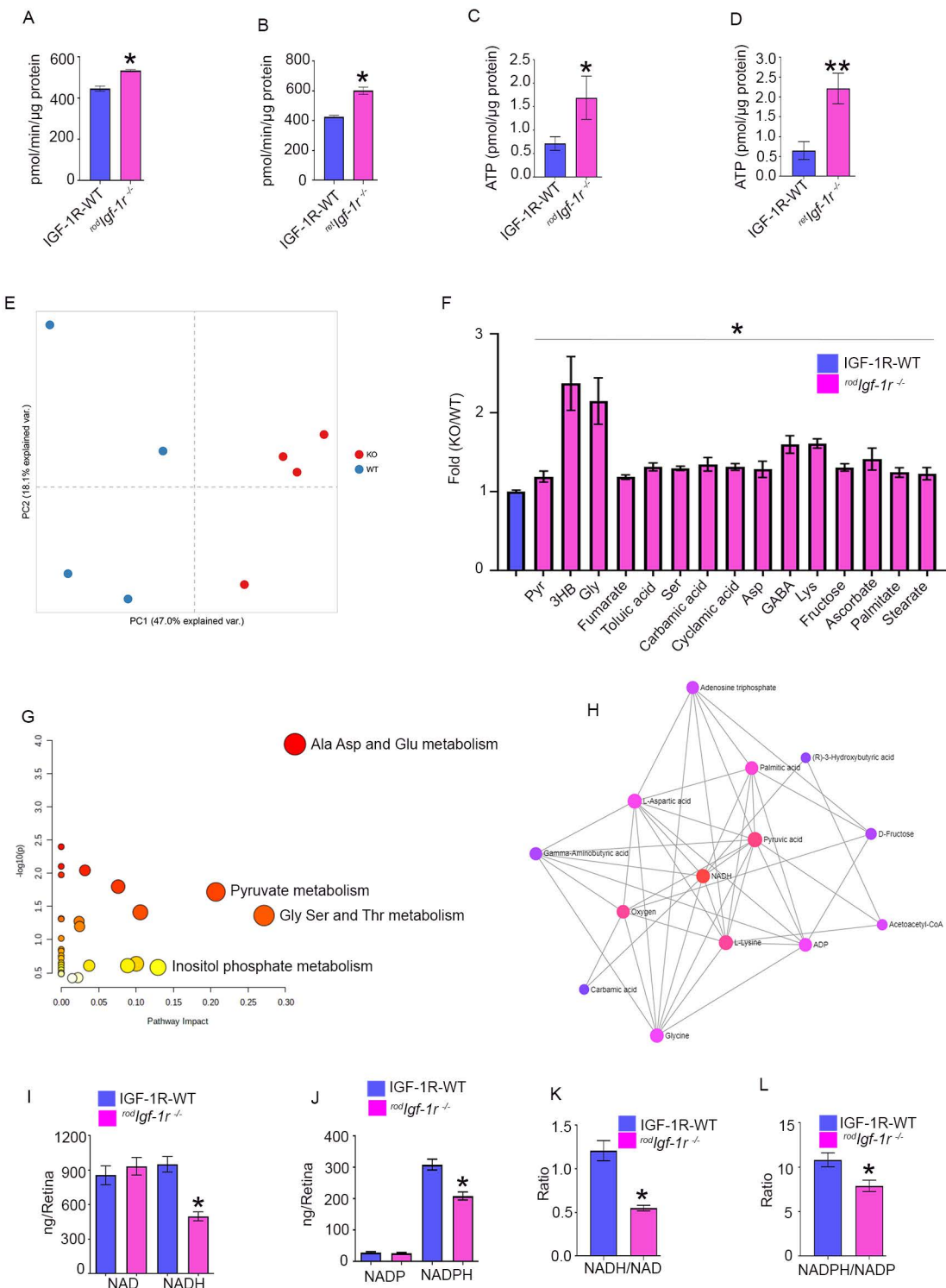

Figure 6

A

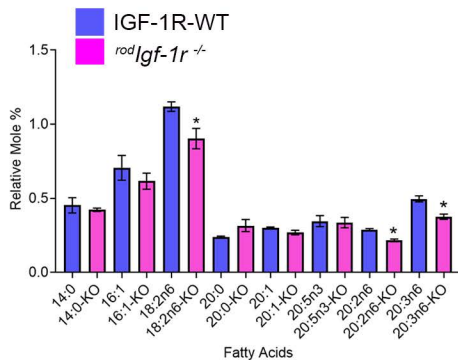

B

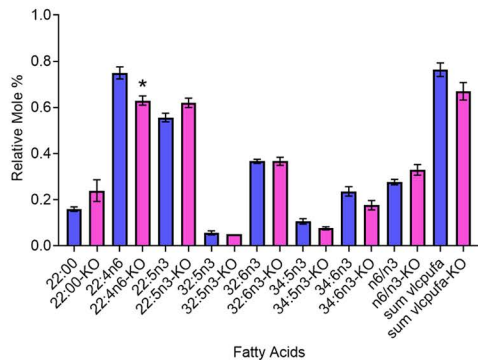

C

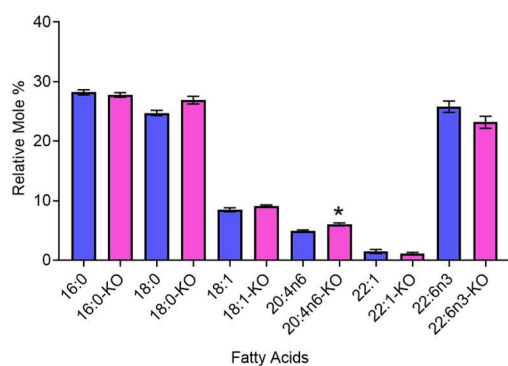

D

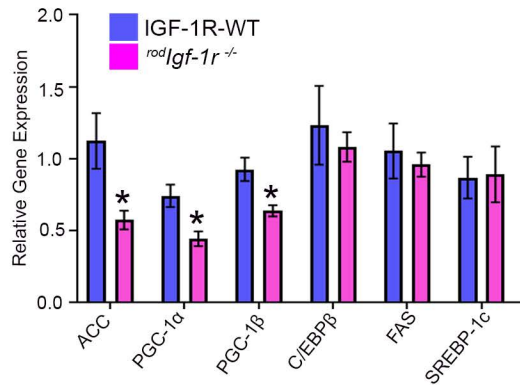

E

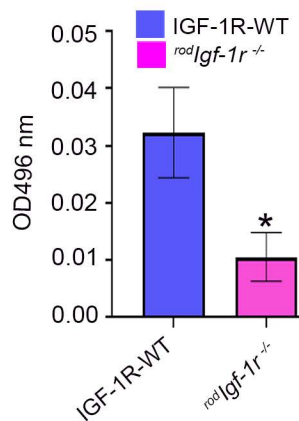

Figure 7

Supplement: Supplementary file 5 — Merged Figures File [file 41419_2022_5074_MOESM5_ESM.pdf]
